# Therapeutic approaches for difficult-to-treat rheumatoid arthritis: a systematic literature review of current evidence

**DOI:** 10.1136/rmdopen-2026-006860

**Published:** 2026-08-04

**Authors:** Golnaz Shams, András Miklós Dorgó, Chiara Ripepi, Emma Wettersand, Ewa Malczuk, Caroline Neumeister, Slađana Rumpl Tunjić, Brigitte Wildner, Hendrik Schulze-Koops, György Nagy, João Eurico Fonseca, Jacob M van Laar, Paco M J Welsing, Paul Studenic

**Affiliations:** 1 Department of Internal Medicine 3, Division of Rheumatology, Medical University of Vienna, Vienna, Austria; 2 Department of Rheumatology and Immunology, Semmelweis University, Budapest, Hungary; 3 Department of Rheumatology & Clinical Immunology, University Medical Center Utrecht, Utrecht, The Netherlands; 4 Rheumatology, Theme Inflammation and Ageing, Karolinska University Hospital, Stockholm, Sweden; 5 Rheumatology division, Department of Medicine Solna, Karolinska Institutet, Stockholm, Sweden; 6 Department of Internal Diseases and Rheumatology, Military Institute of Medicine—National Research Institute, Warsaw, Poland; 7 Association Remisija, EULAR PARE Patient Research Partner, Zagreb, Croatia; 8 University Library, Medical University of Vienna, Vienna, Austria; 9 Division of Rheumatology and Clinical Immunology, Ludwig Maximilians University Munich, Munich, Germany; 10 Heart and Vascular Center, Semmelweis University, Budapest, Hungary; 11 Semmelweis University, Department of Genetics, Cell- and Immunobiology, Budapest, Hungary; 12 Medical School, University of Lisbon and ULS Santa Maria; Lisbon Academic Medical Center, Lisbon, Portugal

**Keywords:** Arthritis, Rheumatoid, DMARD, Biological Therapy, Risk Factors, Health-Related Quality Of Life

## Abstract

**Objective:**

This systematic literature review (SLR) aims to update the evidence regarding therapeutic strategies in difficult-to-treat rheumatoid arthritis (D2T RA), building on the previous SLR informing the European Alliance of Associations for Rheumatology (EULAR) points to consider for the management of D2T RA.

**Methods:**

Three research questions addressed efficacy or safety of treatments in patients with RA with (1) active disease and limited treatment options; (2) active disease with ≥2 prior biologic/targeted synthetic disease-modifying antirheumatic drugs (b/tsDMARDs) and (3) poor health-related quality of life and low objective disease activity (non-pharmacological interventions). MEDLINE, Embase and Cochrane Library were searched until July 2025. Meta-analysis and meta-regression were conducted.

**Results:**

We screened 8589 records and included 131 studies. For research question (RQ)1, evidence on DMARD efficacy and safety was synthesised across a wide spectrum of comorbidities including obesity, cardiovascular disease, history of malignancy and respiratory comorbidities. For RQ2, all b/tsDMARDs except otilimab demonstrated better efficacy than placebo (mostly high risk of bias). Meta-regression showed efficacy was maintained for Janus kinase inhibitors (JAKi) despite increasing number of prior bDMARD failures (1 to ≥3). Safety results confirmed the increased occurrence of infection and malignancy with JAKi. For RQ3, limited evidence suggested orthopaedic surgical intervention as a potential non-pharmacological option in patients with D2T RA.

**Conclusions:**

This SLR summarised evidence supporting DMARD efficacy/safety across multiple comorbidities. In patients with active RA with ≥2 prior bDMARDs, JAKi offer substantial clinical benefits but require careful risk stratification given cardiovascular and malignancy risks. Furthermore, standardised reporting and dedicated studies on non-pharmacological interventions are urgently needed.

**PROSPERO registration number:**

CRD42024593584.

**WHAT IS ALREADY KNOWN ON THIS TOPIC**

- Difficult-to-treat rheumatoid arthritis (D2T RA) is a heterogeneous, multifactorial state defined by European Alliance of Associations for Rheumatology (EULAR) (treatment failures plus active/progressive disease and problematic disease management), and the earlier evidence base informing recommendations was scarce and often indirect.

**WHAT THIS STUDY ADDS**

- This systematic literature review (searches updated to July 2025) provides a synthesis of therapeutic evidence across three D2T RA-relevant clinical scenarios, addressing patient populations who have: limited treatment options due to comorbidity or contraindication; persistent active disease after ≥2 prior biologic/targeted synthetic disease-modifying antirheumatic drug (b/tsDMARD) failures and poor health-related quality of life despite low objective inflammatory activity, considered for non-pharmacological interventions.
- We have provided an expanded synthesis of DMARD safety across RA patient populations with a range of comorbidities or treatment contraindications.
- In patients with active disease and ≥2 prior bDMARDs, JAK inhibitors showed efficacy versus placebo and appeared to maintain relative efficacy across increasing numbers of prior bDMARD failures, although the evidence derives from subgroup analyses of randomised controlled trials mostly with high risk of bias.
- Evidence for non-pharmacological approaches in b/tsDMARD-exposed patients with low inflammatory activity remains very limited, highlighting a major gap in D2T RA care research.

**HOW THIS STUDY MIGHT AFFECT RESEARCH, PRACTICE OR POLICY**

- The review supports individualised treatment decision-making in D2T RA, including cautious consideration of JAK inhibitors balanced against their cardiovascular and malignancy risk profile, in refractory disease and careful tailoring of DMARD choices in the presence of comorbidities.
- Future trials should prespecify D2T RA-relevant subgroup definitions (at least incorporating ≥2 prior b/tsDMARD exposures with different mechanisms of action).
- Dedicated studies on non-pharmacological strategies in well-defined populations with persistent symptoms will be essential to shape future research agendas in D2T RA.

## Introduction

 Rheumatoid arthritis (RA) is a heterogeneous chronic rheumatic disease, with its hallmark symptoms of synovitis accompanied by pain and a plethora of other symptoms impacting quality of life.^1 2^ The introduction of the treat-to-target concept, combined with an increased number of disease-modifying antirheumatic drugs (DMARDs) with different mechanisms of action (MoA) becoming available, has improved outcomes and prospects for patients with RA.^3 4^ A considerable proportion of patients, however, do not achieve sustained remission or low disease activity (LDA) despite the availability of multiple biologic and targeted synthetic DMARDs (b/tsDMARDs).^5^ Clinical reality shows that some patients remain symptomatic despite trialling several b/tsDMARDs. To better harmonise research and care of this subgroup, a task force of the European Alliance of Associations for Rheumatology (EULAR) proposed a definition of difficult-to-treat RA (D2T RA) in 2021.^6^ This umbrella definition made a conceptual shift from a purely treatment-refractory state of the disease to a broader, multifactorial understanding of insufficient disease control. It consists of three main components that must all be present: previous treatment failures, signs of active/progressive disease and problematic perception of disease management by the patient and/or physician. Importantly, patients with well-controlled disease according to validated measures but with persistent RA symptoms that reduce their quality of life also fulfil the criterion regarding active/progressive disease for this definition. Consequently, the D2T RA designation represents a highly heterogeneous population, encompassing a diverse spectrum of clinical phenotypes, underlying biological mechanisms and varied reasons for treatment failure. This ranges from those with true treatment-refractory (multidrug-resistant) disease to others whose persistent symptoms might be amplified by comorbidities that may also limit their treatment options, or non-inflammatory mechanisms such as pain sensitisation or fatigue that preclude effective disease control directed at further lowering inflammatory disease activity.^7 8^ In parallel to the D2T RA definition of EULAR, a comprehensive systematic literature review (SLR) was conducted in 2020 to inform the task force on the EULAR recommendations for the management of D2T RA. Results underscored the scarcity and heterogeneity of the available evidence, which was indeed indirect for this population.^9 10^ Subsequently, several studies have expanded the understanding of D2T RA, emphasising the role of comorbidities,^11^ reporting a global pooled prevalence of 11.7%,^12^ highlighting psychological factors, pain sensitisation and socioeconomic disadvantage as key contributors^13^ and positioning D2T RA within a broader D2T framework across rheumatology.^14^ Given that the earlier SLR^9^ necessarily applied less stringent inclusion criteria and the evidence base has since expanded with new studies reporting on populations that more directly meet the D2T RA definition, the present review builds on the previous SLR^9^ by applying inclusion criteria better aligned with the EULAR definition, enabling more consistent identification of D2T RA populations across studies. Through this approach, we aim to provide a comprehensive synthesis of the available evidence on management strategies for D2T RA subpopulations. The current SLR is independent of the previous EULAR task force and was conducted as part of Stratification of Rheumatoid Arthritis: CompuTational models to personalise mAnagement strategies for difFIcult-to-Treat disease (STRATA-FIT), the first Horizon Europe-funded consortium dedicated to D2T RA. Its findings will serve as the basis for developing a stratified, personalised treatment strategy for patients with D2T RA, which will be tested in the prospective interventional pilot study within the STRATA-FIT project.

## Methods

This SLR was conducted in line with the Cochrane Handbook for Systematic Reviews of Interventions and reported following the Preferred Reporting Items for Systematic Reviews and Meta-Analyses (PRISMA) guidelines.^15 16^ To ensure methodological transparency, the review protocol was prospectively registered with PROSPERO (CRD42024593584). One patient research partner (SRT) was involved from the conceptual phase throughout the entire SLR, including participation in project meetings during protocol development, screening strategy, conflict resolution, discussion and interpretation of the results.

### Research questions

The research questions and accompanying framework of the current SLR were derived based on those investigated in the EULAR D2T RA SLR^9^ through iterative online meeting discussions of a subgroup of investigators (AMD, CN, CR, EW, PS, PW, SRT). The eligibility criteria were refined to more closely operationalise the EULAR definition of D2T RA (online supplemental table 5). Accordingly, the adapted research questions address three distinct clinical scenarios: (1) efficacy and safety of treatments in patients with active RA but limited treatment options due to comorbidities, contraindications or drug-drug interactions; (2) efficacy and safety of treatments in patients with persistent disease activity after failure of ≥2 prior b/tsDMARDs and (3) efficacy of non-pharmacological interventions in patients with RA with poor health-related quality of life (HRQoL) and low objective markers of disease activity. The detailed research questions, structured in Population, Intervention, Comparator and Outcomes (PICO) format, are provided in online supplemental tables 2-4.

### Search strategy

Literature searches were conducted in MEDLINE (R) ALL (via Ovid), Embase (via Embase.com) and the Cochrane Central Register of Controlled Trials (CENTRAL) (via Ovid) by an experienced librarian from the Medical University of Vienna (BW) for the period from 1 December 2019 (the data cut-off of the 2021 SLR) to 12 September 2024. Search terms for rheumatoid arthritis, difficult-to-treat, comorbidities and outcomes were applied as both database-specific subject headings and free-text terms. Truncation and proximity operators were used, with restrictions to specific database fields where appropriate. To ensure comprehensive coverage, four complementary search approaches were combined into a single strategy addressing the overall topic and predefined research questions. The search was updated on 9 July 2025 to capture more recent publications. Additionally, hand-searching of relevant literature was performed. The complete search strategies for all databases are provided in the online supplemental appendix 2.

### Selection, data extraction and quality assessment

We sought to capture evidence from studies including a D2T RA population and indirect evidence from subgroups within broader RA studies that reflect the D2T RA population (table 1). For PICO 1, we examined studies assessing management options for patients with active RA in whom comorbidities, contraindications or drug-drug interactions restrict therapeutic choices, contributing to a D2T state. Because studies including RA populations with comorbidities do not necessarily reflect this subgroup, additional eligibility criteria were applied. Specifically, studies were excluded if they had patients with RA in remission or LDA or patients who were conventional synthetic DMARD-naïve. Randomised controlled trials (RCTs), cohort, case-control and long-term extension (LTE) studies, with ≥40 participants, were eligible for inclusion. For PICO 2, we focused on treatment-refractory disease, comprising patients with RA with active disease who failed at least two b/tsDMARDs. Treatment failure includes failure due to either lack of efficacy or intolerance. We did not specifically require two different MoAs so as not to miss relevant evidence. For efficacy outcomes, only RCTs were eligible, while for safety outcomes, RCTs, cohort, case-control and LTE studies with ≥40 participants and a comparator group from the same population were eligible for inclusion. For PICO 3, the focus was on patients with RA with predominantly non-inflammatory complaints with no design requirement. Eligibility criteria excluded studies conducted exclusively in populations with high disease activity. At least one current or prior b/tsDMARD use was required. Studies had to evaluate non-pharmacological interventions with ≥30 participants.

**Table 1:** Operationalisation of PICO scenarios relative to EULAR D2T RA components

EULAR D2T RA component	EULAR D2T RA definition	PICO 1: efficacy and safety of treatments in patients with active RA but limited treatment options due to comorbidities, contraindications or drug-drug interactions	PICO 2: efficacy and safety of treatments in patients with persistent disease activity after failure of ≥2 prior b/tsDMARDs	PICO 3: efficacy of non-pharmacological interventions in patients with RA with poor health-related quality of life and low objective markers of disease activity
1. Previous treatment failures	Failure of ≥2 b/tsDMARDs (with different mechanisms of action)* after failing csDMARD therapy (unless contraindicated).†	Prior treatment with at least one csDMARD (ie, not csDMARD-naïve).	Prior failure of at least two b/tsDMARDs, regardless of mechanism of action, due to lack of efficacy or intolerance.	Treatment with at least one b/tsDMARD, either currently or previously.
2. Signs suggestive of active/progressive disease (≥1 of a–e)	At least moderate disease activity (according to validated composite measures including joint counts, eg, DAS28-ESR >3.2 or CDAI >10). Signs (including acute phase reactants and imaging) and/or symptoms suggestive of active disease (joint related or other). Inability to taper glucocorticoid treatment (below 7.5 mg/day prednisone or equivalent). Rapid radiographic progression (with or without signs of active disease).‡ Well-controlled disease according to above standards, but still having RA symptoms that are causing a reduction in quality of life.	Criterion 2a was the main operational criterion; criteria 2b, 2c and 2d were allowed but not required.	Criterion 2a was the main operational criterion; criteria 2b, 2c and 2d were allowed but not required.	Criterion 2e was required; studies exclusively including patients with high disease activity were not eligible.
3. Problematic perception of disease management	The management of signs and/or symptoms is perceived as problematic by the rheumatologist and/or the patient.	–	–	–

All three criteria need to be present in D2T RA.

* Unless restricted by access to treatment due to socioeconomic factors.

† Treatment according to EULAR recommendation and if csDMARD treatment is contraindicated, failure of ≥2 b/tsDMARDs with different mechanisms of action is sufficient.

‡ Rapid radiographic progression: change in van der Heijde-modified Sharp score ≥5 points at 1 year.

b, biological; CDAI, Clinical Disease Activity Index; cs, conventional synthetic; DAS28-ESR, Disease Activity Score assessing 28 joints using erythrocyte sedimentation rate; DMARD, disease-modifying antirheumatic drug; D2T, difficult-to-treat; EULAR, European Alliance of Associations for Rheumatology; PICO, Population, Intervention, Comparator and Outcomes; RA, rheumatoid arthritis; ts, targeted synthetic.

Titles and abstracts were screened in duplicate (initial search: AMD, CR, EW; updated search: AMD, GS). Full texts of potentially eligible studies were also screened in duplicate (initial search: AMD, CR, EW, GS; updated search: AMD, GS). At both stages, conflicts were resolved in consultation with the methodologists (PW and PS). In addition, full-text rescreening of studies from the previous SLR was conducted by CN and GS to ensure alignment with the current eligibility criteria. The screening process was conducted via the Rayyan platform.^17^

Data were extracted by EM and GS using a predefined data extraction sheet developed for this review. For every article, key study characteristics were recorded, including author, year of publication, trial identifier (if applicable) and study design. Information relevant to the review questions was collected, covering patient characteristics, interventions, comparators and selected outcomes. For RCTs, risk of bias (RoB) was assessed using the Cochrane Risk of Bias (RoB V.2.0) tool.^18^ The overall RoB was classified as low, some concerns or high, based on the highest level of risk identified across the five domains. The Newcastle-Ottawa Scale was used to assess quality scores for observational studies.^19^

### Statistical analyses

The extracted data were summarised. Categorical outcomes were reported as frequency with proportions and continuous outcomes as means with 95% CIs. Where available, effect estimates, including risk ratios (RRs), incidence rate ratios (IRRs), ORs, HRs or mean differences, were reported with 95% CIs and p values. RRs with 95% CIs were calculated and graphically summarised for efficacy outcomes comparing a specific active treatment versus placebo. Random-effects meta-analyses were performed using the Mantel-Haenszel method when deemed possible based on study characteristics and the variability of effect estimates. Mixed-effects meta-regression using restricted maximum likelihood estimation was conducted to explore whether the number of prior bDMARD treatment failures modified treatment efficacy. The number of prior bDMARD failures was analysed as a binary variable based on the categories reported in the included studies (1 vs ≥2 prior failures; ≥2 vs ≥3 prior failures). The Knapp-Hartung adjustment was applied to provide more accurate CIs and p values. All analyses were conducted in R (V.4.4.1; R Core Team, Vienna, Austria)^20^ using the meta and metafor packages.^20 21^

## Results

**Figure 1:**
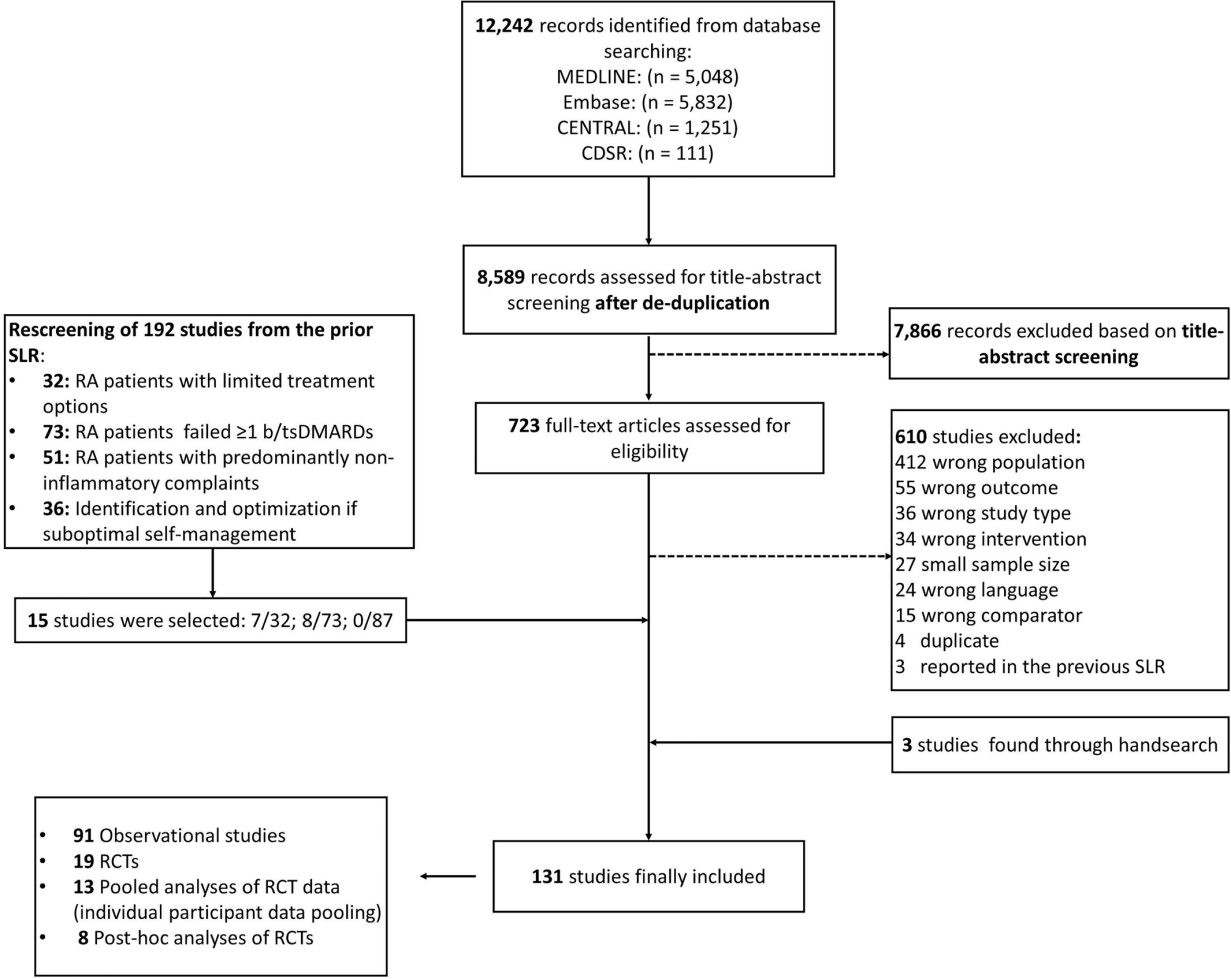
Preferred Reporting Items for Systematic Reviews and Meta-Analyses (PRISMA) flow chart of study selection process. b/tsDMARD, biological/targeted synthetic disease-modifying antirheumatic drug; CDSR, Cochrane Database of Systematic Reviews; RA, rheumatoid arthritis; RCT, randomised controlled trial; SLR, systematic literature review.

The search yielded 8589 records from EMBASE, MEDLINE and the Cochrane Library after deduplication. Following title and abstract screening, 723 references were assessed in full text, of which 113 studies met the inclusion criteria for one of the PICOs. Additionally, three relevant studies were included through manual search. In addition, 15 studies were identified through the rescreening of papers selected from the previous SLR (7/32, 8/73 and 0/87 for PICO 1, 2 and 3, respectively). Reasons for non-eligibility were mostly wrong study type or small sample size, too broad population, including studies involving patients who had failed ≥1 b/tsDMARD and no relevant treatment history. Figure 1 provides the PRISMA flow chart, outlining the overview of the literature search and study selection process. In total, 131 articles were finally included (107 for PICO 1, 21 for PICO 2 and three for PICO 3), of which 116 were newly published and 15 were identified through rescreening of the prior SLR. Studies comprised 19 RCTs, eight post hoc analyses of RCTs, 13 pooled analyses of RCT data (individual participant data pooling) and 91 observational studies. For PICO 2, all RCTs contributed data through subgroup analyses. Four studies explicitly applied the EULAR D2T RA definition as inclusion criteria. Detailed characteristics of included studies and RoB assessments are provided in online supplemental tables 6-12.

### Patients with RA with active disease, prior DMARD exposure and limited treatment options

A total of 107 studies including seven RCTs (six high RoB, one some concerns), seven post hoc analyses of RCTs, 12 pooled analyses of RCT data and 81 observational studies (46 high, 31 moderate and four low RoB) were identified, covering a wide spectrum of comorbidities including cardiovascular disease (CVD),^22–26^ diabetes mellitus,^22 27–31^ obesity,^32–42^ metabolic syndrome,^43^ osteoporosis,^44–49^ renal insufficiency and chronic kidney disease,^22 50–56^ hepatitis B virus and hepatitis C virus infection,^57–67^ history of malignancy,^68–74^ pulmonary disorders,^31 75–78^ RA-associated interstitial lung disease (RA-ILD),^76 79–103^ latent tuberculosis infection,^104 105^ Sjögren’s disease,^106^ amyloid A amyloidosis secondary to RA,^107^ mental disorders,^108 109^ anaemia^22^ and high comorbidity burden.^34^ Treatment-limiting factors included a history of infection,^110–112^ antidrug antibody positivity^113^ and cardiovascular (CV) risk factors in patients initiating Janus kinase inhibitors (JAKi).^24–26 112 114–127^ The results are presented in online supplemental table 1 in detail. Studies with reports on treatment effect estimates comparing treatment outcomes in this population are presented in table 2. More frequently studied co-existing conditions with more consistent evidence on treatment effects are described in greater detail in the sections below on malignancy and cancer, respiratory comorbidities, metabolic syndrome, and CV risk factors.

**Table 2:** Studies reporting effect estimates in patients with RA with active disease and comorbidities or contraindication to certain DMARDs

Comorbidity/Limitation	Comparison groups	Outcome	Effect estimate	RoB		
				L	M/SC	H
Prior malignancy^68^	ABA (with prior malignancy vs without)	Incident malignancy	aHR (95% CI) 0.994 (0.134 to 7.367)			–
Recently diagnosed breast cancer*, ^70^	TNFi versus csDMARDs	Overall survival	aHR (95% CI) 0.77 (0.42 to 1.40)		!	
	TNFi versus csDMARDs	Overall survival	aHR (95% CI) 0.84 (0.54 to 1.31)		!	
Prior solid cancer (excluding NMSC)^74^	TNFi versus csDMARDs	Cancer recurrence	aHR (95% CI) 1.10 (0.21 to 3.16)	+		
	RTX versus csDMARDs	Cancer recurrence	aHR (95% CI) 0.94 (0.32 to 2.11)	+		
Prior breast cancer^74^	TNFi versus csDMARDs	Cancer recurrence	aHR (95% CI) 0.68 (0.00 to 2.37)	+		
	RTX versus csDMARDs	Cancer recurrence	aHR (95% CI) 0.52 (0.00 to 1.95)	+		
Prior breast cancer^71^	TNFi versus no bDMARDs	Overall survival	aHR (95% CI) 1.40 (0.42 to 4.73)			–
	Non-TNFi† versus no bDMARDs	Overall survival	aHR (95% CI) 1.37 (0.22 to 8.42)			–
Prior solid malignancies (excluding NMSC)^71^	TNFi versus no bDMARDs	Overall survival	aHR (95% CI) 0.67 (0.31 to 1.44)			–
	Non-TNFi† versus no bDMARDs	Overall survival	aHR (95% CI) 1.10 (0.26 to 4.60)			–
Pior malignancy^72^	JAKi versus TNFi	Incident cancer‡	aIRR (95% CI) 0.7 (0.2 to 2.7)		!	
	RTX versus TNFi	Incident cancer‡	aIRR (95% CI) 0.3 (0.1 to 1.3)		!	
	IL-6Ri versus TNFi	Incident cancer‡	aIRR (95% CI) 1.7 (0.6 to 4.3)		!	
	ABA versus TNFi	Incident cancer‡	aIRR (95% CI) 1.9 (0.8 to 4.7)		!	
Recently diagnosed colorectal cancer^73^	TNFi versus csDMARDs	Overall survival	aHR (95% CI) 0.72 (0.43 to 1.21)		!	
Recently diagnosed lung cancer^73^	TNFi versus csDMARDs	Overall survival	aHR (95% CI) 0.70 (0.49 to 1.00)		!	
Recently diagnosed prostate cancer^73^	TNFi versus csDMARDs	Overall survival	aHR (95% CI) 0.80 (0.44 to 1.44)		!	
RA-ILD^94^	TNFi versus RTX	All-cause mortality	**aHR (95% CI) 2.33 (1.05 to 5.13**)			–
RA-ILD^90^	csDMARDs versus TNFi	All-cause mortality	aHR (95% CI) 0.90 (0.42 to 1.94)	+		
	IL-6Ri versus TNFi	All-cause mortality	aHR (95% CI) 0.62 (0.26 to 1.51)	+		
	ABA versus TNFi	All-cause mortality	aHR (95% CI) 0.74 (0.36 to 1.53)	+		
	RTX versus TNFi	All-cause mortality	aHR (95% CI) 0.56 (0.28 to 1.12)	+		
	JAKi versus TNFi	All-cause mortality	aHR (95% CI) 0.78 (0.28 to 2.19)	+		
	(IL-6Ri, ABA, RTX and JAKi) versus TNFi	All-cause mortality	**aHR (95% CI) 0.56 (0.33 to 0.97**)	+		
RA-ILD^101^	JAKi versus TNFi	All-cause mortality	**aHR (95% CI) 1.46 (1.136 to 1.870**)		!	
RA-ILD^99^	Non-TNFi versus TNFi	Death or respiratory hospitalisation	aHR (95% CI) 1.21 (0.92 to 1.58)		!	
RA-ILD^100^	ABA versus RTX	Death or respiratory hospitalisation	aHR (95% CI) 1.03 (0.72 to 1.47)		!	
	TCZ versus RTX	Death or respiratory hospitalisation	aHR (95% CI) 1.15 (0.68 to 1.93)		!	
	TOFA versus RTX	Death or respiratory hospitalisation	aHR (95% CI) 0.89 (0.54 to 1.46)		!	
RA-ILD^76^	ABA versus TNFi	ILD exacerbation§	aIRR (95% CI) 0.44 (0.18 to 1.09)		!	
COPD^76^	ABA versus TNFi	COPD exacerbation§	aIRR (95% CI) 0.91 (0.80 to 1.03)		!	
COPD^78^	ABA versus other bDMARDs	Hospitalised COPD exacerbation	aHR (95% CI) 0.57 (0.33 to 0.99)			–
Asthma^76^	ABA versus TNFi	Asthma exacerbation§	aIRR (95% CI) 0.81 (0.54 to 1.22)		!	
Bronchiectasis^75^	RTX versus TNFi	Respiratory survival	**aHR (95% CI) 0.4 (0.17 to 0.96**)		!	
Diabetes mellitus^29^	TNFi versus ABA	Diabetes treatment intensification¶	aHR (95% CI) 0.97 (0.82 to 1.15)			–
	RTX versus ABA	Diabetes treatment intensification¶	aHR (95% CI) 0.99 (0.79 to 1.23)			–
	TCZ versus ABA	Diabetes treatment intensification¶	aHR (95% CI) 0.94 (0.74 to 1.19)			–
	TOFA versus ABA	Diabetes treatment intensification¶	**aHR (95% CI) 0.67 (0.50 to 0.90**)			–
Obesity^42^	ETN versus ADA	DAS28-ESR remission	OR (95% CI) 1.01 (0.43 to 2.40)	+		
	INF versus ADA	DAS28-ESR remission	OR (95% CI) 0.77 (0.26 to 2.34)	+		
	ABA versus ADA	DAS28-ESR remission	OR (95% CI) 0.61 (0.16 to 2.25)	+		
CV risk factor^115^	TOFA versus TNFi	MACE	HR (95% CI) 1.33 (0.91 to 1.94)**			–
CV risk factor and age ≥65 years^115^	TOFA versus TNFi	MACE	HR (95% CI) 1.79 (0.99 to 3.26)			–
CV risk factor and current smoker^119^	TOFA versus TNFi	MACE	HR (95% CI) 1.20 (0.65 to 2.21)			–
CV risk factor and past smoker^119^	TOFA versus TNFi	MACE	HR (95% CI) 2.17 (0.95 to 4.96)			–
CV risk factor^127^	TOFA versus TNFi	CV outcome††	wHR (95% CI) 1.24 (0.90 to 1.69)			–
CV risk factor^114^	UPA versus ADA	MACE	HR (95% CI) 0.63 (0.13 to 3.07)			–
CV risk factor^116^	JAKi versus TNFi	MACE	aHR (95% CI) 1.03 (0.62 to 1.71)		!	
CV risk factor^117^	JAKi versus TNFi	MACE	HR (95% CI) 0.77 (0.45 to 1.34)			–
CV risk factor^118^	JAKi versus TNFi	MACE	aIRR (95% CI) 0.57 (0.12 to 2.66)		!	
CV risk factor^115^	TOFA versus TNFi	Malignancy (excluding NMSC)	**HR (95% CI) 1.48 (1.04 to 2.09**)			–
CV risk factor and age ≥65 years^115^	TOFA versus TNFi	Malignancy (excluding NMSC)	**HR (95% CI) 1.70 (1.00 to 2.90**)			–
CV risk factor^114^	UPA versus ADA	Malignancy (excluding NMSC)	**HR (95% CI) 0.37 (0.14 to 0.97**)			–
History of ASCVD^24^	TOFA versus TNFi	MACE	HR (95% CI) 1.98 (0.95 to 4.14)			–
History of CVD^127^	TOFA versus TNFi	CV outcome††	wHR (95% CI) 1.27 (0.95 to 1.70)			–
History of CVD^117^	JAKi versus TNFi	MACE	HR (95% CI) 0.79 (0.41 to 1.52)			–
History of CVD^26^	JAKi versus TNFi	MACE	HR (95% CI) 1.29 (0.59 to 2.81)		!	
History of CVD^118^	JAKi versus TNFi	MACE	aIRR (95% CI) 1.06 (0.56 to 2.02)		!	
HBcAb+/HBsAg−^61^	RTX versus ETN	HBV reactivation‡‡	**aHR (95% CI) 14.4 (4.0 to 51.2**)			–
	ABA versus ETN	HBV reactivation‡‡	**aHR (95% CI) 11.4 (2.3 to 55.9**)			–
	ADA versus ETN	HBV reactivation‡‡	**aHR (95% CI) 3.6 (1.0 to 13.1**)			–
	TOFA versus ETN	HBV reactivation‡‡	**aHR (95% CI) 6.5 (1.1 to 38.3**)			–
	TCZ versus ETN	HBV reactivation‡‡	aHR (95% CI) 2.1 (0.2 to 19.5)			–
	GOLI versus ETN	HBV reactivation‡‡	aHR (95% CI) 1.9 (0.3 to 10.7)			–
HBcAb+/HBsAg−^57^	ABA versus TNFi	HBsAg RS	**aHR (95% CI) 15.39 (3.08 to 77.04**)			–
	RTX versus TNFi	HBsAg RS	**aHR (95% CI) 35.65 (8.16 to 155.76)**			–
CKD (eGFR <45 mL/min/1.73 m^2^)^52^	TNFi versus JAKi	Drug discontinuation due to toxic AEs	**aHR (95% CI) 0.23 (0.09 to 0.61)**			–
	IL-6Ri versus JAKi	Drug discontinuation due to toxic AEs	**aHR (95% CI) 0.34 (0.14 to 0.81)**			–
	ABA versus JAKi	Drug discontinuation due to toxic AEs	**aHR (95% CI) 0.36 (0.15 to 0.89)**			–
CKD (eGFR <30 mL/min/1.73 m^2^)^50^	IL-6Ri versus TNFi	Drug discontinuation due to infection	aHR (95% CI) 0.64 (0.07 to 5.78)		!	

Bolded values indicate statistically significant estimates.

* Breast cancer diagnosed within the past 2 years before treatment exposure was assessed.

† ABA, RTX or TCZ.

‡ New primary, local recurrence or metastasis.

§ Serious exacerbations defined as hospitalisations or emergency department visits.

¶ Insulin and non-insulin treatment intensification.

** Risks of MACE are higher with tofacitinib and did not meet non-inferiority criteria of the trial.

†† Hospitalisations for myocardial infarction or stroke.

‡‡ HBsAg positive or detectable HBV DNA.

ABA, abatacept; ADA, adalimumab; AE, adverse event; aHR, adjusted HR; aIRR, adjusted incidence rate ratio; ASCVD, atherosclerotic cardiovascular disease; bDMARD, biologic disease-modifying antirheumatic drug; CKD, chronic kidney disease; COPD, chronic obstructive pulmonary disease; csDMARD, conventional synthetic disease-modifying antirheumatic drug; CV, cardiovascular; CVD, cardiovascular disease; DAS28-ESR, Disease Activity Score in 28 joints using erythrocyte sedimentation rate; eGFR, estimated glomerular filtration rate; ETN, etanercept; GOLI, golimumab; H, high risk of bias; HBcAb, hepatitis B core antibody; HBsAg, hepatitis B surface antigen; HBV, hepatitis B virus; ILD, interstitial lung disease; IL-6Ri, interleukin-6 receptor inhibitor; JAKi, Janus kinase inhibitor; L, low risk of bias; M, moderate risk of bias; MACE, major adverse cardiovascular event; NMSC, non-melanoma skin cancer; RoB, risk of bias; RS, reverse seroconversion; RTX, rituximab; SC, some concerns; TCZ, tocilizumab; TNFi, tumour necrosis factor inhibitor; TOFA, tofacitinib; UPA, upadacitinib; wHR, weighted HR.

#### Malignancy and cancer

Seven observational studies were identified for patients with RA with a history of malignancy (four moderate, two high, one low RoB). Compared with tumour necrosis factor inhibitors (TNFi), JAKi and non-TNFi bDMARDs did not show a significantly different cancer risk in patients with RA with prior malignancy initiating b/tsDMARDs in a Spanish registry, after a mean follow-up of 21 months. Rituximab and JAKi had the lowest numerical cancer risks, and interleukin-6 receptor inhibitors (IL-6Ri) and abatacept had higher risks (adjusted IRRs (95% CI) 0.3 (0.1 to 1.3), 0.7 (0.2 to 2.7), 1.7 (0.6 to 4.3) and 1.9 (0.8 to 4.7), respectively), noting that mean follow-up was shortest for rituximab and JAKi and longest for IL-6Ri and abatacept.^72^ Among patients with RA with prior solid cancer from the Danish Rheumatology Quality Register (DANBIO), TNFi (HR 1.10, 95% CI 0.21 to 3.16) and rituximab (HR 0.94, 95% CI 0.32 to 2.11) were not associated with an increased risk of cancer recurrence compared with csDMARDs, including in the breast cancer subcohort; median follow-up was 2.8, 4.7 and 3.5 years for TNFi, rituximab and csDMARDs, respectively.^74^ In another study of patients with solid malignancies, treatment with TNFi or non-TNFi bDMARDs was associated with no significant difference in overall survival after cancer diagnosis compared with patients not exposed to bDMARDs (HR 0.67 (95% CI 0.31 to 1.44), 1.10 (95% CI 0.26 to 4.60), respectively), although a clinically meaningful effect cannot be ruled out for non-TNFi. Comparable results were observed in the breast cancer subgroup.^71^ Similar findings from US claims data indicated that TNFi use after early breast cancer diagnosis, or in elderly patients with early-stage colorectal, lung or prostate cancer, was not associated with impaired survival.^70 73^ Overall, no significant difference was observed in cancer recurrence risk between b/tsDMARDs in patients with RA with prior malignancy, although most of the available studies were in patients with a history of solid cancer and suggested better safety with TNFi, followed by rituximab.

#### Respiratory comorbidities

Twenty-eight observational studies (13 moderate, 12 high and three low RoB), one RCT (high RoB) and one pooled analysis of RCT data were included for patients with respiratory comorbidities.

##### RA-associated interstitial lung disease

Comparing b/tsDMARDs among patients with RA-ILD, treatment with TNFi was associated with more than a twofold higher mortality risk compared with rituximab (HR 2.33, 95% CI 1.05 to 5.13).^94^ A separate study reported no differences in mortality for individual b/tsDMARDs compared with TNFi, but the combined analysis of b/tsDMARDs versus TNFi demonstrated significantly lower risk (HR 0.56, 95% CI 0.33 to 0.97) in line with the prior study.^90^ However, emulated trials found no significant differences in all-cause mortality or respiratory-related hospitalisation between TNFi and non-TNFi initiators (HR 1.21, 95% CI 0.92 to 1.58),^99^ or among abatacept, tocilizumab and tofacitinib compared with rituximab.^100^ Compared with TNFi, JAKi were associated with higher risks of all-cause mortality (HR 1.46, 95% CI 1.14 to 1.87); however, this excess risk was limited to older individuals (≥65 years) and those with CV comorbidities.^101^ Infections were the most frequent cause of tofacitinib discontinuation.^93^ Regarding efficacy, treatment with JAKi and abatacept significantly improved Clinical Disease Activity Index (CDAI) and Health Assessment Questionnaire-Disability Index (HAQ-DI), without an increase in pulmonary complications.^82 85^ JAKi reduced disease activity in patients with RA-ILD with ≥2 prior b/tsDMARD failure.^103^ Abatacept effectively reduced disease activity, with consistent efficacy across ILD subtypes and administration routes.^79 81 87 98^ Monotherapy and combination regimens with methotrexate or other csDMARDs yielded similar outcomes, while few cases discontinued abatacept due to ILD progression.^80 81^ The use of methotrexate was associated with a significantly lower risk of mortality (OR 0.28, 95% CI 0.09 to 0.88).^95 96^ Overall, rituximab appeared to have a lower mortality risk in patients with RA-ILD; abatacept was effective and safe and JAKi improved disease activity, but requires caution in elderly patients and those with CV comorbidities.

##### Other pulmonary comorbidities

Across studies in patients with RA with other pulmonary comorbidities, abatacept showed similar rates of chronic obstructive pulmonary disease (COPD) and asthma exacerbations as TNFi,^76^ while rituximab had fewer discontinuations and better respiratory survival in patients with bronchiectasis.^75^

### Metabolic syndrome, including obesity and diabetes mellitus

Seven observational studies (four moderate, two high and one low RoB), three pooled analyses of RCT data and one post hoc analysis of an RCT were included for obesity. No difference was shown between abatacept and adalimumab in reducing disease activity within 12 months among patients with obesity initiating their first bDMARD in a study using the Swiss Clinical Quality Management in Rheumatic Diseases (SCQM) registry.^42^ Although data from the German Rheumatoid Arthritis: oBservation of BIologic Therapy (RABBIT) registry also showed no negative impact of obesity on the effectiveness of abatacept or rituximab, obesity was associated with reduced improvement in Disease Activity Score assessing 28 joints based on erythrocyte sedimentation rate (DAS28-ESR) among women treated with TNFi (−0.22, 95% CI −0.31 to −0.12) or tocilizumab (−0.22, 95% CI −0.42 to −0.03), and among men treated with tocilizumab (−0.41, 95% CI −0.74 to −0.07).^40^ Consistently, lower DAS28 remission rates at 12 months in patients with and without obesity treated with TNFi were shown,^41^ although a large retrospective cohort found no evidence that non-TNFi bDMARDs were superior to TNFi in patients with obesity initiating their second or third bDMARD.^33^ A pooled analysis of five RCTs found obesity to be associated with a lower likelihood of remission in patients treated with tocilizumab with or without csDMARDs (CDAI HR 0.79, 95% CI 0.69 to 0.91),^39^ with another retrospective cohort similarly showing reduced CDAI-LDA achievement in patients with and without obesity at 6 and 12 months (OR 0.70 and 0.75, respectively).^34^ Rituximab treatment led to consistent improvements in disease activity, pain and fatigue over 12 months in patients with obesity.^37^ Pooled analyses of tofacitinib demonstrated efficacy over placebo in patients with RA and obesity for American College of Rheumatology (ACR) responses and CDAI-LDA achievement at both 3 and 6 months.^36^ For metabolic syndrome, an independent association with reduced treatment response at 6 months across both TNFi (OR 0.65, 95% CI 0.49 to 0.87) and non-TNFi bDMARDs was found (OR 0.76, 95% CI 0.55 to 1.04; moderate RoB).^43^ In post hoc analyses of phase III trials in patients with RA and diabetes, sarilumab demonstrated greater reductions in haemoglobin A1c than placebo or adalimumab with similar efficacy outcomes irrespective of diabetes status, and showed a safety profile consistent with the overall trial populations.^28^ Another study showed that treatment with TNFi was associated with increased insulin sensitivity compared with patients who did not receive biologic therapy (high RoB).^30^

### CV risk factors

Patients with CV risk factors represent a population for whom the use of JAKi should be exercised with caution, following the findings of the Oral Rheumatoid Arthritis Trial (ORAL) Surveillance.^115^ In total, one safety endpoint trial (high RoB), six post hoc analyses, three pooled analyses of RCTs and eight observational studies (six high, two moderate RoB) were identified. In ORAL Surveillance, tofacitinib was not non-inferior to TNFi for major adverse CV events (MACE; HR 1.33, 95% CI 0.91 to 1.94) and malignancies excluding non-melanoma skin cancer (HR 1.48; 95% CI 1.04 to 2.09). Post hoc analyses showed higher MACE risk in patients with a history of atherosclerotic cardiovascular disease (ASCVD) (HR 1.98; 95% CI 0.95 to 4.14), older age (≥65 years; HR 1.79; 95% CI 0.99 to 3.26) or smoking history (HR 2.17; 95% CI 0.95 to 4.96).^24 119^ The Safety of TofAcitinib in Routine care patients with Rheumatoid Arthritis (STAR-RA) study confirmed a higher, although non-significant, CV risk with tofacitinib versus TNFi (weighted HR 1.24; 95% CI 0.90 to 1.69). Elevated risk of CV outcomes was also observed in patients with a history of CVD (pooled weighted HR 1.27; 95% CI 0.95 to 1.70).^127^ However, results from another emulated trial, using data from the JAK-pot collaboration, showed a similar risk of MACE between JAKi and TNFi, overall as well as in patients with a history of CVD.^118^ Real-world data from a Canadian cohort showed lower MACE incidence rates with tofacitinib (1.6 (95% CI 0.6 to 3.3)/100 patient-years) compared with ORAL Surveillance, but the risk remained higher among patients with CV risk factors, while tofacitinib effectiveness was comparable between CV risk groups.^124^ For upadacitinib, a post hoc benefit-risk analysis of the SELECT-COMPARE study showed similar rates of adverse events (AEs) of special interest to adalimumab, except for higher rates of herpes zoster in both low and high CV risk groups, while maintaining superior clinical and functional efficacy.^122^ Although not powered for safety comparisons, results were consistent with previous integrated safety analyses of upadacitinib trials.^114^ For filgotinib, MACE rates were numerically higher in patients aged ≥65 years or with ≥1 CV risk factor. No dose-dependent effect was observed.^123^ Overall, the risk of MACE with JAKi appears more pronounced in high-risk RA populations (elderly, smokers, prior ASCVD).

### Patients with RA with ≥2 prior b/tsDMARD failures and active disease

#### Efficacy outcomes

Twelve studies were included for the efficacy of b/tsDMARDs in patients with ≥2 prior b/tsDMARDs (10 RCTs, a post hoc analysis and a pooled analysis of RCT data). Of these, eight were carried over from the prior SLR^128–135^ and four studies were newly identified, including two phase III placebo-controlled RCTs (contRAst 2 and contRAst 3),^136 137^ one integrated analysis of RCT data^138^ and one head-to-head phase III RCT (SELECT-CHOICE).^139^ In all RCTs, efficacy outcomes were derived from subgroup analyses. Three studies were judged to have some concerns, while the remainder were assessed as having a high RoB. The main study characteristics and efficacy outcomes are summarised in table 3.

**Table 3:** Studies reporting efficacy outcomes in patients with active RA with ≥2 prior b/tsDMARDs

Study ID	Study type	Treatment	N	N of prior b/ts DMARDs	ACR20 WK 12	ACR20 WK 24	Other outcomes/effect estimates	RoB
Fleischmann *et al*^136^; contRAst 2	RCT (subgroup analysis)	OTI 90 mg	40	≥2 bDMARDs	36.2%	59.6%	OR (95% CI) ACR20 (OTI 90 vs PBO at WK 12): 1.69 (0.44 to 6.46)	Some concerns
							OR (95% CI) ACR20 (OTI 90 vs TOFA at WK 24): 0.53 (0.15 to 1.84)	
		OTI 150 mg	40	≥2 bDMARDs	34.6%	49.7%	OR (95% CI) ACR20 (OTI 150 vs PBO at WK 12): 1.59 (0.42 to 6.04)	
							OR (95% CI) ACR20 (OTI 150 vs TOFA at WK 24): 0.35 (0.10 to 1.21)	
		TOFA 5 mg	20	≥2 bDMARDs	NA	72.2%		
		PBO	19	≥2 bDMARDs	24.1%	NA		
Taylor *et al*^137^; contRAst 3	RCT (subgroup analysis)	OTI 90 mg	27	≥2 bDMARDs	46.9%		OR (95% CI) ACR20 (OTI 90 vs PBO at WK 12): 1.01 (0.26 to 4.00)	Some concerns
		OTI 150 mg	29	≥2 bDMARDs	54.8%		OR (95% CI) ACR20 (OTI 150 vs PBO at WK 12): 1.40 (0.35 to 5.52)	
		PBO	14	≥2 bDMARDs	47.6%			
Tesser *et al*^138^	Pooled RCT data analysis	TOFA 5 mg	101	≥2 bDMARDs	40.6%		DAS28 <2.6: 6.8%; ACR50: 19.8%; ACR70: 7.9%; ΔHAQ-DI: −0.4 (WK 12)	High
		TOFA 10 mg	90	≥2 bDMARDs	52.2%		DAS28 <2.6: 6.0%; ACR50: 27.8%; ACR70: 10.0%; ΔHAQ-DI: −0.4 (WK 12)	
		PBO	69	≥2 bDMARDs	18.8%		DAS28 <2.6: 3.2%; ACR50: 10.1%; ACR70: 4.4%; ΔHAQ-DI: −0.1 (WK 12)	
Rubbert-Roth *et al*^139^; SELECT-CHOICE	RCT (subgroup analysis)	UPA 15 mg	43	≥3 bDMARDs of same MoA or ≥2 of multiple MoAs			Mean difference (95% CI) in ΔDAS28-CRP at WK 12 UPA versus ABA: −0.61 (−1.07 to −0.15)	Some concerns
		ABA	47					
Genovese *et al*^132^; FINCH 2	RCT (subgroup analysis)	FILGO 100 mg	33	2 bDMARDs	57.6%			High
		FILGO 200 mg	37	2 bDMARDs	70.3%			
		PBO	36	2 bDMARDs	33.3%			
		FILGO 100 mg	34	≥3 bDMARDs	58.8%			
		FILGO 200 mg	37	≥3 bDMARDs	70.3%			
		PBO	34	≥3 bDMARDs	17.6%			
Genovese *et al*^134^; SELECT-BEYOND	RCT (subgroup analysis)	UPA 15 mg	48	≥3 bDMARDs of same MoA or ≥2 of multiple MoAs	70.8%			High
		UPA 30 mg	54		50.0%			
		PBO	52		23.1%			
Genovese *et al*^130^; post hoc of RA-BEACON	Post hoc analysis of RCT (Genovese *et al*)^135^	BARI 2 mg	72	≥2 TNFi	43%		CDAI <10: 22%	High
		BARI 4 mg	70	≥2 TNFi	54%		CDAI <10: 20%	
		PBO	69	≥2 TNFi	25%		CDAI <10: 4%	
		BARI 2 mg	50	≥3 bDMARDs	38%		CDAI <10: 18%	
		BARI 4 mg	45	≥3 bDMARDs	53%		CDAI <10: 20%	
		PBO	47	≥3 bDMARDs	13%		CDAI <10: 2%	
Kremer *et al*^131^; BALANCE I	RCT (subgroup analysis)	UPA 3 mg	16	≥2 TNFi	63%			High
		UPA 6 mg	16	≥2 TNFi	56%			
		UPA 12 mg	15	≥2 TNFi	60%			
		UPA 18 mg	17	≥2 TNFi	71%			
		PBO	13	≥2 TNFi	39%			
Burmester *et al*^129^	RCT (subgroup analysis)	TOFA 5 mg	37	2 TNFi	37.8%			High
		TOFA 10 mg	30	2 TNFi	53.3%			
		PBO	37	2 TNFi	10.8%			
		TOFA 5 mg	11	≥3 TNFi	36.4%			
		TOFA 10 mg	12	≥3 TNFi	41.7%			
		PBO	9	≥3 TNFi	22.2%			
Smolen *et al*^133^; GO-AFTER	RCT (subgroup analysis)	GOLI*	71	2 TNFi	38%†		OR (95% CI) ACR20 (GOLI vs PBO): 3.2 (1.3 to 8.3)	High
		PBO	44	2 TNFi	16%†			
		GOLI*	22	3 TNFi	14%†		OR (95% CI) ACR20 (GOLI vs PBO): 0.9 (0.2 to 5.3)	
		PBO	21	3 TNFi	14%†			
Emery *et al*^128^; RADIATE	RCT (subgroup analysis)	TCZ 8 mg/kg	52	2 TNFi			ACR20: 50.0%; ACR50: 30.8%; ACR70: 15.4% (WK 24)	High
		TCZ 4 mg/kg	60	2 TNFi			ACR20: 28.3%; ACR50: 13.3%; ACR70: 3.3% (WK 24)	
		PBO	64	2 TNFi			ACR20: 10.9%; ACR50: 1.6%; ACR70: 0.0% (WK 24)	
		TCZ 8 mg/kg	26	3 TNFi			ACR20: 53.8%; ACR50: 19.2%; ACR70: 7.7% (WK 24)	
		TCZ 4 mg/kg	18	3 TNFi			ACR20: 22.2%; ACR50: 22.2%; ACR70: 0.0% (WK 24)	
		PBO	18	3 TNFi			ACR20: 5.6%; ACR50: 0.0%; ACR70: 0.0% (WK 24)	

* Combined 50 mg and 100 mg doses for golimumab.

† ACR20 responses assessed at week 14.

ABA, abatacept; ACR20/50/70, American College of Rheumatology 20%, 50% and 70% improvement criteria; BARI, baricitinib; CDAI, Clinical Disease Activity Index; DAS28, Disease Activity Score in 28 joints; FILGO, filgotinib; GO-AFTER, GOlimulab After Former anti-tumour necrosis factor α Therapy Evaluated in Rheumatoid arthritis; GOLI, golimumab; MoA, mechanism of action; N, number of participants; NA, not available; N of prior b/tsDMARD, number of prior biological or targeted synthetic disease-modifying antirheumatic drugs; OTI, otilimab; PBO, placebo; RA, rheumatoid arthritis; RADIATE, Research on Actemra Determining effIcacy after Anti-TNF failurEs; RCT, randomised controlled trial; RoB, risk of bias; TCZ, tocilizumab; TNFi, tumour necrosis factor inhibitor; TOFA, tofacitinib; UPA, upadacitinib; WK, week; ΔDAS28-CRP, change from baseline in Disease Activity Score in 28 joints based on C reactive protein; ΔHAQ-DI, change from baseline in Health Assessment Questionnaire Disability Index.

**Figure 2:**
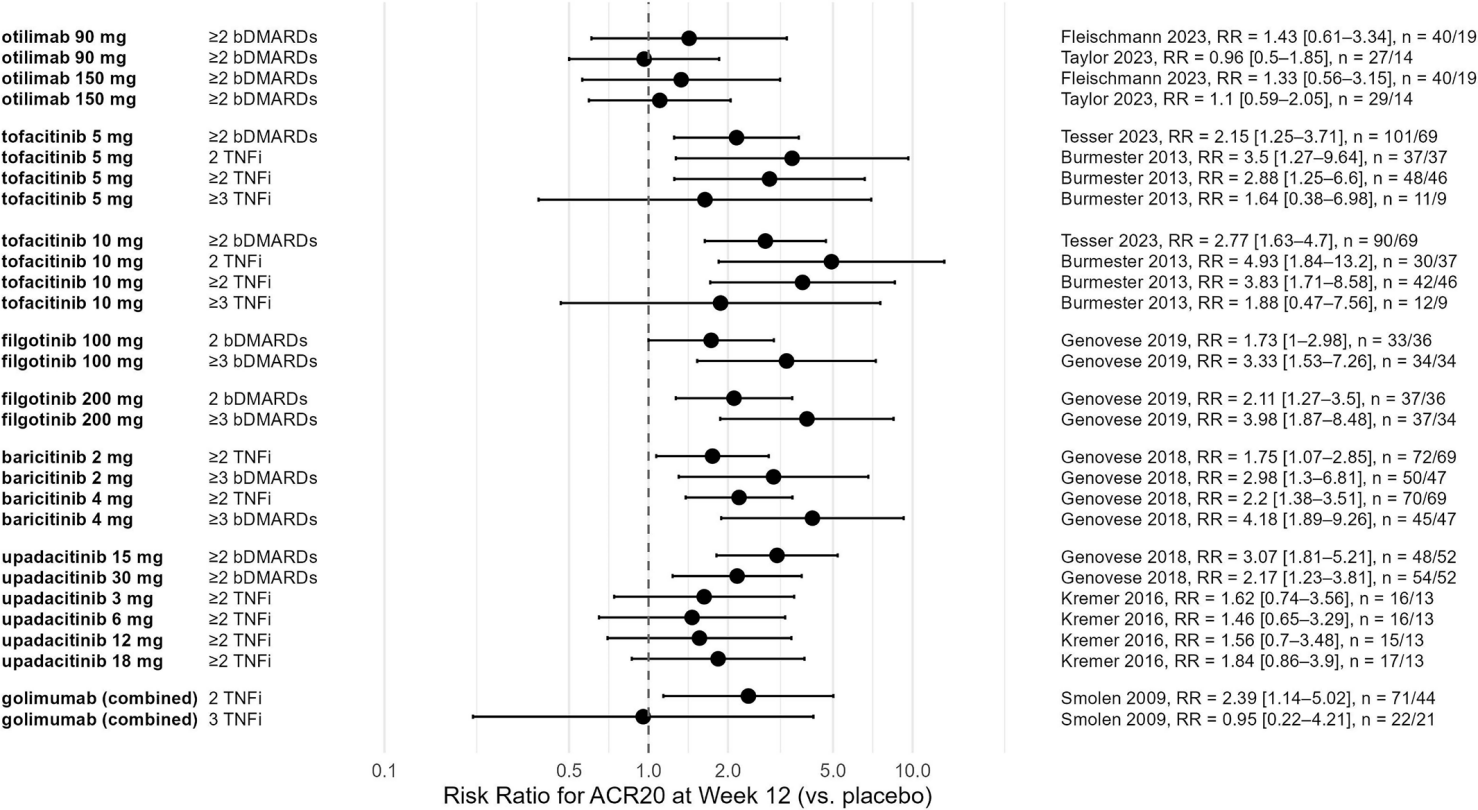
Risk ratios (RRs) for American College of Rheumatology 20% improvement (ACR20) response at week 12 in randomised controlled trials (RCTs) reporting on patients with ≥2 biologic or targeted synthetic disease-modifying antirheumatic drugs (b/tsDMARDs). Tesser *et al*^138^ includes data from Burmester *et al*^129^; both are shown due to providing different subgroup analyses and that their appearance does not represent double-counting. Smolen *et al*^133^ reports week 14 data. TNFi, tumour necrosis factor inhibitor.

**Figure 3:**
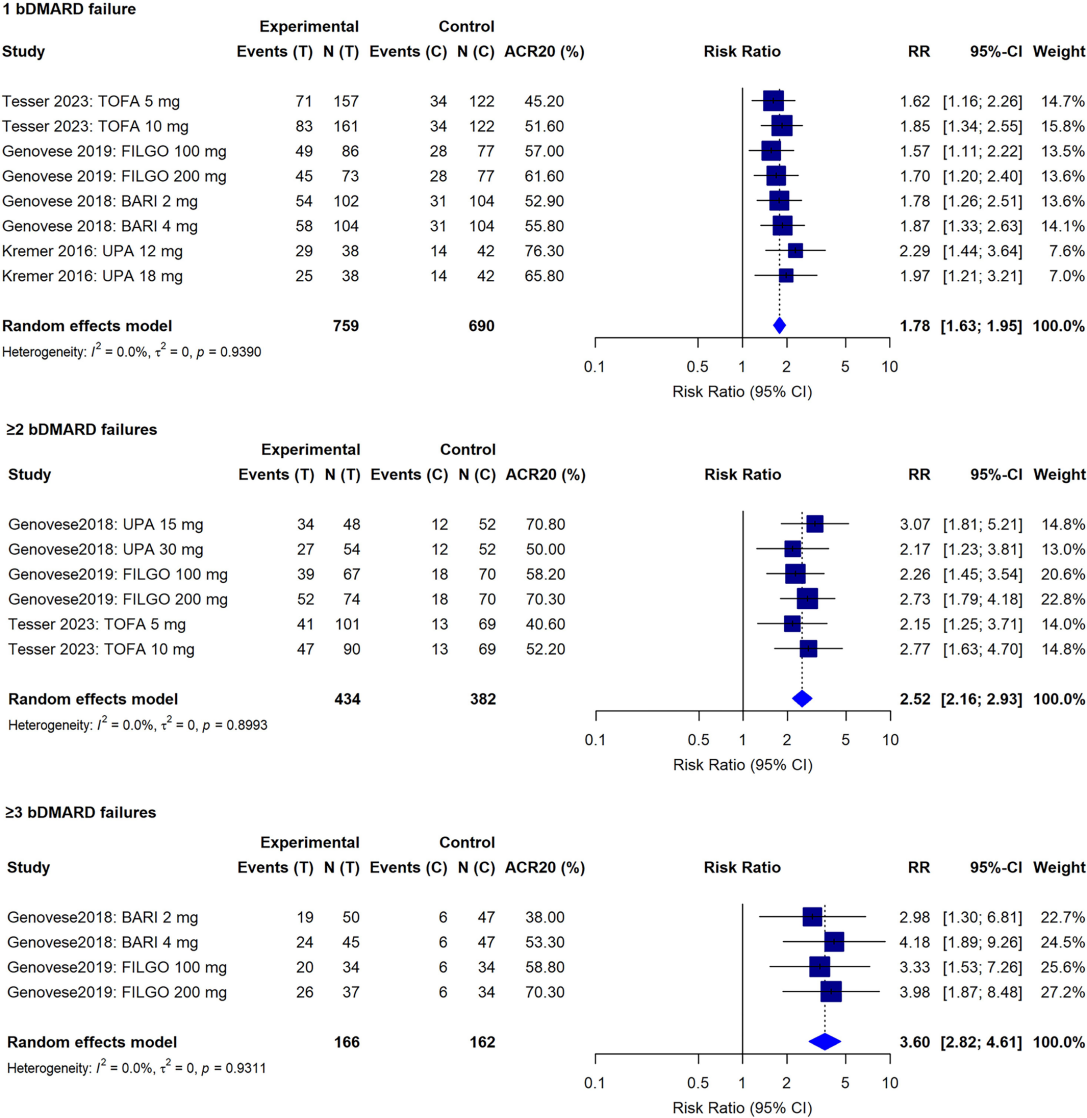
Forest plot summarising the risk ratios (RRs) for achieving an American College of Rheumatology 20% improvement (ACR20) response at week 12 with Janus kinase inhibitors (JAKi) versus placebo, stratified by the number of prior biologic disease-modifying antirheumatic drug (bDMARD) failures at baseline. Pooled estimates from random-effects meta-analysis are shown. BARI, baricitinib; FILGO, filgotinib; TOFA, tofacitinib; UPA, upadacitinib.

In contRAst 2, otilimab (90 or 150 mg administered subcutaneously once weekly) was compared with placebo and tofacitinib 5 mg twice daily, all on stable csDMARD background. In subgroup analyses of patients with ≥2 prior bDMARDs, otilimab showed no statistically significant benefit over placebo at week 12 (ORs 1.69, 95% CI 0.44 to 6.46 for 90 mg; 1.59, 0.42 to 6.04 for 150 mg). At week 24, ACR20 rates were 59.6% and 49.7% for otilimab 90 mg and 150 mg, respectively, vs 72.2% for tofacitinib, indicating numerically lower efficacy (ORs 0.53, 95% CI 0.15 to 1.84 for 90 mg; 0.35, 0.10 to 1.21 for 150 mg).^136^ A trend towards diminishing treatment effect was observed in subgroup analyses with increasing prior treatment failures, suggesting the observed inefficacy of otilimab versus placebo was beyond sample size effect. ContRAst 3, another phase III RCT, compared otilimab (90 or 150 mg administered subcutaneously once weekly), sarilumab (200 mg every 2 weeks) and placebo on stable csDMARDs in a refractory RA population. In subgroup analyses of patients with ≥2 prior bDMARDs, ACR20 responses at week 12 were 46.9% and 54.8% with otilimab 90 mg and 150 mg, respectively, compared with 47.6% for placebo, with no significant differences (otilimab 90 mg: OR 1.01, 95% CI 0.26 to 4.00; otilimab 150 mg: OR 1.40, 95% CI 0.35 to 5.52). Overall, otilimab failed to demonstrate efficacy over placebo. Sarilumab achieved significantly better outcomes to placebo and was superior to both otilimab doses in the overall population. Given the high proportion of patients with inadequate response to bDMARDs (bDMARD-IR) and JAKi (JAKi-IR) included, sarilumab appears to be an option for this patient population, although unfortunately no subgroup analyses were reported.^137^ Pooled analysis of seven phase II and six phase III tofacitinib trials evaluated patients with bDMARD-IR. In the subgroup analyses of patients with ≥2 failed bDMARDs, ACR20 responses at week 12 were 40.6% with tofacitinib 5 mg and 52.2% with 10 mg, compared with 18.8% for placebo. Higher-level responses followed a similar trend, although absolute rates were low (eg, ACR50: 19.8% and 27.8% vs 10.1%; ACR70: 7.9% and 10.0% vs 4.4%). DAS28-ESR remission (<2.6) was 6.8% and 6.0% for tofacitinib 5 mg and 10 mg vs 3.2% for placebo.^138^ In the SELECT-CHOICE trial, upadacitinib was compared with abatacept in patients with RA with bDMARD-IR on background csDMARDs. At baseline, approximately one-third of patients had failed ≥2 bDMARDs. In subgroup analyses stratified by ≥3 prior bDMARDs of the same MoA or ≥2 with different MoAs, a mean difference of −0.61 (95% CI −1.07 to −0.15) in Disease Activity Score in 28 joints based on C reactive protein at week 12 was shown, indicating consistent benefit in this population.^139^ Considering all available evidence from the current and previous SLR, in the majority of studies (n=9), efficacy was reported using ACR20 response at around week 12 versus placebo. The RR of achieving ACR20 versus placebo was calculated for each study and is graphically summarised in figure 2. Overall, JAKi (baricitinib, filgotinib, tofacitinib, upadacitinib) achieved higher ACR20 response rates than placebo, including in patients treated with ≥2 or ≥3 prior bDMARDs.^129 130 132^ Random-effects meta-analyses showed pooled RRs for ACR20 response with JAKi versus placebo of 2.52 (95% CI 2.16 to 2.93) in patients with ≥2 failures and 3.60 (95% CI 2.82 to 4.61) in those with ≥3 failures (figure 3). It was restricted to ACR20 at 12 weeks for JAKi as only they had studies with sufficient data on prior bDMARD exposure. Meta-regression analyses were then conducted to explore whether increasing the number of prior bDMARD exposures modified the effect of treatment. For this purpose, subgroup data for one prior bDMARD failure at baseline from the included studies were also extracted. When studies reported subgroup analyses for different numbers of prior treatment failures, mutually exclusive subgroups were used in the meta-regression analyses to avoid overlapping populations between failure categories. Meta-regression confirmed significantly higher RR for ACR20 in patients with ≥2 prior bDMARD failures compared with those with one prior failure (β=0.34, 95% CI 0.19 to 0.50, p=0.0004), and in those with ≥3 compared with ≥2 failures (β=0.45, 95% CI 0.11 to 0.79, p=0.016). This increase in RR was driven by decreased placebo response rates, while absolute ACR20 responses with JAKi were relatively unaffected by an increase in the number of previous bDMARDs (online supplemental tables 14, 15).

#### Safety outcomes

Nine observational studies were identified reporting safety outcomes in patients with ≥2 prior b/tsDMARDs (four moderate, three low and two high RoB).^140–148^

Three studies specifically included patients fulfilling the EULAR definition of D2T RA. Among them, none fully operationalised all three definitional components: criterion 1 was most consistently applied, criterion 2 was partially addressed in some and criterion 3 was not explicitly addressed in any (online supplemental table 16, detailing D2T RA component operationalisation across included studies).

In the FIRST registry and the Kansai Consortium for Well-being of Rheumatic Disease Patients (ANSWER) cohort, rates of serious or toxic AEs leading to treatment discontinuation were similar across b/tsDMARDs,^141 142^ although allergic reactions were more frequent with TNFi and concomitant glucocorticoid use (≥2.0 mg/day) increased the risk (HR 1.65, 95% CI 1.11 to 2.47).^142^ In patients with ≥2 prior b/tsDMARDs, JAKi were generally well tolerated, with AE-related discontinuation of 11.8% and 0% in patients with D2T (n=76) and non-D2T RA (n=25), respectively, but interpretation is limited by small sample size and the short 24-week follow-up period.^143^ Further details are provided in online supplemental table 13.

Other studies included RA populations with prior treatment failures without explicitly applying the EULAR D2T RA definition. In a separate analysis of the FIRST registry, infections were more frequent with JAKi compared with IL-6Ri, with herpes zoster occurring more often with JAKi. Serious infections and serious adverse events (SAEs), however, were rare and showed no between-group differences.^144^ A study using the British Society for Rheumatology Biologics Register for Rheumatoid Arthritis (BSRBR-RA) likewise found no link between serious infection risk and therapy line across bDMARDs.^145^ Higher malignancy risk with JAKi versus bDMARDs in patients with ≥3 prior b/tsDMARDs was reported in a study using the RABBIT registry (HR 1.98, 95% CI 1.01 to 3.90), particularly among those aged ≥60 years, whereas no excess risk was seen in patients aged <60 years. This pattern reflects higher baseline risk in older patients and in those with multiple lines of pretreatment.^146^

In patients with JAKi-IR with prior bDMARD failures, safety was comparable between patients cycling to another JAKi and those switching to a bDMARD. Switching to non-TNFi bDMARDs was associated with slightly lower AEs, although this difference was neither clinically nor statistically meaningful due to small number of cases.^147^ The JAK-pot collaboration study, however, found that patients who discontinued their first JAKi because of an AE were more likely to discontinue the second JAKi due to AE, compared with those switching to a bDMARD.^148^

### Efficacy of non-pharmacological interventions in b/tsDMARD-exposed patients with RA with low objective markers of disease activity

For patients with RA with predominantly non-inflammatory complaints, only two RCTs (high RoB) and one observational study (moderate RoB), which specifically enrolled EULAR-defined patients with D2T RA, were included.^149–151^ Most studies identified in the literature search on non-pharmacological interventions were conducted in unselected RA populations, often lacking information on prior b/tsDMARD use and disease activity status. In a propensity score-matched study, comparing the impact of orthopaedic surgical intervention (including wrist, finger, elbow, knee, toe, shoulder and ankle procedures) between EULAR-defined patients with D2T RA and non-D2T RA patients, both groups showed significant improvement in HAQ-DI, DAS28-ESR and patient general health (GH) at 12 months postsurgery. Patients with D2T RA demonstrated greater functional improvement with significant group-by-time interaction (p=0.048), indicating differential response patterns. HAQ-DI improvements remained independent of DAS28-ESR changes when adjusted as a covariate, suggesting benefits reflected restoration of joint function rather than reduction of systemic inflammation. However, the greater improvement in D2T RA may reflect their worse baseline functional status (HAQ-DI 1.05 vs 0.69, p=0.002), providing greater potential for functional gains. Of note, this was the only study attempting to operationalise criterion 2e of the EULAR D2T RA definition, defining it as ‘patient complaint attributed to RA in one or more joints’, deviating from EULAR intent by omitting well-controlled disease as a prerequisite and reducing ‘reduction in quality of life’ to merely having a joint complaint (online supplemental table 16).^151^ In another pilot RCT, patients with RA all previously treated with a bDMARD, completed 4 weeks of in-hospital rehabilitation (traditional therapy plus exergaming) and were randomised to home exergaming (n=20) versus habitual activity (n=20) for 8 weeks. Continuing videogame-based exercise did not lead to significant improvements in disease activity compared with habitual activity but led to maintained functional benefits versus deterioration with habitual activity.^150^

## Discussion

We conducted this SLR to provide an updated overview of the current evidence on management options for patients with D2T RA. Given that 5 years have passed since the publication of the EULAR definition of D2T RA, more stringent inclusion criteria were applied in this update to ensure that the identified evidence more accurately reflects this population. We identified 131 studies on patients with limited DMARD options, previous treatment failures and diminished quality of life despite low inflammatory activity. For patients with active RA but limited DMARD options, a higher number of studies were identified compared with the previous SLR, despite the exclusion of studies involving csDMARD-naïve patients and a minimum sample size requirement. This likely reflects growing research attention to RA populations with comorbidities or contraindications that limit DMARD use. Treatment restrictions due to AEs and comorbidities have been reported to be independently associated with D2T RA, with 94% of D2T patients affected by AEs and 69% having comorbidities, compared with 57% and 37% of those without D2T disease, respectively.^152^ Observational studies indicated no significant difference in cancer recurrence or mortality between b/tsDMARDs in patients with RA with prior malignancy, with TNFi showing consistent safety across those with a history of solid malignancies.^70–74^ The EULAR points to consider in patients with inflammatory arthritis and a history of cancer has comprehensively addressed the initiation of targeted therapies in this context.^153^ In patients with RA-ILD, rituximab showed favourable safety.^94^ JAKi and abatacept improved disease activity without exacerbating respiratory complications, but JAKi require caution in elderly or CV-compromised patients.^82 85 101^ Obesity negatively impacted treatment efficacy of most bDMARDs, whereas rituximab, abatacept and tofacitinib mostly seemed to retain efficacy.^34 36 37 39 40^ For patients with diabetes mellitus, limited available evidence suggested TNFi and sarilumab as better options.^28 30^ It remains uncertain whether comorbidities function as a predisposing factor for D2T RA, emerge sequentially from periods of poorly controlled inflammation or represent a bidirectional relationship where both processes reinforce one another. Future iterations of the D2T RA definition are challenged to acknowledge the role of individual comorbidities, whether as drivers, mediators, confounders or consequences. Possible updates of the EULAR points to consider may enable more directional classification and targeted management of individual comorbidities.

For patients with at least two prior b/tsDMARDs and active disease, otilimab exhibited no advantage over placebo.^136 137^ Meta-regression showed JAKi maintain efficacy despite increasing numbers of prior bDMARD treatments (1 to ≥3). However, this finding should be interpreted cautiously, as it may result from subgroup selection, small subgroup sizes and regression-related artefacts rather than true effect modification. A similar pattern was also observed in an observational study included for safety outcomes in this SLR, showing DAS28-ESR improvements at 24 weeks did not significantly differ between patients with one prior b/tsDMARD and patients with EULAR-defined D2T RA.^143^ The head-to-head comparison of upadacitinib versus abatacept, which showed superior efficacy of upadacitinib,^138^ and the numerically better ACR20 response with tofacitinib compared with otilimab further support a possible advantage of JAKi in D2T RA and may argue against the earlier hypothesis that their benefit is primarily due to starting a new MoA after failure of previous MoAs or their later introduction in treatment sequences. However, given the subgroup nature of these findings, interpretation should, of course, be made with caution. No new trials evaluated TNFi in this population, leaving the previous conclusion unchanged that non-TNFi bDMARDs and tsDMARDs appear more effective than switching to another TNFi after prior TNFi failure. So far, subgroup analyses of RCTs are mostly limited to ≥2 prior bDMARDs, or in the case of SELECT-CHOICE, ≥3 bDMARDs of the same MoA or ≥2 of multiple MoAs. In contRAst 3, despite inclusion of patients with JAKi-IR, subgroup analyses were still limited to ≥2 prior bDMARDs or ≥1 JAKi, without integrating tsDMARDs into the overall count or considering MoA change. As b/tsDMARDs both represent targeted therapies, there is reason to consider them specifically and future RCTs should prespecify subgroup definitions reflecting ≥2 b/tsDMARDs with different MoAs to enable better interpretation for the D2T RA population. For the question on the safety of treatments in patients with at least two prior b/tsDMARDs, three studies specifically included patients with D2T RA defined according to the EULAR definition, while the remaining evidence was indirect, which makes interpretation more difficult. Available observational studies indicated comparable rates of SAEs between bDMARDs and JAKi.^141–143^ However, glucocorticoids increased the risk of AEs leading to discontinuation in patients with D2T RA.^142^ The known increased occurrence of herpes zoster and malignancy with JAKi was likewise observed in these patients, particularly for malignancy in older individuals.^144 146^ Regarding non-pharmacological interventions in b/tsDMARD-exposed patients with RA with low objective markers of disease activity, only three studies could be identified. Orthopaedic surgical interventions to resolve functional impairments caused by joint damage, as a potential driver of D2T RA, should be considered in this subgroup.^151^ Most other studies on non-pharmacological interventions in the literature were conducted in RA populations lacking information on prior b/tsDMARD use or disease activity and were therefore excluded. Vagus nerve stimulation is another emerging non-pharmacological approach for patients with D2T RA. In a first-in-human study, it was safe, well-tolerated and reduced signs and symptoms of RA in individuals with multidrug-refractory disease, leading to the subsequent sham-controlled RESET-RA trial, which further confirmed the procedure’s safety.^154 155^ However, neither study met the inclusion criteria of PICO 3 due to small sample size and high disease activity of patients. Despite the growth of therapeutic options, evidence tailored to comorbidity treatment remains fragmented and predominantly observational. There is a lack of head-to-head comparative data and validated treatment algorithms for individuals with a D2T profile. Furthermore, the non-pharmacological management of residual pain, fatigue and diminished HRQoL in patients with low inflammatory activity is largely unexamined or poorly reported, representing a critical clinical and research gap. A major strength of this updated SLR is the use of more stringent inclusion criteria, ensuring that the included evidence reflects the intended population more precisely than in previous reviews. For example, a recent systematic review and network meta-analysis aimed to identify effective therapies for D2T RA, which included RCTs with populations that had failed at least one DMARD (csDMARD or b/tsDMARD). Therefore, its results may not be applicable to the D2T RA population.^156^ A limitation of this review is that treatment options restricted by socioeconomic factors were not addressed, as these non-clinical barriers were outside the scope of the current SLR, but warrant acknowledgement as important real-world contributors to the D2T RA state. Furthermore, efficacy data for patients with RA with previous treatment failure were derived from subgroup or post hoc analyses, mostly with a high RoB, and as efficacy data were considered more reliable when derived from RCTs, observational studies were not included in this SLR for these outcomes. Having experience with different MoAs was also not taken into account, as results were expected to be scarce. Moreover, meta-regression analyses were limited to JAKi due to insufficient data for other bDMARDs. Additionally, few studies could be included for non-pharmacological interventions. In conclusion, this updated SLR summarises current management options for patients with D2T RA. A clinically significant finding is the growing body of evidence supporting the safety profile of DMARDs in patients with multiple comorbidities. Given that co-existing conditions frequently restrict therapeutic options in patients with RA and D2T RA, these insights are highly relevant for clinical decision-making. Furthermore, JAKi showed maintained efficacy across patients with prior bDMARD failures; however, their use requires careful risk stratification, particularly in patients with high-risk CV or thromboembolic profiles, and uncertainty remains regarding their comparative effectiveness in D2T RA.

## Supplementary material

10.1136/rmdopen-2026-006860 online supplemental appendix 1

10.1136/rmdopen-2026-006860 online supplemental appendix 2

## Data Availability

All data relevant to the study are included in the article or uploaded as supplementary information.
