# Supplementary material for: Therapeutic approaches for difficult-to-treat rheumatoid arthritis: a systematic literature review of current evidence
Source: RMD Open. 2026 Aug 4;12(3):e006860. doi: 10.1136/rmdopen-2026-006860 (PMC13448634; doi:10.1136/rmdopen-2026-006860)
Supplement: Supplementary file 1 [file rmdopen-12-3-s001.pdf]

Therapeutic Approaches for Difficult-to-Treat Rheumatoid Arthritis: A Systematic Literature Review of Current Evidence

Supplementary Appendix 1

Supplementary Table 1. Summary of studies reporting on treatments' efficacy or safety in RA patients with limited options due to comorbidity, contraindication or drug-drug interactions.

Author, year	Study Design	Comorbidity or Limitation	Intervention/Exposure	n	Comparator	n	Time point	Outcome Description	N and/or % of responders in intervention group	N and and/or % of responders in comparator group	Mean outcome in intervention group (SD)	p-value (change from baseline Intervention)	Mean outcome in control group (SD)	p-value (change from baseline comparator)	Mean difference (95% CI)	p-value	Other outcomes/ effect estimate	Notes	Risk of Bias
**Osteoporosis and osteosarcopenia**																			
**<u>Studies regarding efficacy</u>**																			
Xie 2019	RCT	Secondary osteoporosis	Zoledronic Acid + MTX	18	MTX	18	6M	DAS28			4.89 (1.26)	p<0.001	4.27 (1.31)	p = 0.032		ns		Combined treatment was more effective than monotherapies, significantly improving VAS (vs. MTX at 6M and both ZOL/MTX at 12M), and ESR (vs. both ZOL/MTX at 6M and 12M).	High
								VAS(mm)			37.90 (14.04)	p<0.001	40.08 (15.88)	p = 0.048		p < 0.05			
								CRP(mg/L)			13.57 (15.89)	p = 0.031	15.39 (26.29)	p = 0.035		ns			
								ESR(mm/h)			30.61 (21.56)	p = 0.022	37.21 (25.47)	p = 0.038		p <0.05			
							12M	DAS28			4.17 (1.21)	p<0.001	4.12 (1.27)	p = 0.021		ns			
								VAS(mm)			29.23 (16.13)	p<0.001	38.72 (17.25)	p<0.001		p < 0.05			
								CRP(mg/L)			8.60 (12.27)	p = 0.029	11.02 (20.12)	p = 0.019		p <0.05			
								ESR(mm/h)			23.58 (17.87)	p = 0.008	36.24 (21.46)	p = 0.023		p <0.05			
			Zoledronic Acid Monotherapy	20	MTX	18	6M	DAS28			5.11 (1.33)	p = 0.036	4.27 (1.31)	p = 0.032		ns			
								VAS(mm)			43.02 (16.20)	p = 0.040	40.08 (15.88)	p = 0.048		ns			
								CRP(mg/L)			18.52 (29.51)	p = 0.077	15.39 (26.29)	p = 0.035		ns			
								ESR(mm/h)			40.25 (28.75)	p = 0.467	37.21 (25.47)	p = 0.038		ns			
							12M	DAS28			4.67 (1.28)	p = 0.019	4.12 (1.27)	p = 0.021		ns			
								VAS(mm)			37.02 (17.89)	p<0.001	38.72 (17.25)	p<0.001		ns			
								CRP(mg/L)			18.78 (23.38)	p = 0.079	11.02 (20.12)	p = 0.019		ns			
								ESR(mm/h)			38.67 (22.87)	p = 0.363	36.24 (21.46)	p = 0.023		ns			
Mori 2021	Observational cohort study	Osteoporosis in postmenopausal female	Denosumab 60 mg SC every 6 month	56	Oral bisphosphonates (Alendronate, Risedronate, or Minodronate)	50	12M	ΔDAS28-CRP			0.04 (0.53)		0.1 (0.74)			p = 0.9		Denosumab slowed the progression of mTSS and ERO compared to bisphosphonates. More patients in the denosumab group achieved structural remission (mTSS ≤ 0.5), meaning fewer had significant joint damage progression	Moderate
								ΔVAS			0.82 (15.7)		0.96 (18.1)			p = 0.82			
								ΔmHAQ			0.021 (0.31)		0.014 (0.23)			p = 0.72			
								ΔmTSS			**1.12 (2.31)**		**1.56 (2.15)**			**p = 0.01**			
								structural remission rate (the change of mTSS≤0.5)	58.9%	28%						**p<0.001**			
								ΔERO (modified sharp erosion score)			0.72 (1.58)		1.16 (1.47)			**p = 0.015**			
								ΔJSN (modified Sharp joint space narrowing score)			0.27 (0.84)		0.24 (0.55)			p = 0.92			
							24M	ΔDAS28-CRP			0.11 (0.72)		0.18 (0.96)			p = 0.84			
								ΔVAS			0.98 (17.6)		1.26 (20.5)			p = 0.87			
								ΔmHAQ			0.026 (0.32)		0.019 (0.27)			p = 0.78			
								ΔmTSS			1.73 (4.36)		2.52 (2.97)			**p = 0.003**			
								ΔERO (modified sharp erosion score)			1.07 (2.38)		1.56 (1.62)			**p = 0.013**			
								ΔJSN (modified Sharp joint space narrowing score)			0.61 (1.37)		0.56 (1.1)			p = 0.67			
Fujieda 2021	RCT	Osteoporosis induced by glucocorticoids	Risedronate 75mg	63	placebo	34	6M	ΔDAS28-ESR			1.82 (–0.79–11.6)	p=0.71	2.99 (11.2–1.18)	p=0.68	–1.16 (–18.6–16.2)	p=0.9		Risedronate did not significantly affect the change in DAS28-ESR compared to placebo (−1.16 [95% CI: −18.6 to 16.2], p = 0.90)	Some concerns
Yun 2023	RCT	postmenopausal women at risk for osteosarcopenia	Self-determination theory based physical activity programme using Tai Chi	31	control group (received routine care)	31	8W	DAS28-ESR			2.93 (0.13)		3.73 (0.13)			p < 0.001		The SDT-based physical activity programme led to significantly lower disease activity (DAS28-ESR) in the intervention group compared to the control group at both 8 weeks (hospital-based group intervention) (p < 0.001) and 16 weeks (home-based individual intervention) (p < 0.001), while HRQoL was significantly better only at 16 weeks (p = 0.004).	High
								EQ-5D-5L			0.78 (0.02)		0.76 (0.02)			p = 0.359			
							16W	DAS28-ESR			2.65 (0.17)		4.19 (0.17)			p < 0.001			
								EQ-5D-5L			0.82 (0.02)		0.74 (0.02)			p = 0.004			
**RA-ILD**																			
**<u>Studies regarding efficacy</u>**																			
Fernandez-Diaz 2020	Observational cohort study	RA-ILD	Abatacept (intravenously: 10 mg/kg every 4 weeks or subcutaneously: 125 mg weekly)	263	No control group		End of follow-up, median: 12M	DAS28 ESR			3.1 (1.3)	p <0.001						Abatacept significantly reduced disease activity (DAS28-ESR), CRP and ESR levels, and allowed for a reduction in corticosteroid dose, with these changes being statistically significant (P < 0.001).	High
								CRP(mg/L)			0.83 (0.41–1.67)	p <0.001							
								ESR(mm/h)			24.0 (10.0–42.0)	p <0.001							
								the median CS dose (mg/day)			5 (2.5–7.5)	p <0.001							
Cassone 2020	Observational cohort study	RA-ILD	Abatacept	44	No comparator		End of follow-up, median: 26.5 M	Remission or a Low disease activity	41 (93.2)										High

Fernández-Díaz 2021	Observational cohort study	RA-ILD	Abatacept + MTX	46	Abatacept monotherapy	111	End of follow-up, median: 12M	ΔDAS28-ESR			-1.2 (-1.8,-0.6)	p <0.001	-1.5 (-1.9, -1.0)	p <0.001		p = 0.58		Among Caucasian patients, Abatacept was similarly effective across all treatment groups in improving articular outcomes. Combination therapies showed significant corticosteroid-sparing effects	Moderate
								Corticosteroid dose reduction mg/day (median)			-2.7 (-4.6,-0.8)	p = 0.006	- 3.8 (-8.3,0.8)	p = 0.10		p = 0.67			
			Abatacept + csDMARDs other than MTX	106	Abatacept monotherapy	111	End of follow-up, median: 18M, 12M	ΔDAS28-ESR			-1.5(-1.8, -1.2)	p <0.001	-1.5 (-1.9, -1.0)	p <0.001		p = 0.92			
								Corticosteroid dose reduction mg/day (median)			-4.8(-6.3, -3.4)	p <0.001	- 3.8 (-8.3,0.8)	p = 0.10		p = 0.65			
Shu 2021	Observational cohort study	RA-ILD	Iguratimod	40	Control group, patients received the treatment of glucocorticoid cyclophosphamide	61	4W, 12W, 24W	ESR										The iguratimod group exhibited significantly lower ESR levels at weeks 4, 12, and 24, with p-values of p = 0.022, p = 0.004. p = 0.019, respectively. Exact numerical values were not provided.	Moderate
								DAS28-ESR										The Iguratimod group demonstrated a significant decrease in DAS28-ESR scores at 12W and 24W, with p-values of p = 0.034 and p = 0.003, respectively. Exact numerical values were not provided.	
								CRP										The iguratimod group exhibited significantly lower CRP levels at 4W, 12W, and 24W, with p-values of p = 0.036, p = 0.006. p < 0.001, respectively. Exact numerical values were not provided.	
Tardella 2022	Observational cohort study	RA-ILD	JAKi (tofacitinib at an oral dose of 5 mg BID or baricitinib at an oral dose of 4 mg daily)	31	Abatacept	44	18M	HAQ			0.71 (0.28)	p < 0.001	0.75 (0.29)	p < 0.001				Treatment with abatacept and JAKis significantly reduced CDAI and HAQ-DI in patients with RA-ILD. JAKis seem to reduce CDAI and HAQ more than abatacept (statistical significant not assessed)	Moderate
								CDAI			8.77 (7.24)	p < 0.001	10.11 (7.58)	p < 0.001					
Shen 2023	Observational cohort study	RA-ILD	Immunoglobulin (10g/d) given for five continuous days before methotrexate injection (10mg/week, once per week). patients were treated with 40mg/d prednisone for 4 weeks, which was then gradually decreased to 10 mg/d for maintenance.	40	Control group (prednisone together with the intravenous injection of methotrexate.	40	12M	ESR			7.4 (3.3)		14.1 (6.2)			p = 0.045			Moderate
Wang 2023	Observational cohort study	RA-ILD	Tofacitinib + iguratimod	24	csDMARDs	28	End of follow-up, mean: 5.9M	DAS28-CRP			3.8 (1.9)		3.5 (1.8)			p = 0.162		Disease activity did not change significantly between groups during the 6-month follow-up	High
								ESR (mm/h)			30.3 (18.2)		29.3 (23.1)			ns			
								CRP (mg/L)			20.7 (24.8)		23.7 (16.6)			ns			
			Iguratimod + csDMARDs	26	csDMARDs	28		DAS28-CRP			4.0 (1.7)		3.5 (1.8)			p = 0.426			
								ESR (mm/h)			34.3 (20.4)		29.3 (23.1)			ns			
								CRP (mg/L)			23.2 (35.2)		23.7 (16.6)			ns			
Atienza-Mateo 2024	Observational cohort study	RA-ILD	Abatacept in UIP (intravenously: 10 mg/kg every 4 weeks after an induction phase with two doses 2 weeks apart or subcutaneously: 125 mg weekly)	106	Abatacept in NSIP (intravenously: 10 mg/kg every 4 weeks after an induction phase with two doses 2 weeks apart or subcutaneously: 125 mg weekly)	84	End of follow-up: 60M	DAS28-ESR			2.9 (1.3)		3.1 (1.3)			p = 0.5		DAS28-ESR decreased, but the reduction was not statistically significant between groups. Also, the corticosteroid sparing effect was similar in both groups.	High
								Corticosteroid dose reduction mg/day (final median dose of prednisone)			5 [2.5-5]		5 [2.5-10]			p=0.08			
López-Maraver 2024	Observational cohort study	RA-ILD	SC abatacept	303	IV abatacept	94	24M	DAS28-ESR			3.02		3.4			p = 0.22		No significant differences between IV and SC abatacept.	Moderate
								Corticosteroid dose reduction mg/day (final median dose of prednisone)			5 [2.5–7.5]		5 [2.5–7.5]			p = 0.75			
Tsujii 2024	Nested case-control	RA-ILD	JAKi	21	Abatacept	21	12M	ΔDAS28-CRP			-2.03	p < 0.05	-1.74	p < 0.05		p = 0.057		JAKi is as safe and effective as ABT for patients with RA-ILD.	Low
								ΔHAQ			- 0.17	p < 0.05	- 0.54	p < 0.05		p = 0.551			
								Corticosteroid dose reduction mg/day			-3.3	p < 0.05	-3	p < 0.05		p = 0.488			
Triboulet 2025	Observational cohort study	RA-ILD	JAKi	42			Follow-up, median: 21M	ΔDAS28			-1.99	<0.001						In RA-ILD patients with extensive prior treatment failure (86% ≥2 and 79% ≥3 b/tsDMARDs), JAKi led to a marked reduction in disease activity. ILD remained overall stable, with only one acute exacerbation reported. Non-severe pulmonary infections occurred in 40% of patients, and the safety profile showed no new concerns.	High
								respiratory tract infection	16 (38.1%)										
								Acute ILD exacerbation	1 (2.38%)										
								Discontinuation due to intolerance	5 (11.9%)										
**Studies regarding safety**																			
Fernandez-Diaz 2020	Observational cohort study	RA-ILD	Abatacept (intravenously: 10 mg/kg every 4 weeks or subcutaneously: 125 mg weekly)	263	No control group		Follow-up, mean: 22.66 M	Serious infections	30 (11.4)									A total of 30 patients (11.4%) discontinued abatacept (ABA) due to adverse events. Despite this, ABA may be a safe treatment option for patients with RA-ILD	High
								ILD impairment required discontinuation	3 (1.14)										
								Cardiovascular events	3 (1.14)										
								Neoplasia	3 (1.14)										
								AE leading to treatment discontinuation	30 (11.4)										

Kang 2020	Observational cohort study	RA-ILD	Abatacept (Medicare)	420	TNFi (Medicare)	1,579	ABA:665 PY/ TNFi: 2258 PY	ILD exacerbations, IR per 100 PY	6.32	8.59							aIRR (95% CI): 0.68 (0.54-0.87)	Overall, there was no significant difference in the risk of polmunary exacerbations between abatacept and TNFi initiators.	Moderate
			Abatacept (MarketScan)	158	TNFi (MarketScan)	1138	ABA: 167 PY/ TNFi: 1441 PY	ILD exacerbations, IR per 100 PY	3.59	11.8							aIRR (95% CI): 0.27 (0.16-0.47)		
			Abatacept (combined cohorts)		TNFi (combined cohorts)			ILD exacerbations									aIRR (95% CI): 0.44 (0.18-1.09)		
Fernández-Díaz 2021	Observational cohort study	RA-ILD	Abatacept + MTX	46	Abatacept monotherapy	111	NR	AE leading to treatment discontinuation	6 (13%)	10 (9.0%)						p = 0.58 (between 3 groups)		Among Caucasian patients, Abatacept was similarly safe across all treatment groups.	Moderate
								Serious Infection	5(10.9%)	11(9.9%)						p = 0.74 (between 3 groups)			
								Cardiovascular Events	1 (2.2%)	1(0.9%)						p = 0.97 (between 3 groups)			
								Neoplasia	0	0									
			Abatacept + csDMARDs other than MTX	106	Abatacept monotherapy	111		AE leading to treatment discontinuation	14 (13.2%)	10 (9.0%)						p = 0.58 (between 3 groups)			
								Serious Infection	14(13.2%)	11(9.9%)						p = 0.74 (between 3 groups)			
								Cardiovascular Events	1 (0.9%)	1(0.9%)						p = 0.97 (between 3 groups)			
								Neoplasia	1 (0.9%)	0						p = 0.51 (between 3 groups)			
Shu 2021	Observational cohort study	RA-ILD	Iguratimod plus glucocorticoid/cyclophosphamide	40	Control group, patients received the treatment of glucocorticoid/cyclophosphamide	61		ALT increase	2 (5.0%)	5 (8.2%)						p = 0.827			Moderate
								WBC increase	4 (10.0%)	0 (0)						p = 0.046			
								PLT decrease	0 (0)	2 (3.3%)						p = 0.670			
Kelly 2021	Observational cohort study	RA-ILD	MTX	150	RA control	240	NR	All deaths Relative Risk									RR: 1.25 (0.86–1.81)	The analysis did not show any effect of csDMARDs (methotrexate, sulfasalazine, leflunomide, hydroxychloroquine) on all-cause or respiratory deaths in RA-ILD patients . Furthermore, no correlation was found between the dose or duration of methotrexate use and the extent or subtype of RA-ILD.	High
								Respiratory deaths Relative Risk (RR)									RR: 2.04 (1.14–3.65)		
			Sulphasalazine	145				All deaths Relative Risk (RR)									RR: 1.40 (0.98–2.00)		
								Respiratory deaths Relative Risk (RR)									RR: 2.90 (1.71–4.92)		
			Leflunomide	61				All deaths Relative Risk (RR)									RR: 1.23 (0.74–2.04)		
								Respiratory deaths Relative Risk (RR)									RR: 2.44 (1.22–4.89)		
			Hydroxychloroquine	55				All deaths Relative Risk (RR)									RR: 1.03 (0.57–1.85)		
								Respiratory deaths Relative Risk (RR)									RR: 2.24 (1.06–4.70)		
			Azathioprine	54				All-cause mortality_Relative Risk (RR)									RR: 1.42 (0.7–2.8)	Azathioprine and cyclophosphamide significantly increased respiratory mortality in RA-ILD patients (p = 0.02). Mycophenolate trended towards a reduction in all-cause mortality although numbers were small (p = 0.36).	
								Respiratory mortality_Relative Risk (RR)									**RR: 2.9 (1.2–6.9)**		
			Mycophenolate	42				All-cause mortality_Relative Risk (RR)									RR: 0.65 (0.2–2.0)		
								Respiratory mortality_Relative Risk (RR)									RR: 1.7 (0.5–6.0)		
			Cyclophosphamide	21				All-cause mortality_Relative Risk (RR)									RR: 1.65 (0.7–3.8)		
								Respiratory mortality_Relative Risk (RR)									RR: 3.6 (1.2–9.4)		
			TNFi	40				All-cause mortality_Relative Risk (RR)									RR: 2.09 (1.1-4.0)	Initial therapy with anti-TNF more than doubled the risk of dying compared with treatment with RTX.	
								Respiratory mortality_Relative Risk (RR)									RR: 2.50 (0.8-7.4)		
			Rituximab	37				All-cause mortality_Relative Risk (RR)									RR: 0.52 (0.2-2.0)		
								Respiratory mortality_Relative Risk (RR)									RR: 0.7 (0.1-5.3)		
Honne 2022	Observational cohort study	RA-ILD	bdmard	42	No comparator		NR	Pulmonary Events	4 (9.52)									7 patients developed severe pneumonia which required hospitalization: 3 in the bronchiectasis group (9.38%) and 4 in the ILD group (9.52%).	High
		Bronchiectasis		32					3 (9.38)										
Kalyoncu 2022	Observational cohort study	RA-ILD	tofacitinib	47	No comparator		Median follow-up: 15 M	AE leading to treatment discontinuation	20 (42.6)									In the RA-ILD group (n=47, median follow-up 15 mo [IQR: 7–32]), tofacitinib was discontinued in 20 patients (42.6%), with infections being the most common cause (6.3 per 100 patient-years). Concomitant methotrexate use with tofacitinib therapy was more common in the RA patients with ILD who discontinued tofacitinib therapy during the follow-up than in those who did not discontinue tofacitinib therapy (45.0% vs. 18.5%, p=0.05).	Moderate
								infection leading to discontinuation	5 (25.0)										
Kim 2022	Observational cohort study	RA-ILD	MTX	79			Mean follow-up duration: 4.3 years (SD 3.5)	Overall Survival, mean (95% CI), years			14.4 (13.3–15.4)		8.7 (7.2–10.2)			p < 0.001		The mean Overall Survival of MTX users was 14.4 years (95% CI = 13.3–15.4), which was significantly higher than the mean OS of nonusers (8.7 years, 95% CI = 7.2–10.2) [log-rank test, p < 0.001. The use of MTX was associated with a significantly lower risk of mortality (OR = 0.284, 95% CI = 0.091–0.880, p = 0.029).	Moderate
								Mortality – MTX users vs. non-users OR (95% CI); p									OR (95% CI) 0.284 (0.091–0.880); p=0.029		
Lee 2023	Observational cohort study	RA-ILD	TNFi	49	No comparator		Median duration of drug withdrawal: 26.9 M	Death	5 (10.2)									RA-ILD (n=159): 15 deaths (9.4%) – TNFi: 5 (33.3%), Abatacept: 7 (46.7%), Tocilizumab: 3 (20.0%), JAKi: 0 (0.0%). Median time to death in RA-ILD group: 5.0 years (IQR 3.1–6.6).	Moderate
			Abatacept	51					7 (13.7)										
			tocilizumab	45					3 (6.7)										
			JAKi	14					0										
Venerito 2023	Observational cohort study	RA-ILD	JAK inhibitors (JAKi): Baricitinib : 28 patients (65.12%), Tofacitinib: 9 patients (20.93%), Filgotinib: 3	43	No comparator		NR	Serious Infection	1 (2.32%)										High
								AE leading to treatment discontinuation	3 (6.98%)										

			patients (6.98%) Upadacitinib: 3 patients (6.98%)					Death	1 (2.32%)										
Shen 2023	Observational cohort study	RA-ILD	Immunoglobulin (10g/d) given for five continuous days before methotrexate injection (10mg/week, once per week). patients were treated with 40mg/d prednisone for 4 weeks, which was then gradually decreased to 10 mg/d for maintenance.	40	Control group (prednisone together with the intravenous injection of methotrexate.	40	Follow-up until 12M	Aminotransferase elevation	2 (5%)	1 (2.5%)									Moderate
								herpes zoster infection	2 (5%)	1 (2.5%)									
								Upper respiratory infection	2 (5%)	2 (5%)									
								Pulmonary infection	4 (10%)	4 (10%)									
								Thrombocytopenia,	1 (2.5%)	1 (2.5%)									
Tsujii 2024	Nested case-control	RA-ILD	JAKi	21	abatacept	21	24M	AE leading to treatment discontinuation	5 (23.8%)	4 (19.0%)									Low
								Infection leading to treatment discontinuation	2 (9.5%)	0									
								Pulmonary complications	2 (9.5%)	3 (14.3%)									
Rudi 2024	Observational cohort study	RA-ILD	csDMARDs	61	TNF inhibitors	114	Follow-up until 5Y	All cause mortality								p = 0.79	aHR (95% CI): 0.90 (0.42 to 1.94)	When non-TNFi bDMARDs and JAK inhibitors analyzed jointly, showed a lower mortality risk than TNFi (aHR = 0.56, 95% CI 0.33–0.97), though individual results lacked significance.	Low
			IL- 6 inhibitors	33	TNF inhibitors	114		All cause mortality								p = 0.29	aHR (95% CI): 0.62 (0.26 to 1.51)		
			Abatacept	60	TNF inhibitors	114		All cause mortality								p = 0.42	aHR (95% CI): 0.74 (0.36 to 1.53)		
			Rituximab	64	TNF inhibitors	114		All cause mortality								p = 0.10	aHR (95% CI): 0.56 (0.28 to 1.12)		
			JAKi	19	TNF inhibitors	114		All cause mortality								p = 0.64	aHR (95% CI): 0.78 (0.28 to 2.19)		
			IL6i, T- cell, B- cell and JAKi together		TNF inhibitors			All cause mortality								p = 0.04	aHR (95% CI): 0.56 (0.33 to 0.97)		
Otsuji 2024	Observational cohort study	RA-ILD	tocilizumab	55	No comparator	55	Follow-up up to 3Y	Respiratory Infection Leading to ILD Mortality	12 (32.4%)									Study suggests that immunosuppression from tocilizumab may have contributed to the deaths that were due to acute exacerbation of ILD induced by respiratory infection.	High
Kim 2024	Observational cohort study	RA-ILD	MTX	79	No comparator		NR	Infection	32 (40.5)			p=0.416						In the RA-ILD group, no statistically significant differences in the frequency of infections were observed between the individual drugs.	High
			Sulphasalazine	8					3 (37.5)			p=1.0							
			Leflunomide	9					2 (22.2)			p=0.487							
			etanercept	25					12 (48)			p=0.494							
			infliximab	3					3 (100)			p=0.558							
			adalimumab	14					5 (35.7)			p=0.862							
			golimumab	9					3 (33.3)			p=0.731							
			abatacept	55					18 (32.7)			p=0.779							
			tocilizumab	47					16 (34)			p=0.524							
			tofacitinib	10					3 (30)			p=0.744							
			baricitinib	6					3 (50)			p=0.674							
Shih 2025	Observational cohort study	RA-ILD	JAKi	812	TNFi	812	Follow-up up to 5Y	All-cause mortality									aHR (95% CI): 1.458(1.136–1.870)	JAK inhibitors were associated with higher risks of mortality, hospitalization, critical care use, and mechanical ventilation compared to TNF inhibitors. Subgroup analyses indicated that the heightened mortality risk was evident in older patients and in those with cardiovascular risk factors, but not observed in the 18–64 years age group or in patients without cardiovascular risk.	Moderate
								Hospital inpatient services									aHR (95% CI): 1.167(1.011–1.348)		
								Critical care services									aHR (95% CI): 1.854(1.414–2.431)		
								Mechanical ventilation									aHR (95% CI): 2.609(1.718–3.962)		
		RA-ILD with CV risk factor	JAKi		TNFi			All-cause mortality									aHR (95% CI): 1.636 (1.197–2.237)		
								Hospital inpatient services									aHR (95% CI): 1.183 (0.981–1.426)		
								Critical care services									aHR (95% CI): 1.929 (1.378–2.700)		
								Mechanical ventilation									aHR (95% CI): 2.539 (1.502–4.293)		
		RA-ILD without CV risk factor	JAKi		TNFi			All-cause mortality									aHR (95% CI): 0.936 (0.590–1.488)		
								Hospital inpatient services									aHR (95% CI): 0.874 (0.663–1.152)		
								Critical care services									aHR (95% CI): 1.134 (0.627–2.049)		
								Mechanical ventilation									aHR (95% CI): 1.355 (0.658–2.791)		
		RA-ILD and ≥65	JAKi		TNFi			All-cause mortality									aHR (95% CI):1.815 (1.316-2.503		
								Hospital inpatient services									aHR (95% CI):1.148 (0.939-1.404)		
								Critical care services									aHR (95% CI):1.698 (1.152-2.503)		
								Mechanical ventilation									aHR (95% CI): 3.076 (1.546-6.122)		
		RA-ILD and ≤ 65	JAKi		TNFi			All-cause mortality									aHR (95% CI):1.179 (0.760-1.829)		
								Hospital inpatient services									aHR (95% CI): 1.172 (0.936-1.468)		
								Critical care services									aHR (95% CI): 1.883 (1.249-2.840)		
								Mechanical ventilation									aHR (95% CI): 2.162 (1.227-3.807)		
Serrano-Combarro 2025	Observational cohort study	RA-ILD	Baricitinib	72			24M	ΔDAS28-ESR	-1.3									Baricitinib could be a useful and safe alternative in both pulmonary and joint disease in RA-ILD patients even in more refractory patients (median previous number of bDMARDs: 3)	High
								Infection	6										
								hypersensitivity pneumonitis	1										
								brain cancer	1										

								severe gastrointestinal intolerance	1										
								myocardial infarction	1										
								scleromalacia perforans	1										
Frideres 2025	Observational cohort study	RA-ILD	Abatacept	150	Rituximab	150	3Y	Composite of death or respiratory hospitalization									aHR (95% CI): 1.03(0.72,1.47)	Abatacept, tocilizumab, and tofacitinib vs rituximab showed no significant differences in survival or respiratory hospitalizations over 3 years in this propensity score–matched new-user cohort (target trial emulation design)	Moderate
								Respiratory hospitalization									aHR (95% CI): 0.99(0.65,1.51)		
								Death									aHR (95% CI): 1.02(0.63,1.65)		
								Respiratory death									aHR (95% CI): 1.15(0.38,3.44)		
			Tocilizumab	93	Rituximab	93	3Y	Composite of death or respiratory hospitalization									aHR (95% CI): 1.15(0.68,1.93)		
								Respiratory hospitalization									aHR (95% CI): 0.74(0.39,1.42)		
								Death									aHR (95% CI): 1.07(0.58,2.00)		
								Respiratory death									aHR (95% CI): 1.47(0.39,5.54)		
			Tofacitinib	74	Rituximab	74	3Y	Composite of death or respiratory hospitalization									aHR (95% CI): 0.89(0.54,1.46)		
								Respiratory hospitalization									aHR (95% CI): 0.89(0.44,1.78)		
								Death									aHR (95% CI): 0.82(0.45,1.50)		
								Respiratory death									aHR (95% CI): 0.65(0.19,2.20)		
England 2025	Observational cohort study	RA-ILD	non-TNFi	237	TNFi	237	3Y	Composite of death or respiratory hospitalisation									aHR (95% CI): 1·21 (0·92–1·58)		Moderate
								Respiratory hospitalization									aHR (95% CI): 1·27 (0·91–1·76)		
								Death									aHR (95% CI): 1·15 (0·83–1·60)		
							1Y	Composite of death or respiratory hospitalisation									aHR (95% CI): 1·38 (0·79–2·42)		
								Respiratory hospitalization									aHR (95% CI): 1·41 (0·93–2·13)		
								Death									aHR (95% CI): 1·53 (0·95–2·48)		
**Sjögren's Disease**																			
Tsuboi 2023	Observational cohort study (ROSE & ROSE II)	Sjögren's Disease	Abatacept IV	68	No comparator		52W	SDAI remission	19 (27.9%)									Abatacept ameliorated both glandular and extraglandular involvements. Serum RF levels also significantly reduced from baseline until 24W, however, it significantly re-increased to 221.6±618.8 IU/ml at 52weeks compared with baseline.	High
								SDAI (decreased)			9.9 (9.5)	p <0.05							
								Serum Igg (mg/dl) (decreased)			1529	p <0.05							
								Serum RF (IU/ml) (increased)			221.6 (618.8)	p <0.05							
								Any AEs	15 (22.1%)										
								AE leading to treatment discontinuation	3 (4.41%)										
**AA amyloidosis secondary to RA**																			
Nakamura 2012	Observational cohort study	AA amyloidosis secondary to RA	Cyclophosphamide (dose determined by level of 24h creatinine clearance)	62	Etanercept 25 mg SC twice weekly	24	Final observation	Survival										The Kaplan–Meier survival curve after treatment favored etanercept over cyclophosphamide (p = 0.025)	High
**Pulmonary Disorders**																			
Weinblatt 2006	RCT	COPD	Abatacept (<60 kg: 500mg; 60-100kg: 750 mg; >100kg: 1000 mg; at D1, D15, D29 and then Q4W)	37	Placebo	17		Serious adverse events	4 (11%)	0									High
								Adverse events involving the respiratory system	43.20%	23.50%									
								Infections	59.50%	58.80%									
Combe 2019	pooled analysis of RCT data	Pulmonary disorders (PD)	Baricitinib 4mg	77	Placebo	89	12W	ACR20 response (with PD)	66.20%	32.60%									High
		Terms for pulmonary disorders included allergic bronchitis, apnoea, asbestosis, aspirin exacerbated respiratory disease, asthma, asthma exercise-induced, atelectasis, bronchial hyper-reactivity, bronchiectasis, bronchiolitis, bronchitis chronic, bronchospasm, bullous lung disease, chronic obstructive pulmonary disease, cor pulmonale chronic, diffuse panbronchiolitis, dyspnoea, dyspnoea exertional, emphysema, idiopathic pulmonary disease, infantile asthma, interstitial lung disease, Langerhans cell histiocytosis, lung consolidation, lung cyst, obstructive airways disorder,		726		792		ACR20 response (without PD)	67.60%	39.90%									
				77		89		ACR50 response (with PD)	40.30%	12.40%									
				726		792		ACR50 response (without PD)	41.20%	14.60%									
				77		89		DAS28-CRP≤3.2 (with PD)	37.70%	16.90%									
				726		792		DAS28-CRP≤3.2 (without PD)	44.50%	16.50%									
			Baricitinib 4mg (with PD)	77	Baricitinib 4mg (without PD)	726	16W	Any TEAE, n (%), (EAIR)	59 (66.3%), (238)	435 (54.9%), (196)									
								SAE, n (%),(EAIR)	2 (2.2%), (8.1)	29 (3.7%), (13.1)									
								Discontinuation due to AEs, n (%), (EAIR)	4 (4.5%), (16.1)	20 (2.5%), (9.0)									
								Deaths, n (%), (EAIR)	0 (0.0%), (0.0)	1 (0.1%), (0.5)									
			Baricitinib 4mg (with PD)	373	Baricitinib 4mg (without PD)	3066	2Y median exposure	Any TEAE, n (%), (EAIR)	343 (92.0%), (45.7)	2567 (83.7%), (43.6)									
								SAE, n (%),(EAIR)	102 (27.3%), (13.6)	460 (15.0%), (7.8)									
								Discontinuation due to AEs, n (%), (EAIR)	35 (9.4%), (4.7)	284 (9.3%), (4.8)									
								Deaths, n (%), (EAIR)	4 (1.0%), (0.5)	9 (0.3%), (0.2)									

		pneumoconiosis, pneumonitis, pulmonary bulla and pulmonary fibrosis.																	
Suissa 2019	Observational cohort study	COPD	Abatacept	1807	other bDMARDs (adalimumab, anakinra, certolizumab pegol, etanercept, golimumab, infliximab, rituximab, tocilizumab and tofacitinib)	3547	NR	Combined respiratory endpoint (hospitalized COPD exacerbation, bronchitis and hospitalized pneumonia or influenza)									aHR (95% CI): 0.87 (0.681.12)		High
								Hospitalized COPD exacerbation									aHR (95% CI): 0.57 (0.33-0.99)		
Kang 2020	Observational cohort study	Chronic Obstructive Pulmonary Disease (COPD)	Abatacept (Medicare)	862	TNFi (Medicare)	3693	ABA: 1122 PY/ TNFi: 5272 PY	COPD exacerbations, IR per 100 PY	20.68	22.72							aIRR (95% CI): 0.93 (0.80-1.08)		Moderate
			Abatacept (MarketScan)	303	TNFi (MarketScan)	2303	ABA: 301 PY/ TNFi: 2808 PY	COPD exacerbations, IR per 100 PY	27.57	34.97							aIRR (95% CI): 0.85 (0.67-1.09)		
			Abatacept (combined cohorts)		TNFi (combined cohorts)			COPD exacerbations									aIRR (95% CI): 0.91 (0.80-1.03)		
Kang 2020	Observational cohort study	Asthma	Abatacept (Medicare)	478	TNFi (Medicare)	2108	ABA: 622 PY/ TNFi : 3322 PY	Asthma exacerbations, IIR per 100 PY	4.66	6.68							aIRR (95% CI):0.66 (0.51-0.84)		Moderate
			Abatacept (MarketScan)	270	TNFi (MarketScan)	2757	ABA: 309 PY/ TNFi: 4130 PY	Asthma exacerbations, IR per 100 PY	12.94	13.78							aIRR (95% CI):1.00 (0.78-1.30)		
			Abatacept (combined cohorts)		TNFi (combined cohorts)			Asthmaexacerbations									aIRR (95% CI):0.81 (0.54-1.22)		
Md Yusof 2020	Observational cohort study	Bronchiectasis	Rituximab	68	TNFi	46	6M	ΔDAS28-CRP (mean difference pre vs post-treatment)							-2.31 (–2.68 to –1.94)	p <0.001		Rituximab had better rates of discontinuation due to adverse lung outcomes than the matched TNFi cohort as well as better 5-year respiratory survival. Rituximab could be a better biologic options for patients with RA and bronchiectasis.	Moderate
								EULAR Good Response	26 (38.2%)										
								EULAR Moderate Response	35 (51.5%)										
							Follow-up up to 10Y	Death	9 (13.2%)	2 (4.3%)									
								Lung infection-related death	5 (7.4%)	2 (4.3%)									
								Lung exacerbation leading to discontinuation of medication	3 (4.4%)	13 (28.3%)									
								Treatment discontinuation due to respiratory causes	8 (11.8%)	15 (32.6%)									
								5-year respiratory survival								p = 0.041	aHR (95% CI): 0.4 (0.17, 0.96)		
**Diabetes Mellitus**																			
**Studies regarding efficacy**	-	-	-	-	-	-	-	-	-	-	-	-	-	-	-	-	-	-	-
Genovese 2020	Pooled analysis of RCT data	Diabetes	sarilumab 150mg (q2w)	50	placebo	50	6M	ACR20	20 (40)	17 (34)								The efficacy of sarilumab after 6 M of treatment, ACR20 was achieved by 40% (20 patients) receiving sarilumab 150 mg, and 58.6% (34 patients) in the sarilumab 200 mg group, compared to 34% (17 patients) in the placebo group. These post hoc analyses of three phase III clinical studies in patients with RA show that sarilumab as monotherapy or in combination with MTX/csDMARDs is associated with a greater reduction in HbA1c than adalimumab monotherapy or placebo + MTX/csDMARDs, particularly in patients with diabetes.	High
			sarilumab 200mg (q2w)	58				ACR20	34 (58.6)										
Avouac 2021	Cross-sectional	Diabetes	TNFi	21	non-biologics	NA		DAS28			2.38 (1.03)		3.28 (1.29)			p = 0.012		Mean DAS28 of TNFi users was lower compared to other biologics as well as patients not receiving biologics. Also, increased insulin sensitivity in TNFi users was observed compared with patients not receiving biologics.	High
Specker 2022	Observational cohort study	Diabetes	Tocilizumab	263	No comparator		Follow-up until 104W	ΔDAS28-ESR			-2.2 (1.8)	p = 0.82							High
Di Muzio 2024	Observational cohort study	Type 2 Diabetes	Tofacitinib	40	No comparator		3M	DAS-28-CRP<2.6	34.9%										High
							3M	DAS-28-CRP≤3.2	65.1%										
							6M	DAS-28-CRP<2.6	41.9%										
							6M	DAS-28-CRP≤3.2	76.8%										
**Studies regarding Safety**	-	-	-	-	-	-	-	-	-	-	-	-	-	-	-	-	-	-	-
Weinblatt 2006	RCT	Diabetes Mellitus	Abatacept (<60 kg: 500mg; 60-100kg: 750 mg; >100kg: 1000 mg; at D1, D15, D29 and then Q4W)	65	Placebo	31	1 Y	Serious adverse events	21.50%	12.90%									High
								Infections	50.80%	58.10%									
Chen 2020	Observational cohort study	Diabetes Mellitus	TNFi	5953	Abatacept	1785	NR	Herpes Zoster Infection IR (95% CI) per 1000 PY	17.5 (14.3, 21.4)	17.2 (11.5, 25.6)							aHR (95%CI): 1.48 (0.88, 2.49)		High
								Diabetes treatment intensification (insulin and non-insulin)									aHR (95%CI): 0.97(0.82,1.15)		
			Rituximab	888	Abatacept	1785		Herpes Zoster Infection IR (95% CI) per 1000 PY	33.3 (22.1, 50.1)	17.2 (11.5, 25.6)							aHR (95%CI): 1.82 (1.02, 3.24)		
								Diabetes treatment intensification (insulin and non-insulin)									aHR (95%CI): 0.99(0.79,1.23)		
			Tocilizumab	759	Abatacept	1785		Herpes Zoster Infection IR (95% CI) per 1000 PY	34.1 (21.8, 53.5)	17.2 (11.5, 25.6)							aHR (95%CI): 1.98 (1.06, 3.68)		
								Diabetes treatment intensification (insulin and non-insulin)									aHR (95%CI): 0.94(0.74,1.19)		
			Tofacitinib	634	Abatacept	1785		Herpes Zoster Infection IR (95% CI) per 1000 PY	36.8 (22.2, 61.0)	17.2 (11.5, 25.6)							aHR (95%CI): 2.16 (1.09, 4.28)		

								Diabetes treatment intensification (insulin and non-insulin)									**aHR (95%CI): 0.67(0.50,0.90)**		
			sarilumab 150mg (q2w)					treatment-emergent adverse event (TEAE)	21 (77.8)	20 (74.1)									
			sarilumab 200mg (q2w)						36 (97.3)										
			sarilumab 150mg (q2w)					Treatment-emergent serious adverse event	3 (11.1)	4 (14.8)									
			sarilumab 200mg (q2w)						5 (13.5)										
			sarilumab 150mg (q2w)					TEAE leading to death	0	1 (3.7)									
			sarilumab 200mg (q2w)						0										
			sarilumab 150mg (q2w)					TEAE leading to discontinuation	1 (3.7)	2 (7.4)									
			sarilumab 200mg (q2w)						5 (13.5)										
			sarilumab 150mg (q2w)					infection	12 (44.4)	12 (44.4)									
			sarilumab 200mg (q2w)	27	placebo	27	13 M		20 (54.1)										
			sarilumab 150mg (q2w)					serious infection	1 (3.7)	4 (14.8)									
			sarilumab 200mg (q2w)						1 (2.7)										
			sarilumab 150mg (q2w)					infection leading to discontinuation	0	1 (3.7)									
			sarilumab 200mg (q2w)						0										
			sarilumab 150mg (q2w)					tuberculosis	0	0									
			sarilumab 200mg (q2w)						0										
			sarilumab 150mg (q2w)					hypersensitivity	1 (3.7)	1 (3.7)									
Genovese 2020	subanalysis_RCT	Diabetes	sarilumab 200mg (q2w)						3 (8.1)									Sarilumab was generally well tolerated, and adverse events observed in patients with diabetes were consistent with the overall safety profile of sarilumab. Hypersensitivity reactions were reported exclusively in the sarilumab-treated group. No association was observed between sarilumab treatment and the occurrence of tuberculosis.	High
			sarilumab 150mg (q2w)					treatment-emergent adverse event (TEAE)	18 (78.3)	17 (73.9)									
			sarilumab 200mg (q2w)						13 (61.9)										
			sarilumab 150mg (q2w)					Treatment-emergent serious adverse event	2 (8.7)	1 (4.3)									
			sarilumab 200mg (q2w)						2 (9.5)										
			sarilumab 150mg (q2w)					TEAE leading to death	0	0									
			sarilumab 200mg (q2w)						0										
			sarilumab 150mg (q2w)					TEAE leading to discontinuation	3 (13.0)	1 (4.3)									
			sarilumab 200mg (q2w)						2 (9.5)										
			sarilumab 150mg (q2w)	23	placebo	23	6 M	infection	10 (43.5)	8 (34.8)									
			sarilumab 200mg (q2w)						4 (19.0)										
			sarilumab 150mg (q2w)					serious infection	1 (4.3)	0									
			sarilumab 200mg (q2w)						0										
			sarilumab 150mg (q2w)					infection leading to discontinuation	3 (13.0)	0									
			sarilumab 200mg (q2w)						0										
			sarilumab 150mg (q2w)					tuberculosis	0	0									
			sarilumab 200mg (q2w)						0										
			sarilumab 150mg (q2w)					hypersensitivity	3 (13.0)	2 (8.7)									

			sarilumab 200mg (q2w)						4 (19.0)										
**Obesity**																			
**Studies regarding efficacy**	-	-	-	-	-	-	-	-	-	-	-	-	-	-	-	-	-	-	-
Gremese 2013	Observational cohort study	Obesity	TNFi (obese)		TNFi (non-obese)		12M	DAS28 remission (Normal vs obese)	10 (15.2%)	184 (32.0%)							OR (95% CI): 2.43 (1.21–4.88)		Moderate
			etanercept (obese)		etanercept (non-obese)				27.60%	36.20%						p = 0.08			
			adalimumab (obese)		adalimumab (non-obese)				14.80%	30.10%						p = 0.44			
Abuhelwa 2020	Pooled analysis of RCT data	Obesity	Tocilizumab±csDMARDs	1256	csDMARDs	374	-	SDAI-remission _HR (95% CI) (normal BMI as reference)	0.84 [0.72 0.98]	0.67 [0.48 0.93]								Obesity was associated with a reduced chance of achieving remission in RA patients, independent of the type of DMARD therapy (tocilizumab or csDMARDs).	High
			Tocilizumab±csDMARDs	1267	csDMARDs	375		CDAI-remission_HR (95% CI) (normal BMI as reference)	0.79 [0.69 0.91]	0.69 [0.50 0.94]									
Pappas 2020	Observational cohort study	Obesity	Tocilizumab IV or SC	356	No comparator		6M	ΔCDAI			-7.38								Moderate
								CDAI LDA (≤10)	19.7%								OR (95% CI)(obese vs non-obese): 0.70 (0.48, 1.01)		
								ΔHAQ			-0.05								
							12M	ΔCDAI			-8.09								
								CDAI LDA (≤10)	27.5%								OR (95% CI)(obese vs non-obese): 0.75 (0.53, 1.06)		
								ΔHAQ			-0.04								
Schäfer 2020	Observational cohort study	Obesity, stratified by sex	TNFi (obese female patients)	NR	TNFi (non-obese female patients)	NR	6M	DAS28-ESR improvement							−0.22 (−0.31; −0.12)	**<0.0001**		No negative effect of obesity was observed on the effectiveness of rituximab or abatacept.	Moderate
								CDAI improvement							−1.37 (−2.39;−0.36)	**0.008**			
								SDAI improvement							−4.79 (−6.76;−2.82)	**<0.0001**			
			TNFi (obese male patients)		TNFi (non-obese male patients)			DAS28-ESR improvement							0.001 (−0.16; 0.16)	0.994			
								CDAI improvement							0.59 (−1.17; 2.34)	0.512			
								SDAI improvement							−0.30 (−3.80; 3.19)	0.865			
			Abatacept (obese female patients)		Abatacept (non-obese female patients)			DAS28-ESR improvement							−0.23 (−0.52; 0.06)	0.117			
								CDAI improvement							−2.18 (−4.81; 0.45)	0.105			
								SDAI improvement							−3.98 (−9.06; 1.11)	0.126			
			Abatacept (obese male patients)		Abatacept (non-obese male patients)			DAS28-ESR improvement							0.22 (−0.23; 0.68)	0.33			
								CDAI improvement							0.57 (−4.04; 5.17)	0.81			
								SDAI improvement							3.78 (−5.48; 13.04)	0.423			
			Rituximab (obese female patients)		Rituximab (non-obese female patients)			DAS28-ESR improvement							−0.13 (−0.42; 0.15)	0.367			
								CDAI improvement							−5.46 (−9.76;−1.15)	**0.013**			
								SDAI improvement							−9.02 (−17.19;−0.85)	**0.031**			
			Rituximab (obese male patients)		Rituximab (non-obese male patients)			DAS28-ESR improvement							0.18 (−0.26; 0.63)	0.419			
								CDAI improvement							0.52 (−5.19; 6.23)	0.859			
								SDAI improvement							4.29 (−6.62; 15.20)	0.441			
			Tocilizumab (obese female patients)		Tocilizumab (non-obese female patients)			DAS28-ESR improvement							−0.22 (−0.42;−0.03)	**0.026**			
								CDAI improvement							−3.14 (−5.29;−1.00)	**0.004**			
								SDAI improvement							−5.98 (−10.13;−1.84)	**0.005**			
			Tocilizumab (obese male patients)		Tocilizumab (non-obese male patients)			DAS28-ESR improvement							−0.41 (−0.74;−0.07)	**0.018**			
								CDAI improvement							−2.00 (−5.56; 1.56)	0.271			
								SDAI improvement							−1.47 (−8.24; 5.30)	0.67			
Baker 2022	Observational cohort study	Obesity	TNFis	1347	-	-	24W	CDAI LDA (≤10)									OR (95% CI)(obese vs non-obese): 0.88 (0.72,1.08)	In patients initiating bDMARDs as second or third-line therapy, there was no evidence that non-TNFi therapies were superior to TNFi therapies in obese patients	Moderate
			non-TNFi bDMARDs	1365				CDAI LDA (≤10)									OR (95% CI)(obese vs non-obese): 0.82 (0.67,1.01)		
Dikranian 2022	Pooled analysis of RCT data	Obesity	Tofacitinib 5mg BID	388	Placebo	186	3M	ACR20	52.4%	22.6%						p<0.05		Tofacitinib showed greater efficacy improvements versus placebo in both doses at at month 3 and 6 in obese patients.	High
								ACR50	28.3%	8.1%						p<0.05			
								ACR70	13.4%	2.7%						p<0.05			
								DAS28-ESR ≤3.2	16.9%	2.9%						p<0.05			
								CDAI LDA (≤10)	29.1%	8.6%						p<0.05			
							6M	ACR20	54.6%	32.3%						p<0.05			
								ACR50	35.6%	13.4%						p<0.05			
								ACR70	17.9%	7.0%						p<0.05			
								DAS28-ESR ≤3.2	19.4%	8.1%						p<0.05			

								CDAI LDA (≤10)	34.0%	16.1%						p<0.05			
			Tofacitinib 10mg BID	443	placebo	186	3M	ACR20	59.2%	22.6%						p<0.05			
								ACR50	33.9%	8.1%						p<0.05			
								ACR70	16.6%	2.7%						p<0.05			
								DAS28-ESR ≤3.2	18.7%	2.9%						p<0.05			
								CDAI LDA (≤10)	34.4%	8.6%						p<0.05			
							6M	ACR20	59.0%	32.3%						p<0.05			
								ACR50	39.4%	13.4%						p<0.05			
								ACR70	20.7%	7.0%						p<0.05			
								DAS28-ESR ≤3.2	24.0%	8.1%						p<0.05			
								CDAI LDA (≤10)	42.0%	16.1%						p<0.05			
Novella-Navarro 2022	Observational cohort study	Obesity	Tocilizumab	18	TNFis	34		CDAI LDA (≤10)	8 (44.4)	16 (47.0)									High
Balsa 2023	Pooled analysis of RCT data	Obesity																Filgotinib 200 mg, with or without methotrexate, demonstrated greater efficacy than the control treatments across all BMI subgroups including obese patients in ACR20, ACR50, and ACR70 response rates, DAS28-CRP low disease activity and remission, CDAI low disease activity and remission, and HAQ-DI. (No numerical value presented)	High
	FINCH 1		Filgotinib 200	124	Filgotinib 100	115	12W	ACR 20/ ACR50/ ACR 70 response, DAS-28-CRP≤3.2, DAS-28-CRP<2.6, CDAI low disease activity, CDAI remission, HAQ-DI (mean change from baseline)											
					Adalimumab	80													
					placebo	123													
	FINCH 2		Filgotinib 200	66	Filgotinib 100	69													
					placebo	64													
KARATAŞ 2023	Observational cohort study	Obesity	Rituximab 1000 mg (on days 0 and 15)	59	No comparator		6 M	ΔESR (mm/h)			–17.5 (–46 to 11)							In obese patients, rituximab treatment led to consistent improvements in disease activity, pain, fatigue, and inflammation over 6 and 12 months	High
								ΔCRP (mg/dL)			–10 (–67 to 4)								
								ΔDAS28			–1.6 (–4.4 to 0.4)								
								ΔHAQ			–0.25 (–1.87 to 0.98)								
								Patient pain ΔVAS			–30 (–59 to 2)								
								Patient fatigue ΔVAS			–20 (–58 to 1)								
								Patient global ΔVAS			–20 (–55–0)								
								Physician global ΔVAS			–43 (–63 to 4)								
							12M	ΔESR (mm/h)			–2 (–48 to 11)								
								ΔCRP (mg/dL)			–3 (–38 to 19)								
								ΔDAS28			–1.95 (–5.4 to 0.8)								
								ΔHAQ			–0.31 (–1.25 to 0)								
								Patient pain ΔVAS			–27.5 (–75 to 30)								
								Patient fatigue ΔVAS			–32.5 (–75 to 16)								
								Patient global ΔVAS			–20 (–75 to 30)								
								Physician global ΔVAS			–47.5 (–85 to 0)								
Baker 2024	Post-hoc analysis RCT (RACAT)	Obesity	Etanercept + MTX	60	Sulfasalazine + hydroxychloroquine + MTX	75	24W	ΔDAS28			-1.91 (0.16)		-1.90 (0.14)			p = 0.98		Triple therapy and Etanercept had comparable efficacy in reducing disease activity and achieving ACR responses in obese patients at 24 weeks.	High
								ACR20	38%	45%						p = 0.32			
								ACR50	20%	22%						p = 0.58			
								ACR70	5%	7%						p = 0.61			
Vallejo-Yagüe 2024	Observational cohort study	Obesity	Etanercept	150	Adalimumab	178	Follow-up until 12M	DAS-28-ESR remission (<2.6)	48 (32.0%)	57 (32.0%)							OR (95% CI): 1.01 (0.43–2.40)	Abatacept did not increase the likelihood of remission or LDA compared with adalimumab among obese patients with RA. However, obese infliximab users had significantly less reduction in ESR and CRP compared to adalimumab users.	Low
								DAS-28-ESR LDA (≤3.2)	73 (48.7%)	87 (48.9%)							OR (95% CI): 0.77 (0.33–1.80)		
								ΔSJC28, median (IQR)			-4.00 (-7.00, -0.50)		-2.00 (-7.00, -1.00)			p = 0.479			
								ΔTJC28, median (IQR)			-3.00 (-6.50, -0.50)		-3.00 (-6.00, 0.00)			p = 0.668			
								ΔESR, median (IQR)			-4.50 (-16.00, 1.00)		-3.00 (-11.50, 1.00)			p = 0.59			
								ΔCRP, median (IQR)			-0.45 (-0.94, -0.10)		0.20 (-1.00, 0.00)			p = 0.389			
								RADAI-5-remission (≤ 1.4)	22 (14.7%)	32 (18.0%)							OR (95% CI): 1.05 (0.32–3.51)		
			Infliximab	73	Adalimumab	178		DAS-28-ESR remission (<2.6)	17 (23.3%)	57 (32.0%)							OR (95% CI): 0.77 (0.26–2.34)		
								DAS-28-ESR LDA (≤3.2)	31 (42.5%)	87 (48.9%)							OR (95% CI): 1.00 (0.36–2.74)		
								ΔSJC28, median (IQR)			-2.00 (-5.50, -0.50)		-2.00 (-7.00, -1.00)			p = 0.648			
								ΔTJC28, median (IQR)			-1.00 (-6.00, 0.00)		-3.00 (-6.00, 0.00)			p = 0.642			

								ΔESR, median (IQR)			**-0.50 (-5.00, 14.75)**		-3.00 (-11.50, 1.00)			**p = 0.044**			
								ΔCRP, median (IQR)			**0.35 (-0.24, 0.50)**		0.20 (-1.00, 0.00)			**p = 0.047**			
								RADAI-5-remission (≤ 1.4)	10 (13.7%)	32 (18.0%)							OR (95% CI): 0.64 (0.15–2.80)		
			Abatacept	42	Adalimumab	178		DAS-28-ESR remission (<2.6)	16 (38.1%)	57 (32.0%)							OR (95% CI): 0.61 (0.16–2.25)		
								DAS-28-ESR LDA (≤3.2)	23 (54.8%)	87 (48.9%)							OR (95% CI): 0.72 (0.19–2.74)		
								ΔSJC28, median (IQR)			-4.00 (-7.00, -2.00)		-2.00 (-7.00, -1.00)			p = 0.562			
								ΔTJC28, median (IQR)			-6.00 (-10.00, -0.75)		-3.00 (-6.00, 0.00)			p = 0.131			
								ΔESR, median (IQR)			-1.00 (-8.00, 2.00)		-3.00 (-11.50, 1.00)			p = 0.574			
								ΔCRP, median (IQR)			-0.23 (-0.59, 0.00)		0.20 (-1.00, 0.00)			p = 0.366			
								RADAI-5-remission (≤ 1.4)	9 (21.4%)	32 (18.0%)							OR (95% CI): 5.43 (0.96–30.87)		
Baker 2025	Observational cohort study	Metabolic Syndrome	TNFi	383			6M	MCID for CDAI (with metabolic syndrome vs without)									OR (95% CI) 0.65 (0.49–0.87)	Metabolic syndrome was associated with a lower likelihood of MCID in CDAI. This negative association was observed for both TNF inhibitors and non-TNFi therapies), with no significant interaction between treatment classes.	Moderate
			Non-TNFi (except JAKi)	304			6M	MCID for CDAI (with metabolic syndrome vs without)									OR (95% CI) 0.76 (0.55–1.04)		
**Studies regarding Safety**	-	-	-	-	-	-	-	-	-	-	-	-	-	-	-	-	-	-	-
Balsa 2023	Pooled analysis of RCT data	Obesity	Filgotinib 200	NA	Filgotinib 100	NA		TEAEs	555 (83.1%)	393 (78.9%)									High
								Serious TEAEs	107 (16.0%)	70 (14.1%)									
								Serious infection	27 (4.0%)	19 (3.8%)									
								Venous thrombotic and embolic events	7 (1.0%)	2 (0.4%)									
								MACE	7 (1.0%)	5 (1.0%)									
								Herpes Zoster infection	27 (4.0%)	9 (1.8%)									
								Malignancies excluding NMSC	8 (1.2%)	7 (1.4%)									
								Death	7 (1.0%)	2 (0.4%)									
KARATAŞ 2023	Observational cohort study	Obesity	Rituximab 1000 mg (on days 0 and 15)	59	No comparator		Mean follow-up: 37M	AE leading to treatment discontinuation	3 (5.1)									Adverse events leading to treatment discontinuation were reported during a mean follow-up of 37 months; only 3 patients (5.1%) discontinued due to AE, including 1 (5.9%) due to infection.	Moderate
								infection leading to discontinuation	1 (5.9)										
**Cardiovascular Disease (CVD)**																			
**Studies regarding efficacy**	-	-	-	-	-	-	-	-	-	-	-	-	-	-	-	-	-	-	
Specker 2022	Observational cohort study	Coronary Artery Disease (CAD)	Tocilizumab	118	No comparator		Follow-up until 104W	ΔDAS28-ESR			-2.0 (1.8)	p = 0.50							High
Sagtaganov 2024	Cross-sectional	CVD	Yoga	24	Kinesiotherapy, balneotherapy, and pilates	34	-	Morning stiffness, median (IQR)			70.00 (60.00–70.00)		90.00 (80.00–100.00)			p < 0.001		RA patients with cardiovascular disease practicing yoga experienced lower pain levels, reduced morning stiffness duration, and lower mean DAS-28 scores, demonstrating greater improvements in disease activity and symptom relief compared to those undergoing kinesiotherapy, balneotherapy, or Pilates.	High
								VAS pain (mm), median (IQR)			30.00 (20.00–30.00)		40.00 (32.50–50.00)			p < 0.001			
								DAS28, median (IQR)			5.67 (5.25–5.85)		6.34 (6.11–6.78)			p < 0.001			
**Studies regarding Safety**	-	-	-	-	-	-	-	-	-	-	-	-	-	-	-	-	-	-	-
Khosrow-Khavar 2022	Observational cohort study	History of CVD	Tofacitinib	-	TNFi	-	-	CV outcome (hospitalisations for MI or stroke)									pooled wHR (95% CI): 1.27 (0.95 to 1.70)		High
Charles Schoeman 2023	Post-hoc analysis (ORAL Surveillance)	History of ASCVD	Tofacitinib 5 mg	204	TNFis	214		MI (fatal/ non-fatal) IR per 100 PY (95% CI)	1.01 (0.41, 2.08)	0.42 (0.09, 1.24)							HR (95% CI): 2.35 (0.61, 9,10)		High
								Stroke (fatal/ nonfatal) IR per 100 PY (95% CI)	1.17 (0.51, 2.32)	0.42 (0.09, 1.24)							HR (95% CI): 2.80 (0.74, 10.56)		
			Tofacitinib 10 mg	222	TNFis	214		MI (fatal/ non-fatal) IR per 100 PY (95% CI)	1.07 (0.43, 2.20)	0.42 (0.09, 1.24)							HR (95% CI): 2.55 (0.66, 9.87)		
								Stroke (fatal/ nonfatal) IR per 100 PY (95% CI)	0.9 (0.33, 1.96)	0.42 (0.09, 1.24)							HR (95% CI): 2.10 (0.53, 8.41)		
Curtis 2023	Post-hoc analysis RCT (ORAL Surveillance)	History of ASCVD	Tofacitinib 5 mg	204	TNFis	214		lung cancer IR per 100 PY (95% CI)	0.40 (0.08–1.16)	0.25 (0.03–0.89)							HR (95% CI):2.13 (0.45–10.03)		High
			Tofacitinib 10 mg	222	TNFis	214		lung cancer IR per 100 PY (95% CI)	0.66 (0.21–1.54)	0.25 (0.03–0.89)							HR (95% CI): 1.61 (0.27–9.63)		
Buch 2024	Post-hoc analysis (ORAL Surveillance)	History of ASCVD	Tofacitinib 5 mg	204	TNFis	214		MACE IR per 100 PY (95% CI)	2.53 (1.47, 4.05)	1.28 (0.59, 2.44)							HR (95% CI): 1.96 (0.87, 4.40)	Results showed a numerically higher risk of extended MACE with tofacitinib versus TNFi in patients with a history of ASCVD.	High
								MACE8 IR per 100 PY (95% CI)	4.95 (3.38, 6.99)	3.68 (2.38, 5.64)							HR (95% CI): 1.34 (0.80, 2.27)		
								MACE8 + VTE IR per 100 PY (95% CI)	5.11 (3.52, 7.18)	4.33 (2.90, 6.21)							HR (95% CI): 1.19 (0.72, 1.95)		

								MACE IR per 100 PY (95% CI)	2.61 (1.52, 4.18)	1.28 (0.59, 2.44)							HR (95% CI): 2.01 (0.89, 4.50)		
			Tofacitinib 10 mg	222	TNFis	214		MACE8 IR per 100 PY (95% CI)	4.93 (3.35, 6.99)	3.68 (2.38, 5.44)							HR (95% CI): 1.32 (0.78, 2.24)		
								MACE8 + VTE IR per 100 PY (95% CI)	6.15 (4.35, 8.44)	4.33 (2.90, 6.21)							HR (95% CI): 1.41 (0.87, 2.28)		
			Tofacitinib combined doses	426	TNFis	214		MACE IR per 100 PY (95% CI)	2.57 (1.78, 3.59)	1.28 (0.59, 2.44)							HR (95% CI): 1.98 (0.95, 4.14)		
Aymon 2025	Observational cohort study	History of CVD	JAKi	-	TNFi	-	Median follow-up: 1.3 Y; total PY JAKi: 2,827, total PY TNFi: 5,517	MACE IR (95% CI) per 1000 PY (pooled analysis)	6.72 (4.05–10.50)	1.16 (0.67–2.00)							aIRR (95% CI): 1.06 (0.56–2.02)		Moderate
Cho 2025	Observatinal cohort study	History of CVD (Ischemic heart disease, peripheral vascular disorder, or cerebrovascular disease)	JAKi	-	TNFi	-	Median follow-up: 1.84 Y JAKi and 1.58 Y TNFi	MACE IR per 100 PY	13.24	7.6							HR (95% CI): 1.29 (0.59-2.81)	In patients with a history of CVD, JAK inhibitor use was associated with a nonsignificant increase in 3-point MACE risk compared with TNF inhibitors consistent with the trend observed in the ORAL Surveillance trial.	Moderate
You 2025	Observational cohort study	History of CVD	JAKi	293	TNFi	897		MACE IR per 100 PY (95% CI)	3.07 (1.70–5.54)	3.44 (2.66–4.45)							HR (95% CI): 0.79 (0.41–1.52)	No significant differences in the incidence of MACE, or in its individual components (MI, stroke, and HF in this study), were observed between JAKi and TNFi among patients with a history of CVD.	High
								MI IR per 100 PY (95% CI)	0.28 (0.04–1.98)	0.41 (0.20–0.87)							HR (95% CI): 0.80 (0.10–6.26)		
								Stroke IR per 100 PY (95% CI)	1.12 (0.42–2.97)	1.48 (1.00–2.19)							HR (95% CI): 0.57 (0.20–1.63)		
								HF IR per 100 PY (95% CI)	1.67 (0.75–3.73)	1.78 (1.24–2.54)							HR (95% CI): 0.91 (0.36–2.30)		
**Risk Factors**																			
**Studies regarding Safety**	-	-	-	-	-	-	-	-	-	-	-	-	-	-	-	-	-	-	-
Khosrow-Khavar 2022	Observational cohort study	CV risk factor	Tofacitinib	3497	TNFi	31 573	Total PY of follow up, TOFA: 3159; TNFi: 29030	CV outcome (hospitalisations for MI or stroke)									wHR (95% CI): 1.24 (0.90–1.69)		High
Taylor 2022	Pooled analysis of RCT data	Overall population (54.8% of patients had ≥1 CV risk factor)	Baricitinib (all patients who received ≥1 dose)	1780				MACE IR (95% CI)	0.70 (0.53 to 0.92)										High
		Aged ≥50 with CV risk factor	Baricitinib (all patients who received ≥1 dose)					MACE IR (95% CI)	0.77 (0.56 to 1.04)										
Ytterberg 2022	RCT (ORAL Surveillance)	CV risk factor	Tofacitinib 5 mg	1455 (PY = 5166.32)	TNFis	1451 (PY = 5045.27)	4 Y (median follow up)	MACE IR per 100 PY (95% CI)	0.91 (0.67–1.21)	0.73 (0.52–1.01)	-	-	-	-	-	-	HR (95% CI): 1.24 (0.81–1.91)	Risks of MACE and cancers were higher with tofacitinib and did not meet noninferiority criteria.	High
				1455 (PY = 5491.48)	TNFis	1451 (PY = 5482.30)		Malignancies Excluding NMSC IR per 100 PY (95% CI)	1.13 (0.87–1.45)	0.77 (0.55–1.04)	-	-	-	-	-	-	**HR (95% CI): 1.47 (1.00–2.18)**		
			Tofacitinib 10 mg	1456 (PY = 4871.96)	TNFis	1451 (PY = 5045.27)		MACE IR per 100 PY (95% CI)	1.05 (0.78–1.38)	0.73 (0.52–1.01)	-	-	-	-	-	-	HR (95% CI): 1.43 (0.94–2.18)		
				1456 (PY = 5311.71)	TNFis	1451 (PY = 5482.30)		Malignancies Excluding NMSC IR per 100 PY (95% CI)	1.13 (0.86–1.45)	0.77 (0.55–1.04)	-	-	-	-	-	-	**HR (95% CI): 1.48 (1.00–2.19)**		
			Combined tofacitinib doses	2911 (PY = 10,038.28)	TNFis	1451 (PY = 5045.27)		MACE IR per 100 PY (95% CI)	0.98 (0.79-1.19)	0.73 (0.52–1.01)	-	-	-	-	-	-	HR (95% CI): 1.33 (0.91-1.94)		
				2911 (PY = 10,803.19)	TNFis	1451 (PY = 5482.30)		Malignancies Excluding NMSC IR per 100 PY (95% CI)	1.13 (0.94-1.35)	0.77 (0.55–1.04)	-	-	-	-	-	-	**HR (95% CI):1.48 (1.04-2.09)**		
		Age ≥65 years old and CV risk factor	Tofacitinib 5 mg	413	TNFis	462		MACE IR per 100 PY (95% CI)	1.38 (0.83, 2.15)	0.91 (0.50, 1.52)	-	-	-	-	-	-	HR (95% CI): 1.51 (0.76, 3.02)		
			Tofacitinib 10 mg	478	TNFis	462		MACE IR per 100 PY (95% CI)	1.68 (1.24, 2.74)	0.91 (0.50, 1.52)	-	-	-	-	-	-	**HR (95% CI): 2.06 (1.08, 3.93)**		
			Combined tofacitinib doses	891	TNFis	462		MACE IR per 100 PY (95% CI)	1.63 (1.20 to 2.18)	0.91 (0.50, 1.52)	-	-	-	-	-	-	HR (95% CI): 1.79 (0.99 to 3.26)		
			Combined tofacitinib doses	891	TNFis	462		Malignancies Excluding NMSC IR per 100 PY (95% CI)	1.79 (1.35 to 2.33)	1.05 (0.62 to 1.67)	-	-	-	-	-	-	**HR (95% CI): 1.70 (1.00 to 2.90)**		
Charles Schoeman 2023	Post-hoc analysis (ORAL Surveillance)	CV risk factor	Tofacitinib 5 mg	1455	TNFis	1451	-	MI (fatal/ non-fatal) IR per 100 PY (95% CI)	0.37 (0.22, 0.57)	0.22 (0.11, 0.39	-	-	-	-	-	-	HR (95% CI): 1.69 (0.80, 3.55)		High
							-	Stroke (fatal/ nonfatal) IR per 100 PY (95% CI)	0.35 (0.21, 0.55)	0.34 (0.20, 0.54)	-	-	-	-	-	-	HR (95% CI): 1.03 (0.53, 2.00)		
			Tofacitinib 10 mg	1456	TNFis	1451	-	MI (fatal/ non-fatal) IR per 100 PY (95% CI)	0.39 (0.23, 0.61)	0.22 (0.11, 0.39	-	-	-	-	-	-	HR (95% CI): 1.80 (0.85, 3.77)		
							-	Stroke (fatal/ nonfatal) IR per 100 PY (95% CI)	0.37 (0.22, 0.58)	0.34 (0.20, 0.54)	-	-	-	-	-	-	HR (95% CI): 1.10 (0.57, 2.13)		
Curtis 2023		CV risk factor	Tofacitinib 5 mg	1455	TNFis	1451		Malignancies excluding NMSC	62 (4.26%)								**HR (95% CI): 1.47 (1.00–2.18)**		High

			Tofacitinib 10 mg	1456	TNFis	1451		Malignancies excluding NMSC	60 (4.12%)								**HR (95% CI): 1.48 (1.00–2.19)**		
	Post-hoc analysis (ORAL Surveillance)	Current smoker	Tofacitinib 5 mg	411	TNFis	353		lung cancer IR per 100 PY (95% CI)	0.58 (0.27–1.10)								HR (95% CI): 1.94 (0.60–6.29)		
			Tofacitinib 10 mg	402	TNFis	353		lung cancer IR per 100 PY (95% CI)	0.55 (0.24–1.08)								HR (95% CI): 1.83 (0.55–6.09)		
		Past smoker	Tofacitinib 5 mg	309	TNFis	326		lung cancer IR per 100 PY (95% CI)	0.34 (0.09–0.88)								HR (95% CI): 1.41 (0.32–6.29)		
			Tofacitinib 10 mg	302	TNFis	326		lung cancer IR per 100 PY (95% CI)	0.65 (0.26–1.33)								HR (95% CI): 2.67 (0.69–10.32)		
Kristensen 2023	Post-hoc analysis (ORAL Surveillance)	Age ≥65 year old	Tofacitinib (5mg and 10 mg combined)	891	TNFis	462		MACE									HR (95% CI): 1.79 (0.99, 3.26)		High
								MI									HR (95% CI): 3.28 (0.97, 11.14)		
								VTE									**HR (95% CI): 4.38 (1.32, 14.56)**		
								All-cause death									**HR (95% CI): 2.12 (1.02, 4.41)**		
		Current smoker	Tofacitinib (5mg and 10 mg combined)	813	TNFis	353		MACE									HR (95% CI): 1.20 (0.65, 2.21)		
								MI									HR (95% CI): 1.55 (0.57, 4.16)		
								VTE									HR (95% CI): 5.08 (0.66, 39.07)		
								All-cause death									HR (95% CI): 1.55 (0.57, 4.17)		
		Past smoker	Tofacitinib (5mg and 10 mg combined)	611	TNFis	326		MACE									HR (95% CI): 2.17 (0.95, 4.96)		
								MI									HR (95% CI): 2.68 (0.59, 12.22)		
								VTE									**HR (95% CI): 5.13 (1.19, 22.02)**		
								All-cause death									**HR (95% CI): 3.38 (1.18, 9.71)**		
Buch 2024	Post-hoc analysis (ORAL Surveillance)	CV risk factor	Tofacitinib 5 mg	1455	TNFis	1451		MACE8 IR per 100 PY (95% CI)	1.41 (1.10, 1.77)	1.34 (1.04, 1,70)							HR (95% CI) vs TNFi: 1.05 (0.75, 1.46)	In patients with CV risk factors, the risk of MACE-8 plus VTE was similar for tofacitinib 5 mg BID vs. TNFi but higher with tofacitinib 10 mg BID.	High
								MACE8 + VTE IR per 100 PY (95% CI)	87 (6.0%)	1.53 (1.20, 1.91)							HR (95% CI) vs TNFi: 1.12 (0.82, 1.52)		
			Tofacitinib 10 mg	1456	TNFis	1451		MACE8 IR per 100 PY (95% CI)	1.49 (1.16, 1.87)	1.34 (1.04, 1,70)							HR (95% CI) vs TNFi: 1.11 (0.79, 1.55)		
								MACE8 + VTE IR per 100 PY (95% CI)	101 (6.9%)	1.53 (1.20, 1.91)							**HR (95% CI) vs TNFi: 1.38 (1.02, 1.85)**		
		Age ≥65 year old and CV risk factor	Tofacitinib 5 mg	1455	TNFis	1451		MACE IR per 100 PY (95% CI)	1.38 (0.83, 2.15)	0.91 (0.50, 1.52)							HR (95% CI) vs TNFi: 1.51 (0.76, 3.02)	Patients aged ≥65 years had a higher risk of MACE 3, 8 and MACE8 + VTE with tofacitinib compared to TNFi as reference, and the risk was significantly higher for the 10 mg twice daily dose.	
								MACE8 IR per 100 PY (95% CI)	1.97 (1.30, 2.86)	1.70 (1.11, 2.49)							HR (95% CI) vs TNFi: 1.16 (0.67, 1.98)		
								MACE8 + VTE IR per 100 PY (95% CI)	2.57 (1.79, 3.69)	1.91 (1.28, 2.74)							HR (95% CI) vs TNFi: 1.35 (0.82, 2.20)		
			Tofacitinib 10 mg	1456	TNFis	1451		MACE IR per 100 PY (95% CI)	1.88 (1.24, 2.74)	0.91 (0.50, 1.52)							**HR (95% CI) vs TNFi: 2.06 (1.08, 3.93)**		
								MACE8 IR per 100 PY (95% CI)	2.82 (2.02, 3.94)	1.70 (1.11, 2.49)							**HR (95% CI) vs TNFi: 1.65 (1.01, 2.71)**		
								MACE8 + VTE IR per 100 PY (95% CI)	3.73 (2.78, 4.89)	1.91 (1.28, 2.74)							**HR (95% CI) vs TNFi: 1.95 (1.24, 3.07)**		
Fleischmann 2023	Integrated post hoc analysis of RCTs	CV risk factor	Upadacitinib 15mg ±csDMARD(s)	1717	Adalimumab 40mg+MTX	320		serious infection EAIR (95% CI) [n/100 PY]	3.9 (3.3, 4.5)	3.0 (1.9, 4.6)									High
								Malignancies EAIR (95% CI) [n/100 PY] (excl. NMSC)	1.1 (0.8, 1.4)	1.5 (0.8, 2.6)									
								herpes zoster EAIR (95% CI) [n/100 PY]	3.4 (2.9, 4.0)	1.2 (0.5, 2.2)									
								MACE EAIR (95% CI) [n/100 PY]	0.6 (0.4, 0.8)	0.5 (0.1, 1.3)									
								VTE EAIR (95% CI) [n/100 PY]	0.6 (0.4, 0.9)	0.6 (0.2, 1.5)									

	Post-hoc analysis (SELECT-COMPARE study)	CV risk factor	Upadacitinib 15mg+MTX	649	Adalimumab 40mg+MTX	177		serious infection EAIR (95% CI) [n/100 PY]	3.7 (2.9, 4.7)	3.7 (1.9, 6.5)							HR 1.04 (95% CI 0.56, 1.93)		
								Malignancies EAIR (95% CI) [n/100 PY] (excl. NMSC)	0.7 (0.4, 1.2)	1.8 (0.6, 3.9)							HR 0.37 (95% CI 0.14, 0.97)		
								herpes zoster EAIR (95% CI) [n/100 PY]	2.8 (2.1, 3.7)	0.9 (0.2, 2.6)							**HR 3.07 (95% CI 0.96, 9.88)**		
								MACE EAIR (95% CI) [n/100 PY]	0.4 (0.2, 0.8)	0.6 (0.1, 2.1)							HR 0.63, 95% CI 0.13, 3.07		
								VTE EAIR (95% CI) [n/100 PY]	0.3 (0.1, 0.6)	0.9 (0.2, 2.6)							HR 0.37 (95% CI: 0.09, 1.54)		
								death EAIR (n/100 PY)	1.4	1.5							HR 0.91 (95% CI 0.35, 2.38)		
Haraoui 2024	Observational cohort study	CV risk factor: ≥50 yrs + ≥1 CV risk (current smoker, HTN, diabetes mellitus, history of CAD, or RA associated extra-articular disease)	Tofacitinib 5 mg BID (some 10 mg BID)	272			Up to 36 M	MACE IR per 100 PY (95% CI)	1.6 (0.6, 3.3)									Patients with cardiovascular risk factor had higher rates of adverse events of special interest such as MACE, malignancy, and serious infection, while tofacitinib effectiveness remained comparable between those with and without cardiovascular risk	High
								VTE IR per 100 PY (95% CI)	0.5 (0.1, 1.7)										
								Serious infection IR per 100 PY (95% CI)	5.5 (3.5, 8.2)										
								HZ (non-serious/serious) IR per 100 PY (95% CI)	1.4 (0.5, 3.0)										
								Malignancies (excluding NMSC) IR per 100 PY (95% CI)	2.1 (1.0, 4.0)										
							6 M	CDAI remission	12.00%										
								DAS28-ESR remission	16.50%										
								DAS28-ESR LDA	31.00%										
								HAQ-DI (mean change)			− 0.3 (− 0.4, − 0.2)								
Priora 2024	Observational cohort study (BIRRA project)	CV risk factor	Tofacitinib	129			6 M	ΔDAS28-ESR			−1.9			<0.001				Efficacy was similar between High and lower-risk patients, although older age slightly reduced the magnitude of improvement.	High
								ΔDAS28-CRP			−2.1			<0.001					
							12 M	ΔDAS28-ESR			−2.2			<0.001					
								ΔDAS28-CRP			−2.6			<0.001					
Aymon 2025	Observational cohort study	Age ≥50 years with CV risk factor	JAKi	7876	TNFi	7876	Median follow-up: 1.3 Y; total PY JAKi: 9,027, TNFi: 21,628	MACE IR (95% CI) per 1000 PY (within-registry analysis)	10.08 (8.12–12.38)	11.93 (10.52–13.48)							aIRR (95% CI) 0.57 (0.12–2.66)		Moderate
							Median follow-up: 1.3 Y; total PY JAKi: 11,440 , TNFi: 21,136	MACE IR (95% CI) per 1000 PY (pooled data analysis)	3.58 (2.57–4.86)	4.45 (3.59–5.44)							aIRR (95% CI) 1.11 (0.55–2.22)		
Burmester 2025	Post-hoc benefit-risk analysis (SELECT-COMPARE)	High CV risk (≥65 years of age and/or ≥1 CV risk factor: prior history of a CV event, hypertension, diabetes mellitus, current or former smoker, age ≥65 years, elevated baseline low- density lipoprotein cholesterol (LDL- C; ≥3.36 mmol/L) and lowered baseline high- density lipoprotein cholesterol (HDL- C; <1.034 mmol/L))	UPA 15 mg QD+MTX	1106 (PY = 321.2)	ADA 40 mg EOW+MTX	458 (PY = 135.5)	6M	serious infection EAER (95% CI)/100 PY	5.6 (3.3, 8.9)	5.2 (2.1, 10.6)									High
			UPA 15 mg QD+MTX	1107 (PY = 3368.5)	ADA 40 mg EOW+MTX	458 (PY = 1191.6)	5Y	serious infection EAER (95% CI)/100 PY	4.4 (3.7, 5.1)	3.3 (2.3, 4.5)									
			UPA 15 mg QD+MTX	1106 (PY = 321.2)	ADA 40 mg EOW+MTX	458 (PY = 135.5)	6M	Herpes zoster EAER (95% CI)/100 PY	2.5 (1.1, 4.2)	1.5 (0.2, 5.3)									
			UPA 15 mg QD+MTX	1107 (PY = 3368.5)	ADA 40 mg EOW+MTX	458 (PY = 1191.6)	5Y	Herpes zoster EAER (95% CI)/100 PY	3.1 (2.5, 3.7)	1.1 (0.6, 1.9)									
			UPA 15 mg QD+MTX	1106 (PY = 321.2)	ADA 40 mg EOW+MTX	458 (PY = 135.5)	6M	Malignancies EAER (95% CI)/100 PY (excl. NMSC)	0 (0, 1.1)	0 (0, 2.7)									
			UPA 15 mg QD+MTX	1107 (PY = 3368.5)	ADA 40 mg EOW+MTX	458 (PY = 1191.6)	5Y	Malignancies EAER (95% CI)/100 PY (excl. NMSC)	0.7 (0.5, 1.1)	1.0 (0.5, 1.8)									
			UPA 15 mg QD+MTX	1106 (PY = 321.2)	ADA 40 mg EOW+MTX	458 (PY = 135.5)	6M	NMSC EAER (95% CI)/100 PY	0.3 (0, 1.7)	0.7 (0, 4.1)									
			UPA 15 mg QD+MTX	1107 (PY = 3368.5)	ADA 40 mg EOW+MTX	458 (PY = 1191.6)	5Y	NMSC EAER (95% CI)/100 PY	0.7 (0.4, 1.0)	0.2 (0, 0.6)									
			UPA 15 mg QD+MTX	1106 (PY = 321.2)	ADA 40 mg EOW+MTX	458 (PY = 135.5)	6M	MACE EAER (95% CI)/100 PY	0 (0, 1.1)	1.5 (0.2, 5.3)									
			UPA 15 mg QD+MTX	1107 (PY = 3368.5)	ADA 40 mg EOW+MTX	458 (PY = 1191.6)	5Y	MACE EAER (95% CI)/100 PY	0.4 (0.2, 0.6)	0.3 (0.1, 0.9)									
			UPA 15 mg QD+MTX	1106 (PY = 321.2)	ADA 40 mg EOW+MTX	458 (PY = 135.5)	6M	VTE (95% CI)/100 PY	0.6 (0.1, 2.2)	2.2 (0.5, 6.5)									
			UPA 15 mg QD+MTX	1107 (PY = 3368.5)	ADA 40 mg EOW+MTX	458 (PY = 1191.6)	5Y	VTE (95% CI)/100 PY	0.3 (0.2, 0.6)	0.5 (0.2, 1.1)									
			UPA 15 mg QD+MTX	522	ADA 40 mg EOW+MTX	245	6M	DAS28(CRP) < 2.6 response rate (95% CI)	38.5 (34.3, 42.7)	26.1 (20.6, 31.6)						p<0.001			

			UPA 15 mg QD+MTX	522	5Y	245	5Y	DAS28(CRP) < 2.6 response rate (95% CI)	29.1 (25.2, 33.0)	23.7 (18.4, 29.0)									
			UPA 15 mg QD+MTX	522	ADA 40 mg EOW+MTX	245	6M	CDAI < 2.8 response rate (95% CI)	20.9 (17.4, 24.4)	14.3 (9.9, 18.7)						p<0.05			
			UPA 15 mg QD+MTX	522	5Y	245	5Y	CDAI < 2.8 response rate (95% CI)	23.4 (19.7, 27.0)	17.1 (12.4, 21.9)									
			UPA 15 mg QD+MTX	522	ADA 40 mg EOW+MTX	245	6M	DAS28(CRP) < 3.2 response rate (95% CI)	51.9 (47.6, 56.2)	39.2 (33.1, 45.3)						p<0.001			
			UPA 15 mg QD+MTX	522	5Y	245	5Y	DAS28(CRP) < 3.2 response rate (95% CI)	31.6 (27.6, 35.6)	25.7 (20.2, 31.2)									
			UPA 15 mg QD+MTX	522	ADA 40 mg EOW+MTX	245	6M	CDAI < 10 response rate (95% CI)	50.4 (46.1, 54.7)	39.2 (33.1, 45.3)						p<0.01			
			UPA 15 mg QD+MTX	522	5Y	245	5Y	CDAI < 10 response rate (95% CI)	33.9 (29.8, 38.0)	27.3 (21.8, 32.9)									
			UPA 15 mg QD+MTX	522	ADA 40 mg EOW+MTX	245	6M	Change from BL in HAQ- DI ≤ -0.22 (95% CI)	58.8 (54.6, 63.0)	52.7 (46.4, 58.9)									
			UPA 15 mg QD+MTX	522	5Y	245	5Y	Change from BL in HAQ- DI ≤ -0.22 (95% CI)	31.4 (27.4, 35.4)	24.9 (19.5, 30.3)									
Cho 2025	Observational cohort study	aged ≥50 with CV risk factor	JAKi		TNFi			MACE IR per 100 PY	6.85	7.22							HR (95% CI): 0.92 (0.52-1.64)	In patients aged 50 years or older with at least one CV risk factor, the risks of MACE was generally comparable between JAKi and TNFi users	Moderate
Mariette 2025	Pooled analysis of RCT data	≥ 65 years age or ≥ 1 CV risk factor	Filgotinib 100mg	1008	Filgotinib 200 mg	1278	3.8 Y (Median exposure duration)	MACE EAIR (95% CI)/100 PYE	0.7 (0.4, 1.0)	0.6 (0.4, 0.9)								Among patients aged ≥ 65 years or with ≥ 1 cardiovascular risk factor, MACE rates were numerically higher. No dose dependent effect was observed.	High
								VTE EAIR (95% CI)/100 PYE	0.3 (0.1, 0.5)	0.3 (0.2, 0.5)									
		≥ 65 years age	Filgotinib 100mg	326	Filgotinib 200 mg	407		MACE EAIR (95% CI)/100 PYE	1.0 (0.5, 1.9)	1.0 (0.5, 1.7)									
								VTE EAIR (95% CI)/100 PYE	0.2 (0.0, 0.8)	0.3 (0.1, 0.8)									
		< 65	Filgotinib 100mg	1321	Filgotinib 200 mg	1880		MACE EAIR (95% CI)/100 PYE	0.4 (0.2, 0.6)	0.2 (0.1, 0.4)									
								VTE EAIR (95% CI)/100 PYE	0.2 (0.1, 0.4)	0.2 (0.1, 0.3)									
		≥50 years with CV risk factors (current smoker or family/medical history)	Filgotinib 100mg	692	Filgotinib 200 mg	870		MACE EAIR (95% CI)/100 PYE	0.7 (0.4–1.2)	0.7 (0.4–1.0)									
Kao 2025	Retrospective cohort study	⩾ 65 years age	tsDMARDs (mostly TOFA)		TNFis		Up to 5 Y	MACE									aHR (95% CI): 1.43 (0.87–2.35)	No association between MACE and tsDMARDs compared with TNF inhibitors was foun after adjustment, consistent with patients with high CV risk.	Moderate
		High CV risk	tsDMARDs (mostly TOFA)	495	TNFis	2735		MACE									aHR (95% CI): 1.03 (0.62–1.71)		
You 2025	Observational cohort study	High CV risk	JAKi	939	TNFi	3026		MACE IR per 100 PY (95% CI)	1.32 (0.81-2.15)	1.52 (1.23-1.86)							HR (95% CI): 0.77 (0.45-1.34)	No significant differences in the incidence of MACE, or in its individual components (MI, stroke, and HF in this study), were observed between JAKi and TNFi across high CV risk groups.	High
								MI IR per 100 PY (95% CI)	0.16 (0.04–0.66)	0.17 (0.09–0.31)							HR (95% CI): 1.31 (0.29-5.96)		
								Stroke IR per 100 PY (95% CI)	0.41 (0.17–0.99)	0.62 (0.45–0.85)							HR (95% CI): 0.57 (0.22-1.46)		
								HF IR per 100 PY (95% CI)	0.74 (0.39–1.42)	0.80 (0.60–1.06)							HR (95% CI): 0.81 (0.38-1.72)		
**Hepatitis C virus Infection (HCV)**																			
**Studies regarding efficacy**	-	-	-	-	-	-	-	-	-	-	-	-	-	-	-	-	-	-	-
Lashen 2021	Observational cohort study	Chronic HCV	Sofosbuvir + daclatasvir or sofosbuvir/ledipasvir (direct-acting antivirals (DAAs))	59	No comparator		Baseline	Tender joints			3.34 (1.63)							The mean value of ESR, CRP, DAS28, TJC, SJC, PtGA after HCV elimination was significantly lower than the baseline values. The number of patients who used corticosteroids was significantly declined from 16 (27.1%) patients before DAAs to 4 (6.8%) patients after DAAs therapy (P < 0.001). However the difference in the mean steroid dose was not statistically significant, and this is due to the small number of patients who were maintained on steroids after HCV treatment. After sustained virologic response at 12 weeks post-treatment (SVR12), the RA disease activity was significantly improved in comparison to the pre-HCV treatment status [13.6% with high, 32.2% with moderate, 23.7% with low activity and 30.5% patients in remission after achieving SVR12 vs. 30.5% with high, 59.3% with moderate, 6.8% with low activity and 3.4% patients in remission before achieving SVR12 respectively; (p< 0.001)	High
								Swollen joints			3.64 (1.81)								
								PtGA			40.68 (15.35)								
								DAS28			4.37 (0.90)								
								ESR (mm/h)			29.68 (14.32)								
								CRP (mg/l)			11.44 (4.13)								
								Steroids n (%)			16 (27.1)								
								Steroid dose			7.50 (2.88)								
								High activity	18 (30.5)										
								Moderate activity	35 (59.3)										
								Low activity	4 (6.8)										
								Remission	2 (3.4)										
							3 M	Tender joints			1.86 (1.28)	p<0.001			1.48 (1.23–1.72)				
								Swollen joints			2.34 (1.47)	p<0.001			1.3 (1.10–1.50)				
								PtGA			22.88 (15.46)	p<0.001			17.8 (15.78–19.81)				
								DAS28			3.32 (0.93)	p<0.001			1.05 (0.94–1.15)				
								ESR (mm/h)			17.13 (10.84)	p<0.001			12.55 (10.33–14.75)				
								CRP (mg/l)			5.76 (1.57)	p<0.001			5.68 (4.67–6.69)				
								Steroids n (%)			4 (6.8)	p<0.001							
								Steroid dose			4.5 (0.58)	p=0.08			3.00 (-0.67–6.67)				
								High activity	8 (13.6)			p< 0.001							
								Moderate activity	19 (32.2)										
								Low activity	14 (23.7)										
								Remission	18 (30.5)										

**Studies regarding Safety**	-	-	-	-	-	-	-	-	-	-	-	-	-	-	-	-	-	-	-
Burton 2017	Observational cohort study	Chronic HCV	Biologic DMARDs	457	Nonbiologic DMARDs	640	12 M	Hepatotoxicity incidence	22 (4.8%)	15 (2.3%)	-		-		-	0.03		bDMARDs showed higher hepatotoxicity than non-bDMARDs. TNFi and non-TNFi had similar risks.	High
			TNFi biologics	407	Non-TNFi biologics	50	12 M	Hepatotoxicity incidence	20 (4.9%)	2 (4.0%)	-		-		-	1.00			
			SSZ/HCQ	393	MTX	156	12 M	Hepatotoxicity incidence	7 (1.8%)	6 (3.9%)	-		-		-	0.20			
			SSZ/HCQ	393	LEF	91	12 M	Hepatotoxicity incidence	7 (1.8%)	2 (2.2%)	-		-		-	0.70			
Lashen 2021	Observational cohort study	Chronic HCV	Sofosbuvir + daclatasvir or sofosbuvir/ledipasvir (direct-acting antivirals (DAAs))	59	No comparator		3 M	adverse events	0									No drug-related adverse events	High
**Hepatitis B virus Infection (HBV)**																			
**Studies regarding Efficacy**	-	-	-	-	-	-	-	-	-	-	-	-	-	-	-	-	-	-	-
Chen 2017	Observational cohort study	11.1% chronic HBV infection (HBsAG+, undetectable HBV-DNA), 65.1% resolved HBV infection (anti-HBc+, HBsAG-, undetectable HBV-DNA), 23.8% no HBV infection (anti-HBC-, HBsAG-, undetectable HBV-DNA)	Tocilizumab (8mg/kg Q4W, IV)	63	NA	NA	12W	DAS28-ESR <3.2	44 (70%)									No significant differences in response to TCZ among patients with chronic HBV infection, resolved HBV and non-HBV infection.	High
								DAS28-ESR <2.6	32 (51%)										
**Studies regarding Safety**	-	-	-	-	-	-	-	-	-	-	-	-	-	-	-	-	-	-	-
Chen 2017	Observational cohort study	11.1% chronic HBV infection (HBsAG+, undetectable HBV-DNA), 65.1% resolved HBV infection (anti-HBc+, HBsAG-, undetectable HBV-DNA), 23.8% no HBV infection (anti-HBC-, HBsAG-, undetectable HBV-DNA)	Tocilizumab (8mg/kg Q4W, IV)	63	NA	NA	12W	HBV reactivation	3 (4.8%)									3 patients with chronic HBV infection and without antiviral prophylaxis developed HBV reactivation	High
Fukuda 2019	Observational cohort study	Resolved HBV infection (HBsAg - and HBsAb + and/or HBcAb +)	glucocorticoids (≥5mg/d prednisolone)				4 Y observation	Hepatitis B virus reactivation OR (95% CI)								p = 0.1367	1.597 (0.862 – 2.958)	There were no differences in reactivation frequency among those treated with corticosteroids, biologics, and methotrexate, the OR for reactivation(0.586 [95% CI 0.264–1.300]) was lower for patients treated with methotrexate alone to those treated with corticosteroid or biologics.	High
			bDMARDs+MTX													p = 0.9766	1.015 (0.383 – 2.688)		
			bdmards alone													p = 0.5075	1.375 (0.536 – 3.530)		
			MTX alone													p = 0.1889	0.586 (0.264 – 1.300)		
Harigai 2020	Pooled analysis of RCT data	Prior HBV infection (HbcAb+)	Baricitinib 2mg or 4 mg	215	No comparator		-	Positive test HBV DNA	32 (14.9)									Among 215 patients with prior HBV infection (HBcAb+), 32 (14.9%) had detectable HBV DNA after treatment initiation, 8 (3.7%) had a single quantifiable result (≥29 IU/mL), and 4 (1.86%) met the criteria for HBV reactivation (≥100 IU/mL); however, no patient developed clinical hepatitis.	High
								reactivation of HBV	4 (1,86)										
Kuo 2020	Observational cohort study	Prior HBV infection (HBcAb+/HBsAg−)	Rituximab 1000mg	50	No comparator		Mean duration: 3.9Y	Reactivation of HBV	4 (8)									With RTX administration, four patients (8%) experienced HBV reactivation after a mean duration of 3.5 years (SD, 2.5 years). HBV reactivation was significantly more common in patients who had been previously treated with adalimumab (100% vs 39%; p = 0.02).	High
Chen 2021	Observational cohort study	Patients with resolved HBV (HBsAg− / anti-HBc+)	Tocilizumab	81	TNFis	255		HBsAg RS IR (per 1000 PY)	0.000									Abatacept and Rituximab had higher risk of HBV reactivation in patient with RA and resolved hepatitis B. (HBV reactivation definition: HBsAg+)	High
			Abatacept	69	TNFis	255		HBsAg RS IR (per 1000 PY)	9,375								aHR (95% CI): 15.394 (3.076 to 77.037)		
			Rituximab	84	TNFis	255		HBsAg RS IR (per 1000 PY)	17,717								aHR (95% CI): 35.646 (8.158 to 155.758)		
Kuo 2021	Observational cohort study	Past HBV Infection	Tocilizumab IV. 4–8 mg/kg or SC. 162 mg	97	No comparator		Median follow-up: 9Y	reactivation of HBV	4 (4.12)									7 patients were HBsAg+ (7.2%), 64 were HBsAg−/HBcAb+ (65.9%), and 26 were HBsAg−/HBcAb− (26.8%). The median follow-up was 9 years and TCZ was given for a median of 29 months. Four patients (4.1%) experienced HBV reactivation. Of the 7 HBsAg+ patients, 4 received antiviral prophylaxis and had no reactivation, while all 3 without prophylaxis (100%) experienced reactivation and hepatitis flare-up.	High
Jeong 2021	Observational cohort study	HBsAg positive	Glucocorticoids ≤10 mg of prednisolone	128				HBV reactivation									OR (95% CI) 0.807 (0.143-4.546)	In the multivariable-adjusted analysis, low-dose corticosteroids did not affect HBV reactivation in HBsAg-positive RA patients. Leflunomide significantly increased the risk.	High
			Leflunomide	12				HBV reactivation									OR (95% CI) 3.851 (1.026-14.459)		
			Hydroxychloroquine	100				HBV reactivation									OR (95% CI) 2.986 (0.788-11.305)		

			Sulfasalazine	70				HBV reactivation									OR (95% CI) 1.451 (0.552-3.817)		
			TNFi	7				HBV reactivation									OR (95% CI) 1.329 (0.204-8.642)		
Kuo 2024	Observational cohort study	HBcAb+/HBsAg−	Adalimumab	96	Etanercept	116		HBV reactivation; IR per 1000 PY	9.4	3.7							**aHR (95%CI): 3.6 (1.0-13.1)**	Compared to etanercept, rituximab abatacept and adalimumab were associated with a higher risk of HBV reactivation. (HBV reactivation definition: HBsAg+ or detectable HBV DNA)	High
			Golimumab	75	Etanercept	116		HBV reactivation; IR per 1000 PY	5	3.7							aHR (95%CI): 1.9 (0.3-10.7)		
			Abatacept	24	Etanercept	116		HBV reactivation; IR per 1000 PY	24	3.7							**aHR (95%CI): 11.4 (2.3-55.9)**		
			Rituximab	26	Etanercept	116		HBV reactivation; IR per 1000 PY	48.3	3.7							**aHR (95%CI): 14.4 (4.0-51.2)**		
			Tocilizumab	24	Etanercept	116		HBV reactivation; IR per 1000 PY	1.6	3.7							aHR (95%CI): 2.1 (0.2-19.5)		
			Tofacitinib	29	Etanercept	116		HBV reactivation; IR per 1000 PY	14.5	3.7							**aHR (95%CI): 6.5 (1.1-38.3)**		
**Latent Tuberculosis positive (LTBI positive)**																			
**Studies regarding Safety**	-	-	-	-	-	-	-	-	-	-	-	-	-	-	-	-	-	-	-
Shin 2021	Observational cohort study	Latent Tuberculosis positive	Non-TNFis (abatacept, tocilizumab)	367	TNFis (infliximab, adalimumab, golimumab and etanercept)	1879	Follow-up until 4Y	Tuberculosis, IR per 100 PY	0.45 (0.11-1.78)	0.31 (0.91-0.51)								According to the subgroup analysis, among patients with latent TB receiving chemoprophylaxis, those started non-TNFi had higher IR of TB compared to those who started TNFis (statistical significance was not reported).	High
Jung 2022	Observational cohort study	Latent tuberculosis infection (who received preventive treatment)	Infliximab	113	Etanercept	279	Median follow-up: 803 days	Tuberculosis, IR per 100,000 PY	1236.2 (307.4–3204.4)	513.9 (127.8–1332.0)							aIRR (95% CI) 2.54 (0.50–12.96)	No statistically significant differences in the risk of tuberculosis were found among biologic therapies in RA patients who received LTBI prophylaxis. Infliximab showed the highest TB risk (IRR 2.54, 95% CI: 0.50–12.96), but was not statistically significant.	Moderate
			Adalimumab	298	Etanercept	279	Median follow up:506 days	Tuberculosis, IR per 100,000 PY	597.7 (148.6–1549.4)	513.9 (127.8–1332.0)							aIRR (95% CI) 0.93 (0.18–4.70)		
			Golimumab	182	Etanercept	279	Median follow up:549 days	Tuberculosis, IR per 100,000 PY	0	513.9 (127.8–1332.0)							-		
			Tocilizumab	296	Etanercept	279	Median follow up:668 days	Tuberculosis, IR per 100,000 PY	882.2 (316.3–1896.0)	513.9 (127.8–1332.0)							aIRR (95% CI) 1.35 (0.32–5.80)		
**Renal insufficiency and Chronic Kidney Disease (CKD)**																			
**Studies regarding efficacy**	-	-	-	-	-	-	-	-	-	-	-	-	-	-	-	-	-	-	-
Specker 2022	Observational cohort study	Renal Insufficiency	Tocilizumab	77	No comparator		Follow-up until 104W	ΔDAS28-ESR			-2.0 (1.8)	p = 0.50							High
Maeyama 2024	Observational cohort study	Renal dysfunction	Baricitinib 2mg	30	Baricitinib 4 mg	30	52W	TJC28			1.1 (0.9)	1.8 (3.5)				p > 0.05			High
								SJC28			0.5 (0.8)	0.7 (1.7)				p > 0.05			
								EGA			11.1 (15.3)	14.3 (15.3)				p > 0.05			
								PtGA			12.4 (13.0)	20.0 (18.4)				p > 0.05			
			Baricitinib 2mg	53	Baricitinib 4 mg	86		Infections and infestation events	3 (5.7%)	17 (19.8%)									
								Respiratory, thoracic and mediastinal disorder events	0 (0.0%)	9 (10.5%)									
								Hepatobiliary disorders events	2 (3.8%)	6 (7.0%)									
**Studies regarding Safety**	-	-	-	-	-	-	-	-	-	-	-	-	-	-	-	-	-	-	-
Lee 2020	Observational cohort study	Reduced renal function	MTX ; mean dose 11 mg (SD 3.2)	120	No comparator		Median follow-up: 21 M	Serious infection	10 (8.3)									Overall MTX toxicity occurred in 35 (29.2%) patients. Leukopenia (10%, 12/120) was the most commonly observed event, followed by serious infection (8.3%, 10/120), hepatotoxicity (7.5%, 9/120).	High
								Hematologic Abnormalities	25 (20.8)										
								Renal toxicity	7 (5.8)										
								Hepatotoxicity	9 (7.5)										
								Gastrointestinal events - Mucositis	7 (5.8)										
Mitsuboshi 2021	Observational cohort study	CKD	Methotrexate + folic acid	67	Methotrexate	21		Haematological toxicities	3 (14%)	33 (49%)						p = < 0.01	OR (95%CI): 0.29 (0.01–0.85)	Folic acid use significantly reduced haematological toxicities in MTX users with CKD (OR = 0.29, 95% CI: 0.01–0.85, p < 0.01), suggesting a protective effect.	High
Yokoyama 2023	Observational cohort study	Renal dysfunction (eGFR ≥ 60 and < 90)	Golimumab	223	No comparator			SAE IR per 100 PY	8.6									The frequency of typical serious adverse events due to Golimumab exposure in RA patients with renal dysfunction is independent of the degree of renal dysfuncion (eGFR ≥ 60 and < 90 or eGFR ≥ 30 and < 60).	Moderate
		Renal dysfunction (eGFR ≥ 30 and < 60)	Golimumab	115	No comparator			SAE IR per 100 PY	10.1										
Nakayama 2024	Observational cohort study	CKD (eGFR 45–60 mL/min/1.73 m2)	TNFi	200	JAKi	96	12 M	Drug discontinuation due to AEs	93.2%	91.4%							aHR (95% CI): 0.53 (0.26–1.08)(discontinuation due to toxic AEs)	In RA patients with CKD (eGFR 45–60 mL/min/1.73 m²), JAK inhibitors (Tofacitinib, Baricitinib, Upadacitinib, Filgotinib) had a comparable 12-month	High

			IL-6Ri	126	JAKi	96		Drug discontinuation due to AEs	93.8%	91.4%							aHR (95% CI): 0.60 (0.28–1.26) (discontinuation due to toxic AEs)	retention rate due to adverse events (AEs) as TNFi, IL-6Ri, and Abatacept. However, in patients with eGFR < 45 mL/min/1.73 m², JAKi showed the lowest AE-related retention, while TNFi, IL-6Ri, and Abatacept had significantly better AE profiles.	
			Abatacept	129	JAKi	96		Drug discontinuation due to AEs	95.0%	91.4%							aHR (95% CI): 0.52 (0.24–1.11)(discontinuation due to toxic AEs)		
		CKD (eGFR < 45 mL/min/1.73 m2)	TNFi	89	JAKi	54	12M	Drug discontinuation due to AEs	93.1%	75.1%							**aHR (95% CI): 0.23 (0.09–0.61)(discontinuation due to toxic AEs)**		
			IL-6Ri	96	JAKi	54		Drug discontinuation due to AEs	94.1%	75.1%							**aHR (95% CI): 0.34 (0.14–0.81)(discontinuation due to toxic AEs)**		
			Abatacept	92	JAKi	54		Drug discontinuation due to AEs	92.3%	75.1%							**aHR (95% CI): 0.36 (0.15–0.89)(discontinuation due to toxic AEs)**		
		CKD (eGFR 45–60 mL/min/1.73 m2)	JAKi (TOF, BAR, or FIL) with dose reduction	67	JAKi (TOF, BAR, or FIL) without dose reduction	17	12M	Retention rate due to toxic AEs	93.3%	93.6%								Among patients on TOF, BAR, or FIL, dose reduction made no difference in those with moderate CKD, but in severe CKD, reduced doses were associated with lower AE-related retention than standard doses (although the diference was not statistically signifcant).	
		CKD (eGFR < 45 mL/min/1.73 m2)	JAKi (TOF, BAR, or FIL) with dose reduction	31	JAKi (TOF, BAR, or FIL) without dose reduction	5		Retention rate due to toxic AEs	80.5%	51.1%							aHR (95% CI) 0.15 (0.016–1.38)(discontinuation due to toxic AEs)		
Yoshimura 2024	Observational cohort study	CKD eGFR<30 mL/min/1.73 m2	IL-6Ri	14	TNFis	50	36M	ineffectiveness leading to discontinuation of medication	2 (14.3%)	26 (52%)						p=0.03	aHR (95% CI): 0.11 (0.02 to 0.85),	IL6-Ris in patients with an eGFR of <30mL/min/1.73 m2, was associated with a lower risk of bDMARD discontinuation due to ineffectiveness than that for TNFαis.	Moderate
			IL-6Ri	14	TNFis	50		infection leading to discontinuation of medication	1 (7.1%)	4 (8%)						p=0.69	aHR (95% CI) 0.64 (0.07 to 5.78)		
			Abatacept	6	TNFis	50		infection leading to discontinuation of medication	0 (0)	4 (8%)									
Yoshimura 2025	Observational cohort study	CKD (eGFR 30–60 mL/min/1.73 m2)	JAKi	100			within 24M	Discontinuation due to infection	5 (5%)									Herpes zoster and DVT occurred more frequently in those with eGFR <30, these differences were not statistically significant. Also, patients showed significant improvements with decreased DAS28-CRP and reduced prednisolone use over 6 months after JAKi initiation.	High
								Discontinuation due to adverse effect	19 (19%)										
								Herpes zoster	8 (8%)										
								DVT	4 (4%)										
								CVD	1 (1%)										
								Death	1 (1%)										
		CKD (eGFR < 30 mL/min/1.73 m2)	JAKi	19			within 24M	Discontinuation due to infection	1 (5.26%)										
								Discontinuation due to adverse effect	2 (10.52%)										
								Herpes zoster	3 (15.79%)								OR (95% CI) vs eGFR ≥60: 1.43 (0.68–3.02)		
								DVT	2 (10.52%)								OR (95% CI) vs eGFR ≥60: 1.50 (95% CI, 0.59–3.80)		
								CVD	0										
								Death	1 (5.26%)										
**Anaemia**																			
Specker 2022	Observational cohort study	Anaemia	Tocilizumab (men)	141	No comparator		Follow-up until 104W	ΔDAS28-ESR			-2.4 (2.1)	p = 0.14							High
			Tocilizumab (women)	437							-2.3 (1.7)	p = 0.07							
**Malignancies and Cancer**																			
**Studies regarding efficacy**	-	-	-	-	-	-	-	-	-	-	-	-	-	-	-	-	-	-	-
Kunishita 2023	Observational cohort study	Previous malignancy	Abatacept	73	No comparator		Baseline	DAS28-CRP			3.8 (1.2)							Abatacept was effective in patients with a history of malignancy	High
								SDAI			23.5 (13.1)								
							6 M	DAS28-CRP			2.2 (1.3)								
								SDAI			8.7 (11.6)								
							12 M	DAS28-CRP			1.9 (1.2)								
								SDAI			6.5 (11.1)								
							60 M	DAS28-CRP			1.3 (0.4)								
								SDAI			1.9 (2.5)								
**Studies regarding Safety**	-	-	-	-	-	-	-	-	-	-	-	-	-	-	-	-	-	-	-
pundole 2020	Observational cohort study	Solid Malignancies (excluding NMSC)	TNFi (etanercept, adalimumab, infliximab, certolizumab, golimumab)	NR	no bDMARDs	NR		Overall survival									aHR (95% CI): 0.67 (0.31–1.44)	No significant survival differences were found between RA patients with solid malignancies, comparing those on TNFi (etanercept, adalimumab,	High

			nonTNFi (abatacept, rituximab or tocilizumab)	NR	no bDMARDs	NR		Overall survival									aHR (95% CI): 1.10 (0.26–4.60)	infliximab, certolizumab, golimumab) or nonTNFi (abatacept, rituximab, tocilizumab) to those without bDMARDs.	
		Breast Cancer	TNFi (etanercept, adalimumab, infliximab, certolizumab, golimumab)	NR	no bDMARDs	NR		Overall survival									aHR (95% CI): 1.40 (0.42–4.73)	In breast cancer patients, TNFi users or nonTNFi users exhibited a higher but statistically nonsignificant HR compared with those who did not receive bDMARD.	
			nonTNFi (abatacept, rituximab or tocilizumab)	NR	no bDMARDs	NR		Overall survival									aHR (95% CI): 1.37 (0.22–8.42)		
Kunishita 2023	Observational cohort study	Previous malignancy	Abatacept	73	No comparator		60 M	infection leading to discontinuation	1 (4.8)									Abatacept was safe in patients with a history of malignancy	High
								Malignancy	2 (9.5)										
								Malignancy IR per 100,000 PY	779								aHR (95% CI): 0.994 (0.134–7.367) (previous malignancy vs no malignancy)		
Hoshida 2024	Observational cohort study	RA-associated Lymphoproliferative Disorder	Tacrolimus	71	NR		Median follow-up from the date of RA-LPD diagnosis: 44M	5-year overall survival rate	68 (96.4%)							p < 0.05 (TAC vs no drug)		TCZ-only regimens might be a better treatment option for patients with RA after LPD onset	Moderate
								Tumor Regrowth after Spontaneous Regression percent	29.50%										
								5-year Post-regression Growth-Free Survival Rate-percent	62.20%							**p < 0.01, (TCZ (95.0%) vs TAC (62.2%))**			
			Tocilizumab	37				5-year overall survival rate	34 (90.9%)							p < 0.05, (TCZ vs no drug)			
								Tumor Regrowth after Spontaneous Regression percent	7.70%										
								5-year Post-regression Growth-Free Survival Rate-percent	95%							**p < 0.01 (TCZ (95.0%) vs No drug (51.4%))**			
								Spontaneous Regression Retention									**aHR (95% CI) 0.106 (0.014–0.824) vs none**		
			MTX	26				5-year overall survival rate	24 (90.9%)										
								Tumor Regrowth after Spontaneous Regression percent	50%										
								5-year Post-regression Growth-Free Survival Rate-percent	33.30%							**p < 0.01, (TCZ (95.0%) vs MTX (33.3%))**			
			Abatacept	21				5-year overall survival rate	21 (100%)										
								Tumor Regrowth after Spontaneous Regression percent	11.80%										
								5-year Post-regression Growth-Free Survival Rate-percent	83.30%										
			Tocilizumab+tacrolimus	11				5-year overall survival rate	10 (91.8%)										
								Tumor Regrowth after Spontaneous Regression percent	57.10%										
								5-year Post-regression Growth-Free Survival Rate-percent	38.10%							**p < 0.01, (TCZ (95.0%) vs TAC plus TCZ (38.1%))**			
			TNFi	9				5-year overall survival rate	8 (91.5%)										
								Tumor Regrowth after Spontaneous Regression percent	42.90%										
								5-year Post-regression Growth-Free Survival Rate-percent	57.10%							p < 0.01, (TCZ (95.0%) vs TNFi (57.1%))			
			Rituximab	6				5-year overall survival rate	6 (100%)										
								Tumor Regrowth after Spontaneous Regression percent	0										
								5-year Post-regression Growth-Free Survival Rate-percent	100%										
Ruiz 2024	Observational cohort (Clinformatics® Data Mart Database)	Newly diagnosed Breast Cancer	TNFi	165	csDMARDs	669	12M	Overall Survival								p = 0.39	HR (95% CI): 0.77 (0.42–1.40)	TNFi treatment during the first two years after early Breast Cancer was not associated with worse survival. There was better Breast Cancer specific survival in those who received TNFis compared with csDMARDs only.	Moderate
	Observational cohort (SEER program)	Newly diagnosed Breast Cancer	TNFi	201	csDMARDs	848	12M	Overall Survival								p = 0.44	HR (95% CI): 0.84 (0.54–1.31)		
								Breast Cancer specific survival								**p = 0.046**	**HR (95% CI): 0.28 (0.08–0.98)**		
Molina-Collada 2024	Observational cohort study	History of malignancies	JAKi	51	TNFi	82	Mean follow-up: 21 M	Cancer IR (95% CI) (per 1000 PY) defined as any cancer (new primary, local recurrence or metastasis)	37.6 (12.1–116.6)	50.9 (27.4–94.6)							Adjusted Incidence Rate Ratio (95% CI): 0.7 (0.2–2.7	JAK inhibitors and newer biologics, do not show a significantly increased cancer risk in RA patients with prior malignancy compared to TNFi.	Moderate
								Mortality (per 1000 PY)	12.5	10.1							Adjusted Incidence Rate Ratio (95% CI): 0.6 (0.1–6.9)		

			Rituximab	46	TNFi	82		Cancer IR (95% CI) (per 1000 PY) defined as any cancer (new primary, local recurrence or metastasis)	15.1 (3.8–60.4)	50.9 (27.4–94.6)							Adjusted Incidence Rate Ratio (95% CI): 0.3 (0.1–1.3)		
								Mortality (per 1000 PY)	52.9	10.1							Adjusted Incidence Rate Ratio (95% CI): 3.2 (0.7–15.7)		
			IL-6Ri	53	TNFi	82		Cancer IR (95% CI) (per 1000 PY) defined as any cancer (new primary, local recurrence or metastasis)	83.9 (40.0–175.9)	50.9 (27.4–94.6)							Adjusted Incidence Rate Ratio (95% CI): 1.7 (0.6–4.3)		
								Mortality (per 1000 PY)	0	10.1									
			Abatacept	44	TNFi	82		Cancer IR (95% CI) (per 1000 PY) defined as any cancer (new primary, local recurrence or metastasis)	96.8 (50.4–186.1)	50.9 (27.4–94.6)							Adjusted Incidence Rate Ratio (95% CI): 1.9 (0.8–4.7)		
								Mortality (per 1000 PY)	75.3	10.1							Adjusted Incidence Rate Ratio (95% CI): 4.3 (0.9–21.4)		
Ruiz 2025	Observational cohort study	Recently diagnosed early stage colorectal cancer	TNFi	80	csDMARDs	324	1Y post cancer diagnosis (0–1y)	Overall survival								p = 0·22	aHR (95% CI): 0·72 (0·43–1·21)	In RA patients ≥66 years with early-stage colorectal, lung, or prostate cancer, no statistically significant harmful association was observed between TNF inhibitor use versus csDMARDs and overall or cancer-specific survival, and no dose effects were identified. In lung cancer, overall survival remained significantly improved at 3 years (aHR 0.58, 95% CI 0.34–0.96), while cancer-specific survival was not.	Moderate
								Colorectal cancer-specific survival								p = 0·27	aHR (95% CI): 1·91 (0·61–6·05)		
				67	csDMARDs	252	2Y post cancer diagnosis (0–2y)	Overall survival								p = 0·35	aHR (95% CI): 0·74 (0·40–1·38)		
								Colorectal cancer-specific survival								p = 0·84	aHR (95% CI): 0·85 (0·18–4·02)		
				56	csDMARDs	181	3Y post cancer diagnosis (0–3y)	Overall survival								p = 0·18	aHR (95% CI): 0·61 (0·30–1·25)		
								Colorectal cancer-specific survival								p = 0·29	aHR (95% CI):0·29 (0·03–2·82)		
		Recently diagnosed early stage lung cancer	TNFi	102	csDMARDs	604	1Y post cancer diagnosis (0–1y)	Overall survival								p = 0·050	aHR (95% CI): 0·70 (0·49–1·00)		
								Lung cancer-specific survival								p = 0·041	aHR (95% CI): 0·40 (0·17 – 0·96)		
				78	csDMARDs	395	2Y post cancer diagnosis (0–2y)	Overall survival								p = 0·053	aHR (95% CI): 0·66 (0·43–1·01)		
								Lung cancer-specific survival								p = 0·27	aHR (95% CI): 0·55 (0·19 – 1·60)		
				57	csDMARDs	269	3Y post cancer diagnosis (0–3y)	Overall survival								**p = 0·039**	**aHR (95% CI): 0·58 (0·34–0·96)**		
								Lung cancer-specific survival								p = 0·66	aHR (95% CI): 0·75 (0·21–2·65)		
		Recently diagnosed early stage prostate cancer	TNFi	120	csDMARDs	423	1Y post cancer diagnosis (0–1y)	Overall survival								p = 0·45	aHR (95% CI): 0·80 (0·44–1·44)		
				104	csDMARDs	336	2Y post cancer diagnosis (0–2y)	Overall survival								p = 0·64	aHR (95% CI): 0·86 (0·46–1·62)		
				84	csDMARDs	266	3Y post cancer diagnosis (0–3y)	Overall survival								p = 0·82	aHR (95% CI): 0·92 (0·47–1·83)		
Westermann 2025	Observational cohort study	Prior solid cancer	TNFi	81	csDMARD	651	TNFi: 343 PY; csDMARDs: 2750 PY	Cancer recurrence									aHR (95% CI): 1.10 (0.21 to 3.16)	No significant increase in recurrence risk was seen with TNFi or rituximab vs csDMARDs in RA patients with prior solid cancer in remission. Results for breast cancer recurrence, assessed separately, were also not statistically significant. However, limited power and wide CIs mean a clinically relevant risk cannot be ruled out.	Low
								Death									aHR (95% CI):1.38 (0.21 to 4.44)		
			Rituximab	99	csDMARD	651	RTX: 532 PY; csDMARDs: 2750 PY	Cancer recurrence									aHR (95% CI):0.94 (0.32 to 2.11)		
								Death									aHR (95% CI): 0.67 (0.13 to 1.51)		
		Prior breast cancer	TNFi	44	csDMARD	363	TNFi: 217 PY; csDMARDs: 1564 PY	Cancer recurrence									aHR (95% CI): 0.68 (0.00 to 2.37)		
			Rituximab	54	csDMARD	363	RTX: 285 PY; csDMARDs: 1564 PY	Cancer recurrence									aHR (95% CI): 0.52 (0.00 to 1.95)		
**Anti Drug Antibody positive**																			
Bitoun 2023	Subanalysis_RCT	Anti Drug Antibody positive	Infliximab and adalimumab	26			12M	EULAR Moderate and Good Response	9 (35%)							**p = 0.001**	OR (95% CI) (vs ADA negative patients): 0.11 (0.02-0.38)	Antidrug antibodies (ADAbs) significantly reduce response to anti-TNF monoclonal antibodies (adalimumab, infliximab), while their impact on etanercept and rituximab is not significant; for tocilizumab, there is a potential negative association, but the evidence is weaker (P = 0.07).	Moderate
			Etanercept	5			12M		3 (60%)							p = 0.29	OR (95% CI) (vs ADA negative patients): 0.35 (0.05-2.99)		
			Rituximab	15			12M		8 (53%)							p = 0.32	OR (95% CI) (vs ADA negative patients): 0.46 (0.09-2.08)		
			Tocilizumab	10			12M		7 (70%)							p = 0.07	OR (95% CI) (vs ADA negative patients): 0.11 (0.005-0.98)		
**High comorbidity burden**																			
pappas 2020	Observational cohort study	High comorbidity burden	Tocilizumab IV or SC	195	No comparator		6M	ΔCDAI			-7.72							Tocilizumab was effective regardless of comorbidity burden.	Moderate
								CDAI LDA (≤10)	21.5%								OR (95% CI)(high comorbidity burden vs low): 0.85 (0.45, 1.62)		

								ΔHAQ			-0.09								
								ΔCDAI			-7.82								
							12M	CDAI LDA (≤10)	31.3%								OR (95% CI)(high comorbidity burden vs low): 1.52 (0.85, 2.71)		
								ΔHAQ			-0.11								
**History of infection**																			
Cohen 2021	Pooled analysis of RCT data	Histoty of herpes zoster	Upadacitinib 15 mg QD in patients with history of herpes zoster	110	Upadacitinib 15 mg QD in patients without history of herpes zoster	2520		Herpes zoster									HR (95% CI: 24.194 (15.941, 36.719)	Among patients treated with upadacitinib 15 mg, those who had a history of HZ had a higher risk of HZ	High
Jeong 2022	Observational cohort study	History of herpes zoster	Etanercept	311	Abatacept	228	Median follow-up: 2.7 Y (ETN: 1004 PY, ABA: 394PY)	herpes zoster	71 (22.8)								aHR (95% CI): 1.22 (0.75–2.00), p=0.421	Among first-line b/tsDMARD users, an increased risk of recurrent herpes zoster was found only with tofacitinib in the adjusted analysis compared to abatacept and compared to etanercept. Among patients with recurrent herpes zoster who received tofacitinib, half of them developed herpes zoster 0.3 years after starting treatment (IQR: 0.1–0.8 years).	High
			Infliximab	186	Etanercept	311	Median follow-up: 2.7 Y (INF: 435 PY, ETN: 1004PY)	herpes zoster	35 (18.8)								aHR (95% CI): 0.88 (0.55–1.41), p=0.599		
					Abatacept	228	Median follow-up: 2.7 Y (INF: 435 PY, ABA: 394)										aHR (95% CI): 1.08 (0.62–1.87), p= 0.784		
			Adalimumab	412	Etanercept	311	Median follow-up: 2.7 Y (ADA: 1018PY, ETN: 1004PY)	herpes zoster	96 (23.3)								aHR (95% CI):1.22 (0.85–1.75), p=0.276		
					Abatacept	228	Median follow-up: 2.7 Y (ADA: 1018PY, ABA: 394PY)										aHR (95% CI):1.50 (0.93–2.40), p=0.094		
			Golimumab	190	Etanercept	311	Median follow-up: 2.7 Y (GOLI: 310 PY, ETN: 1004 PY)	herpes zoster	26 (13.7)								aHR (95% CI): 0.54 (0.27–1.09), p=0.084		
					Abatacept	228	Median follow-up: 2.7 Y (GOLI: 310 PY, ABA: 394 PY)										aHR (95% CI): 0.66 (0.32–1.37), p= 0.268		
			Tocilizumab	247	Etanercept	311	Median follow-up: 2.7 Y (TCZ: 392 PY, ETN: 1004 PY)	herpes zoster	34 (13.8)								aHR (95% CI):0.95 (0.57–1.58), p=0.838		
					Abatacept	228	Median follow-up: 2.7 Y (TCZ: 392 PY, ABA: 394 PY)										aHR (95% CI):1.16 (0.67–2.00), p=0.590		
			Tofacitinib	143	Etanercept	311	Median follow-up: 2.7 Y (TOFA: 97 PY, ETN: 1004 PY)	herpes zoster	18 (12.6)								**aHR (95% CI): 3.01 (1.49–6.11), p=0.002**		
					Abatacept	228	Median follow-up: 2.7 Y (TOFA: 97 PY, ABA: 394 PY)										**aHR (95% CI): 3.69 (1.77–7.69), p<0.001**		
			Abatacept	228	Etanercept	311	Median follow-up: 2.7 Y (ABA: 394PY, ETN: 1004 PY)	herpes zoster	33 (14.5)								aHR (95% CI): 0.82 (0.50–1.34), p=0.421		
Kuwana 2025	Observational cohort study (postmarketing)	History of infection	Tofacitinib 5 mg BID		Other DMARDs (MTX, csDMARDs, bDMARDs, or immunosuppressants)		Up to 1 Y	serious infection events (SIEs) IR per 100 PY	13.73	2.03									Moderate
**Mental Disorders**																			
Latocha 2023	RCT	Insomnia	Cognitive behavioural therapy for insomnia	31	Control group	31	6 M	Fatigue (BRAF-MDQ, 0–70)			24.0 (1.4)		36.4 (1.5)		-12.4 (-16.5, -8.4)	p <0.0001		Statistically significant differences between the groups in PtGA, pain (VAS), fatigue (BRAF-MDQ), and RA impact (RAID) at week 26, all in favor of CBT-I.	High
								RA impact of disease (RAID, 0–10)			4.2 (0.20)		5.5 (0.20)		-1.4 (-1.9, -0.80)	p <0.0001			
								Disease activity (DAS28-CRP)			3.3 (0.14)		3.5 (0.14)		-0.18 (-0.56, 0.21)	p=0.366			
								Tender joints (0–28)			5.4 (0.71)		5.5 (0.71)		-0.13 (-2.1, 1.9)	p=0.897			
								Swollen joints (0–28)			0.67 (0.22)		0.72 (0.23)		-0.05 (-0.69, 0.58)	p=0.868			
								CRP			3.8 (0.68)		3.20 (0.71)		0.63 (-1.3, 2.6)	p=0.525			
								Patient global assessment (VAS, 0–100)			41.0 (2.8)		54.0 (2.8)		-13.0 (-20.9, -5.1)	p=0.0014			
								Pain (VAS, 0–100)			38.9 (2.5)		48.4 (2.5)		-9.5 (-16.5, -2.5)	p=0.0085			
								HAQ			0.79 (0.04)		0.79 (0.04)		-0.01 (-0.13, 0.11)	p=0.887			
								SF-36-PCS (0–100)			39.1 (0.92)		36.7 (0.97)		2.3 (-0.31, 5.0)	p=0.082			
								SF-36-MCS (0–100)			46.2 (1.3)		49.4 (1.3)		3.3 (-0.34, 6.8)	p=0.075			
Citera 2024			Tofacitinib 5mg BID	870	Placebo	263	6M	ACR20 response rate (%)	61.70%										High

	Pooled analysis of RCT data	Probable major depressive disorder and/or probable generalized anxiety disorder (pMDD/pGAD) based on SF-36 (Short Form-36 Health Survey) ≤ 38						ACR50 response rate (%)	36.00%								
								ACR70 response rate (%)	16.50%								
								DAS28-4(ESR)-defined remission rate	5.90%								
								change from baseline in HAQ-DI, LS mean	-0.5								
								change from baseline in FACIT-F, LS mean	5.2								
								change from baseline in Pain (VAS), LS mean	-25.1								
							12M	ACR20 response rate (%)	58.40%								
								ACR50 response rate (%)	33.80%								
								ACR70 response rate (%)	18.30%								
								DAS28-4(ESR)-defined remission rate	8.00%								
								change from baseline in HAQ-DI, LS mean	-0.5								
								change from baseline in FACIT-F, LS mean	5.6								
								change from baseline in Pain (VAS), LS mean	-26.5								
			Tofacitinib 10mg BID	477	Adalimumab 40mg Q2W	264	6M	ACR20 response rate (%)	67.50%								
								ACR50 response rate (%)	42.90%								
								ACR70 response rate (%)	22.40%								
								DAS28-4(ESR)-defined remission rate	11.80%								
								change from baseline in HAQ-DI, LS mean	-0.6								
								change from baseline in FACIT-F, LS mean	5.8								
								change from baseline in Pain (VAS), LS mean	-28.3								
							12M	ACR20 response rate (%)	62.20%								
								ACR50 response rate (%)	37.30%								
								ACR70 response rate (%)	21.50%								
								DAS28-4(ESR)-defined remission rate	9.40%								
								change from baseline in HAQ-DI, LS mean	-0.6								
								change from baseline in FACIT-F, LS mean	6.3								
								change from baseline in Pain (VAS), LS mean	-29								

ACR, American College of Rheumatology; AE, adverse event; ALT, alanine aminotransferase; ASCVD, atherosclerotic cardiovascular disease; bDMARD, biologic disease-modifying antirheumatic drug; CDAI, Clinical Disease Activity Index; CKD, chronic kidney disease; CRP, C-reactive protein; csDMARD, conventional synthetic disease-modifying antirheumatic drug; DAS28, Disease Activity Score in 28 joints; DAS28-ESR, Disease Activity Score in 28 joints using erythrocyte sedimentation rate; DAS28-CRP, Disease Activity Score in 28 joints using C-reactive protein; DVT, deep vein thrombosis; EAER, exposure-adjusted event rate; EGA, evaluator global assessment; eGFR, estimated glomerular filtration rate; ERO, modified Sharp erosion score; ESR, erythrocyte sedimentation rate; EQ-5D-5L, EuroQol 5-Dimension 5-Level questionnaire; GC, glucocorticoid; HAQ, Health Assessment Questionnaire; HBV, hepatitis B virus; HBsAg, hepatitis B surface antigen; HBsAb, hepatitis B surface antibody; HBcAb, hepatitis B core antibody; IgG, immunoglobulin G; IL-6i, interleukin-6 inhibitor; IL-6Ri, interleukin-6 receptor inhibitor; IQR, interquartile range; IR, incidence rate; JAKi, Janus kinase inhibitor; JSN, joint space narrowing; LDA, low disease activity; LEF, leflunomide; M, months; MACE, major adverse cardiovascular event; MI, myocardial infarction; mHAQ, modified Health Assessment Questionnaire; mTSS, modified Total Sharp Score; MTX, methotrexate; NMSC, nonmelanoma skin cancer; NSIP, nonspecific interstitial pneumonia; OR, odds ratio; PLT, platelet; PtGA, patient global assessment; PY, patient-years; RA-ILD, rheumatoid arthritis–associated interstitial lung disease; RADAI-5, Rheumatoid Arthritis Disease Activity Index-5; RF, rheumatoid factor; ROR, reporting odds ratio; SC, subcutaneous; SDAI, Simplified Disease Activity Index; SJC28, swollen joint count in 28 joints; TEAE, treatment-emergent adverse event; TJC28, tender joint count in 28 joints; TNFi, tumor necrosis factor inhibitor; UIP, usual interstitial pneumonia; VAS, visual analogue scale; VTE, venous thromboembolism; W, weeks; WBC, white blood cell; Δ, change from baseline. aHR, adjusted hazard ratio; aIRR, adjusted incidence rate ratio; CI, confidence interval; HR, hazard ratio; IR, incidence rate; IRR, incidence rate ratio; OR, odds ratio; PY, patient-years; RR, relative risk; wHR, weighted hazard ratio. Higher-risk populations were defined as patients aged ≥50 years with ≥1 CV risk factor, based on ORAL Surveillance criteria and available data from the SELECT RA programme. Risk factors included: (1) prior CV event ('cardiac disorders' per MedDRA V.25.0), (2) hypertension (medical history only), (3) diabetes, (4) current/former tobacco use, and (5) HDL-C <40 mg/dL at baseline. Family history of premature coronary heart disease and extra-articular RA were not captured and thus excluded.
