# Supplementary material for: Therapeutic approaches for difficult-to-treat rheumatoid arthritis: a systematic literature review of current evidence
Source: RMD Open. 2026 Aug 4;12(3):e006860. doi: 10.1136/rmdopen-2026-006860 (PMC13448634; doi:10.1136/rmdopen-2026-006860)
Supplement: Supplementary file 2 [file rmdopen-12-3-s002.pdf]

# Therapeutic Approaches for Difficult-to-Treat Rheumatoid Arthritis: A Systematic Literature Review of Current Evidence

## Supplementary Appendix 2

# Contents

**1. Research Questions** ....................................................................4
**2. Population, Intervention, Comparator, Outcome (PICO) framework and eligibility criteria** ..........4
2.1 PICO 1 ....................................................................4
2.1.1 PICO 1 Details (Supplementary Table 2) ....................................................................4
2.1.2 Inclusion Criteria....................................................................6
2.1.3 Exclusion criteria ....................................................................6
2.2 PICO 2 ....................................................................7
2.2.1 PICO 2 Details (Supplementary Table 3) ....................................................................7
2.2.2 Inclusion Criteria....................................................................9
2.2.3 Exclusion Criteria ....................................................................9
2.3 PICO 3 ....................................................................10
2.3.1 PICO 3 Details (Supplementary Table 4) ....................................................................10
2.3.2 Inclusion Criteria....................................................................11
2.2.3 Exclusion Criteria ....................................................................11
**3. Comparison of PICO criteria between the 2020 SLR and the current SLR** (Supplementary Table 5) ....................................................................12
**4. Literature Search** ....................................................................13
4.1 Search period ....................................................................13
4.1.1 Original Search ....................................................................13
4.1.2 Search Update ....................................................................13
4.2 Search Strategy ....................................................................13
4.2.1 Ovid MEDLINE ....................................................................13
4.2.2 Embase....................................................................17
4.2.3 Cochrane Central Register of Controlled Trials ....................................................................22
4.2.4 Cochrane Database of Systematic Reviews ....................................................................24
**5. Overview of included studies** ....................................................................25
5.1 PICO 2 (efficacy) ....................................................................25
5.1.1 RCTs (Supplementary Table 6) ....................................................................25
5.1.2 Pooled analyses of RCT data (Supplementary Table 7) ....................................................................27
5.2 PICO 2 (safety)....................................................................28
5.2.1 Observational Studies (Supplementary Table 8) ....................................................................28
5.3 PICO 3 (Supplementary Table 9) ....................................................................30
**6. Risk of Bias Assessment** ....................................................................31
6.1 PICO 1 Studies ....................................................................31
6.1.1 Observational studies using NOS (Supplementary Table 10) ....................................................................31

6.1.2 RCTs using RoB 2.0 (Figure S1) ....................40
6.2 PICO 2 Studies ....................41
6.2.1 Observational studies using NOS (Supplementary Table 11) ....................41
6.2.2 RCTs using RoB 2.0 (Figure S2) ....................43
6.3 PICO 3 Studies ....................44
6.3.1 Observational Study using NOS (Supplementary Table 12) ....................44
6.3.2 RCTs using RoB 2.0 (Figure S3) ....................44
**7. Additional Results** ....................45
7.1 PICO2 safety outcomes (Supplementary Table 13) ....................45
7.2 Meta-regression results ....................48
7.2.1 RR of ACR20 Response (Supplementary Table 14) ....................48
7.2.1 Absolute ACR20 Response (Supplementary Table 15) ....................48
7.3. Operationalization of EULAR D2T RA components in Studies (Supplementary Table 16) ....................49
**References** ....................50

# 1. Research Questions

1. What therapeutic strategies are reported for patients with Rheumatoid arthritis (RA) with insufficiently controlled disease activity with contraindications/limitations (comorbidities, drug-drug interactions) to certain disease-modifying antirheumatic drugs (DMARDs)?

2. What therapeutic strategies are reported for patients who failed ≥2 biologic/ targeted synthetic (b/ts) DMARD and have active disease?

3. What therapeutic strategies are reported for patients with RA and predominantly non-inflammatory complaints?

# 2. Population, Intervention, Comparator, Outcome (PICO) framework and eligibility criteria

## 2.1 PICO 1

Efficacy and safety of treatments in patients with active RA but limited DMARD options due to comorbidities, contraindications, or drug–drug interactions

### 2.1.1 PICO 1 Details (Supplementary Table 2)

| | |
|---|---|
| Population | Rheumatoid arthritis & comorbidities or contraindications or (risk of) drug-drug interactions, and active disease (terms: D2T, difficult to treat, difficult-to-treat, DMARD failure, bDMARD failure) |
| Intervention | Any of the following interventions, alone or in combination:<br>csDMARD<br>bDMARD<br>tsDMARD<br>glucocorticoids, steroids,<br>Education: Formalised or protocolised education from the specialist nurse/ health care professional as part of the multidisciplinary team<br>Education, learning, counselling, one-to-one education, group education, health literacy and related terms (e.g., health liter*)<br>App-supported care |
| Comparator | Any (combination) of or none of the treatments/interventions/procedures listed under intervention<br>Continuation of treatment strategy (same/continuous glucocorticoid/DMARD regimen/dose/route/monotherapy)<br>Other/continuous DMARD/different dose of DMARD<br>No treatment, placebo, other non-pharmacological,<br>Standard care<br>No control, if DMARD is administered in a population with "contraindications" |
| Outcome | At least one of below listed outcome measures:<br>**Efficacy:**<br>ACR Response Criteria (ACR20, ACR50, ACR70) |

| | |
|---|---|
| | DAS28 (Disease Activity Score using 28 joint counts)<br>SDAI (Simplified Disease Activity Index)<br>CDAI (Clinical Disease Activity Index)<br>EULAR Response Criteria<br>Disease activity states by DAS28, SDAI, CDAI<br>ACR/EULAR Boolean Remission Criteria<br>Swollen Joint count 28/32/66<br>Tender Joint Count 28/32/68<br>HAQ-DI (Health Assessment Questionnaire Disability Index)<br>mHAQ (Modified Health Assessment Questionnaire)<br>Sharp/van der Heijde Score<br>Larsen Score<br>Patient Global Assessment (PGA)<br>Pain Scale<br>Fatigue Assessment (VAS/NRS; FACIT)<br>RAPID3 (Routine Assessment of Patient Index Data 3)<br>SF-36 (Short Form-36 Health Survey)<br>EQ-5D (EuroQol-5 Dimensions)<br>C-reactive protein (CRP)/erythrocyte sedimentation rate (ESR)<br>**Safety:**<br>Overall safety outcomes:<br>• Adverse events (AE)<br>• Serious adverse events (SAE)<br>• Adverse events leading to treatment discontinuation<br>• All-cause mortality<br>• Treatment-related mortality<br>Infections:<br>• Serious infections<br>• Opportunistic infections (including tuberculosis and fungal infections)<br>Malignancies:<br>• Malignancies (including new or recurrent cancers, lymphomas, and skin cancers)<br>Thromboembolic events:<br>• Venous thromboembolism (deep vein thrombosis, pulmonary embolism)<br>Cardiovascular outcomes<br>• Major adverse cardiovascular events (MACE)<br>• Myocardial infarction<br>• Stroke<br>• Hypertension<br>Lipid levels<br>Hematologic abnormalities:<br>• Anemia<br>• Leukopenia<br>• Neutropenia<br>• Thrombocytopenia<br>• Changes in leukocyte counts<br>• Complete blood count (CBC) abnormalities<br>Hepatic function: |

| | |
|---|---|
| | • Hepatotoxicity:<br>Elevated transaminases (ALT, AST), bilirubin, and other liver function abnormalities<br>Renal function:<br>• Renal function impairment:<br>Serum creatinine<br>Blood urea nitrogen (BUN)<br>Gastrointestinal events:<br>• Gastrointestinal bleeding<br>• Gastrointestinal perforations<br>• Gastrointestinal ulcerations<br>Pulmonary events:<br>• Pneumonitis<br>• Interstitial lung disease<br>• Exacerbation of pre-existing lung disease<br>Other safety outcomes:<br>• Drug–drug interactions<br>• Injection-site reactions<br>• Hypersensitivity reactions (including anaphylaxis)<br>• Immunogenicity (anti-drug antibodies)<br>Corticosteroid-related adverse outcomes:<br>Bone mineral density loss<br>Cataracts<br>Hyperglycemia |

### 2.1.2 Inclusion Criteria

- Adult RA patients (≥18 years)
- Diagnosed according to 2010 ACR/EULAR criteria for RA or 1984 ARA criteria (in observational studies also other measures verifying the diagnosis could be applicable).
- Randomized controlled trials (RCTs), post-hoc analyses of RCTs, pooled analyses of RCT data (individual participant data pooling), cohorts (including single arm trials/studies), case-controls, long-term extension studies of RCTs.

### 2.1.3 Exclusion criteria

- DMARD naïve patient populations
    - Studies including DMARD-naïve patients were excluded unless a clearly defined eligible subgroup without DMARD-naïve patients was reported.
- Patient populations exclusively in remission or with low disease activity.
- Pregnancy-related studies
- Studies evaluating none of the interventions of interest.
- Outcomes not defined or not relevant to the predefined outcomes of interest.
- Sample size < 40.
- Editorials, comments, case reports, systematic literature reviews, conference abstracts.
- For post-hoc studies and pooled analyses of RCT data, only the most recent publications were included.
- Studies published in language other than English.

## 2.2 PICO 2

Efficacy and safety of treatments in patients with persistent disease activity after failure of ≥2 prior b/tsDMARDs.

### 2.2.1 PICO 2 Details (Supplementary Table 3)

| | |
|---|---|
| Population | Rheumatoid arthritis, pretreated with ≥2b/tsDMARDs, and active disease (terms: Biologically refractory, D2T, difficult to treat, difficult-to-treat, true refractory) |
| Intervention | Any of the following interventions, alone or in combination:<br>DMARD, including non-standard dosing and combination treatments<br>csDMARD, bDMARDs, tsDMARDs<br>Education: Formalised or protocolised education from the specialist nurse/ health care professional as part of the multidisciplinary team<br>Education, learning, counselling, one-to-one education, group education, health literacy and related terms (e.g. health liter*)<br>App-supported care |
| Comparator | Continuation of background therapy without modification (same glucocorticoid or DMARD, dose, route of administration, and treatment composition);<br>Another DMARD/different dose of a DMARD; or<br>Placebo |
| Outcome | Any (combination) of below list of outcomes:<br>Efficacy outcomes:<br>ACR Response Criteria (ACR20, ACR50, ACR70)<br>DAS28 (Disease Activity Score using 28 joint counts)<br>SDAI (Simplified Disease Activity Index)<br>CDAI (Clinical Disease Activity Index)<br>EULAR Response Criteria<br>Disease activity states by DAS28, SDAI, CDAI<br>ACR/EULAR Boolean Remission Criteria<br>Swollen Joint count 28/32/66<br>Tender Joint Count 28/32/68<br>HAQ-DI (Health Assessment Questionnaire Disability Index)<br>mHAQ (Modified Health Assessment Questionnaire)<br>Sharp/van der Heijde Score<br>Larsen Score<br>Patient Global Assessment (PGA)<br>Pain Scale<br>Fatigue Assessment (VAS/NRS; FACIT)<br>RAPID3 (Routine Assessment of Patient Index Data 3)<br>SF-36 (Short Form-36 Health Survey)<br>EQ-5D (EuroQol-5 Dimensions)<br>C-reactive protein (CRP)/erythrocyte sedimentation rate (ESR)<br><br>**Safety:**<br><br>Overall safety outcomes:<br>• Adverse events (AE)<br>• Serious adverse events (SAE)<br>• Adverse events leading to treatment discontinuation<br>• All-cause mortality |

| | |
|---|---|
| | • Treatment-related mortality<br>Infections:<br>• Serious infections<br>• Opportunistic infections (including tuberculosis and fungal infections)<br>Malignancies:<br>• Malignancies (including new or recurrent cancers, lymphomas, and skin cancers)<br>Thromboembolic events:<br>• Venous thromboembolism (deep vein thrombosis, pulmonary embolism)<br>Cardiovascular outcomes<br>• Major adverse cardiovascular events (MACE)<br>• Myocardial infarction<br>• Stroke<br>• Hypertension<br>Lipid levels<br>Hematologic abnormalities:<br>• Anemia<br>• Leukopenia<br>• Neutropenia<br>• Thrombocytopenia<br>• Changes in leukocyte counts<br>• Complete blood count (CBC) abnormalities<br>Hepatic function:<br>• Hepatotoxicity:<br>Elevated transaminases (ALT, AST), bilirubin, and other liver function abnormalities<br>Renal function:<br>• Renal function impairment:<br>Serum creatinine<br>Blood urea nitrogen (BUN)<br>Gastrointestinal events:<br>• Gastrointestinal bleeding<br>• Gastrointestinal perforations<br>• Gastrointestinal ulcerations<br>Pulmonary events:<br>• Pneumonitis<br>• Interstitial lung disease<br>• Exacerbation of pre-existing lung disease<br>Other safety outcomes:<br>• Drug–drug interactions<br>• Injection-site reactions<br>• Hypersensitivity reactions (including anaphylaxis)<br>• Immunogenicity (anti-drug antibodies)<br>Corticosteroid-related adverse outcomes:<br>Bone mineral density loss<br>Cataracts<br>Hyperglycemia |

### 2.2.2 Inclusion Criteria

- Adult RA patients (≥18 years).
- Diagnosed according to 2010 ACR/EULAR criteria for RA or 1984 ARA criteria (in observational studies also other measures verifying the diagnosis could be applicable).
- Failure of ≥2 b/tsDMARDs, due to lack of efficacy and/or intolerance, not necessarily with different mechanism of action, with ongoing active disease.
- For PICO 2 (efficacy): RCTs, post-hoc analyses of RCTs, pooled analyses of RCT data (individual participant data pooling).
- For PICO 2 (safety): RCTs, post-hoc analyses of RCTs, pooled analyses of RCT data, observational cohort studies, case-controls, long-term extension studies of RCTs.

### 2.2.3 Exclusion Criteria

- Patient populations exclusively in remission or with low disease activity.
- Studies evaluating none of the interventions of interest.
- Studies without a comparator group (e.g., single-arm studies).
- Outcomes not defined or not relevant to the predefined outcomes of interest.
- Sample size < 40.
- Editorials, comments, case reports, systematic literature reviews, conference abstracts.
- For post-hoc studies and pooled analyses of RCT data, only the most recent publications were included.
- Studies published in language other than English.

## 2.3 PICO 3

Efficacy of non-pharmacological interventions in RA patients with poor health-related quality of life (HRQoL) and low objective markers of disease activity.

### 2.3.1 PICO 3 Details (Supplementary Table 4)

| | |
|---|---|
| Population | Rheumatoid arthritis, received at least one current or prior b/tsDMARD |
| Intervention | Any of the following interventions, alone or in combination:<br>• Non-DMARD treatments, including non-pharmacological interventions (e.g., self-efficacy interventions, cognitive behavioural therapy, psychotherapy, lifestyle interventions)<br>• Education, including formalised or protocolised education delivered by specialist nurses or other healthcare professionals as part of a multidisciplinary team<br>• Education and counselling interventions, including one-to-one education, group education, learning interventions, and health literacy–related interventions<br>• App-supported care |
| Comparator | • Any of the treatments, interventions, or procedures listed above<br>• Usual care or continuation of care (including the same glucocorticoid and/or DMARD regimen, dose, and route of administration)<br>• Alternative DMARD strategies, including initiation of a new DMARD or a different DMARD dose<br>• Placebo |
| Outcome | Any of the following outcomes, alone or in combination:<br><br>Disease activity and treatment effectiveness, measured using validated indices and objective measures of inflammation:<br><br>• ACR Response Criteria (ACR20, ACR50, ACR70)<br>• DAS28 (Disease Activity Score using 28 joint counts)<br>• SDAI (Simplified Disease Activity Index)<br>• CDAI (Clinical Disease Activity Index)<br>• EULAR Response Criteria<br>• Disease activity states based on DAS28, SDAI, or CDAI<br>• ACR/EULAR Boolean Remission Criteria<br>• Swollen joint count (28/32/66)<br>• Tender joint count (28/32/68)<br><br>**Quality of life (QoL) and patient-reported outcome measures (PROMs):**<br><br>• Goal Attainment Scaling (GAS)<br>• HAQ-DI (Health Assessment Questionnaire Disability Index)<br>• mHAQ (Modified Health Assessment Questionnaire)<br>• Patient Global Assessment (PGA)<br>• Pain scales<br>• Fatigue assessments (VAS/NRS; FACIT)<br>• RAPID3 (Routine Assessment of Patient Index Data 3) |

| | |
|---|---|
| | • SF-36 (Short Form-36 Health Survey)<br>• EQ-5D (EuroQol-5 Dimensions)<br>• Arthritis Self-Efficacy Scale (ASES)<br>• Self-Efficacy for Managing Chronic Disease (6-item scale)<br>• PEPPI-5 (Perceived Efficacy in Patient–Physician Interactions)<br>• Patient Acceptable Symptom State (PASS)<br>• Work productivity (IPCQ)<br>• GR-eHealth Literacy Scale<br>• Shared decision-making process survey |

### 2.3.2 Inclusion Criteria

- Adult RA patients (≥18 years).
- Diagnosed according to 2010 ACR/EULAR criteria for RA or 1984 ARA criteria (in observational studies also other measures verifying the diagnosis could be applicable).

### 2.2.3 Exclusion Criteria

- Patient populations exclusively with high disease activity.
- Studies evaluating pharmacological interventions.
- Studies without a comparator group (e.g., single-arm studies).
- Outcomes not defined or not relevant to the predefined outcomes of interest.
- Sample size < 30.
- Editorials, comments, case reports, systematic literature reviews, conference abstracts.
- Studies published in language other than English.

## 3. Comparison of PICO criteria between the 2020 SLR and the current SLR (Supplementary Table 5)

Supplementary Table 5. Comparison of PICO and key eligibility criteria between the 2020 SLR and the current SLR

| | 2020 SLR[1] | Current SLR | Key Differences |
|---|---|---|---|
| **PICO 1** (PICO 1.a in 2020 SLR) | What are effective management options when DMARD choices are limited, because of adverse events, comorbidity or contraindications, to treat persistent inflammation in D2T RA?<br><br>P: D2T RA patients with inflammatory activity and limited DMARD options; I: Management options (including non-pharmacological therapy); C: No or different management options; O: Efficacy and/or safety/tolerability | What are the efficacy and safety of treatments in patients with active RA but limited treatment options due to comorbidities, contraindications, or drug–drug interactions?<br><br>(PICO details specified in Supplementary Table 2) | In the current SLR, DMARD-naïve populations explicitly excluded; sample size ≥40 introduced; conference abstracts excluded; pregnancy-related studies excluded |
| PICO 2 (PICO 1.b in 2020 SLR) | What are effective management options for true refractory RA?<br><br>P: D2T RA with inflammation not explained by other causes (true refractory RA); I: Management options (including non-pharmacological therapy); C: No or different management options; O: Reduction of RA inflammation | What is the efficacy and safety of treatments in patients with persistent disease activity after failure of ≥2 prior b/tsDMARDs?<br><br>(PICO details specified in Supplementary Table 3) | In the current SLR, threshold raised from ≥1 to ≥2 b/tsDMARD failures, directly aligning with EULAR D2T RA definition; sample size ≥40 introduced; observational studies not eligible for efficacy outcomes; comparator group required |
| PICO 3 (PICO 1.c in 2020 SLR) | What are effective management options for RA patients with persistent complaints that are not explained by active inflammation?<br><br>P: D2T RA patients with persistent complaints not explained by active inflammation; I: Management options (including non-pharmacological therapy); C: No or different management options; O: Reduction in (specific) complaints | What is the efficacy of non-pharmacological interventions in RA patients with poor health-related quality of life (HRQoL) and low objective markers of disease activity?<br><br>(PICO details specified in Supplementary Table 4) | The current SLR requires objective evidence of low inflammation; prior or current b/tsDMARD use required; sample size ≥30 introduced; comparator group required; pharmacological interventions explicitly excluded |

b/tsDMARD, biological/targeted synthetic disease-modifying antirheumatic drug; CDAI, Clinical Disease Activity Index; csDMARD, conventional synthetic disease-modifying antirheumatic drug; D2T RA, difficult-to-treat rheumatoid arthritis; DMARD, disease-modifying antirheumatic drug; HRQoL, health-related quality of life; PICO, Population, Intervention, Comparator, Outcome; RA, rheumatoid arthritis; SLR, systematic literature review.

# 4. Literature Search

## 4.1 Search period

### 4.1.1 Original Search

01 December 2019 to 12 September 2024

### 4.1.2 Search Update

12 September 2024 to 9 July 2025

## 4.2 Search Strategy

### 4.2.1 Ovid MEDLINE

| | |
|---|---|
| 1 | (d2tra or "d2t ra").ti,ab,kf. |
| 2 | ((ra or "rheumat* arthriti*" or "rheumat* arthropath*" or "rheumat* polyarthriti*") adj5 ("difficult to treat*" or d2t)).ti,ab,kf. |
| 3 | ((ra or "rheumat* arthriti*" or "rheumat* arthropath*" or "rheumat* polyarthriti*") adj3 (established or refractory or fail* or resistan* or unsuccess* or retreat* or pretreat* or intoleran* or inadequat*)).ti,ab,kf. |
| 4 | ((ra or "rheumat* arthriti*" or "rheumat* arthropath*" or "rheumat* polyarthriti*") adj7 ((previous* or subsequent* or discontinu* or switch* or "for more than" or cycling or second*) adj3 (treat* or therap* or medicat*))).ti,ab,kf. |
| 5 | ((ra or "rheumat* arthriti*" or "rheumat* arthropath*" or "rheumat* polyarthriti*") adj7 ((adverse or side) adj2 (effect* or event* or react*))).ti,ab,kf. |
| 6 | ((ra or "rheumat* arthriti*" or "rheumat* arthropath*" or "rheumat* polyarthriti*") adj7 (adverse adj2 drug*)).ti,ab,kf. |
| 7 | (exp *Arthritis, Rheumatoid/ or ("rheumat* arthriti*" or "rheumat* arthropath*" or "rheumat* polyarthriti*").ti,kf.) and ("difficult to treat*" or d2t).ti,ab,kf. |
| 8 | (exp *Arthritis, Rheumatoid/ or ("rheumat* arthriti*" or "rheumat* arthropath*" or "rheumat* polyarthriti*").ti,kf.) and (established adj2 (disease* or arthriti* or arthropath* or polyarthriti*)).ti,ab,kf. |
| 9 | (exp *Arthritis, Rheumatoid/ or ("rheumat* arthriti*" or "rheumat* arthropath*" or "rheumat* polyarthriti*").ti,kf.) and ((refractory or fail* or resistan* or unsuccess* or retreat* or pretreat* or intoleran* or inadequat*).ti,ab,kf. or (previous* or subsequent* or discontinu* or switch* or 'for more than' or cycling or second*).ti,kf.) |
| 10 | (exp *Arthritis, Rheumatoid/ or ("rheumat* arthriti*" or "rheumat* arthropath*" or "rheumat* polyarthriti*").ti,kf.) and ((previous* or subsequent* or discontinu* or switch* or 'for more than' or cycling or second*) adj3 (treat* or therap* or medicat*)).ti,ab,kf. |
| 11 | (exp *Arthritis, Rheumatoid/ or ("rheumat* arthriti*" or "rheumat* arthropath*" or "rheumat* polyarthriti*").ti,kf.) and ((adverse or side) adj2 (effect* or event* or react*)).ti,ab,kf. |
| 12 | (exp *Arthritis, Rheumatoid/ or ("rheumat* arthriti*" or "rheumat* arthropath*" or "rheumat* polyarthriti*").ti,kf.) and ((adverse or interact*) adj2 drug*).ti,ab,kf. |
| 13 | (exp *Arthritis, Rheumatoid/ or ("rheumat* arthriti*" or "rheumat* arthropath*" or "rheumat* polyarthriti*").ti,kf.) and exp Drug Resistance/ |

| | |
|---|---|
| 14 | (exp *Arthritis, Rheumatoid/ or ("rheumat* arthriti*" or "rheumat* arthropath*" or "rheumat* polyarthriti*").ti,kf.) and (exp "Drug-Related Side Effects and Adverse Reactions"/ or exp Long Term Adverse Effects/ or exp Drug Interactions/) |
| 15 | (exp *Arthritis, Rheumatoid/ or ("rheumat* arthriti*" or "rheumat* arthropath*" or "rheumat* polyarthriti*").ti,kf.) and adverse effects.fs. |
| 16 | (exp *Arthritis, Rheumatoid/ or ("rheumat* arthriti*" or "rheumat* arthropath*" or "rheumat* polyarthriti*").ti,kf.) and (exp *Obesity/ or (obes* or adipos* or overweight*).ti,kf. or exp *body mass index/ or ("body mass index" or bmi).ti,kf. or exp *Depression/ or (depression* or depressive*).ti,kf. or exp *comorbidity/ or (comorbid* or "co morbid*" or multimorbid* or "multi morbid*").ti,kf. or exp *contraindications/ or (contraindicat* or "contra indicat*").ti,kf. or complication*.ti,kf. or exp *infections/ or infect*.ti,kf. or exp *hepatitis/ or hepatitis*.ti,kf. or exp *hiv/ or (hiv* or aids* or "human immunodeficiency virus*" or "human immune deficiency virus*" or "acquired immunodeficiency*" or "acquired immune deficiency*").ti,kf. or exp *tuberculosis/ or tuberculo*.ti,kf. or exp *neoplasms/ or (neoplas* or malignan* or tumor* or tumour* or cancer*).ti,kf. or exp *lung diseases/ or exp *bronchial diseases/ or ("lung disease*" or "bronchial disease*" or asthma* or copd* or "chronic obstructive pulmonary disease*" or "chronic obstructive lung disease*" or "chronic airflow obstruction*" or "chronic obstructive airway disease*" or bronchitis or "pulmonary fibros*" or "fibrosing alveoliti*").ti,kf. or exp *cardiovascular diseases/ or ("cardiovascular disease*" or "cv disease*" or cvd or "cardiac event*").ti,kf. or (hypertension* or "high blood pressure*").ti,kf. or exp *hyperlipidemias/ or (hyperlipidemia* or hyperlipidaemia* or hyperlipemia*).ti,kf. or (cardiomyopath* or "cardiac failure*" or "myocardial disease*" or myocardiopath*).ti,kf. or exp *kidney diseases/ or ((kidney or renal) adj (disease* or failure* or dysfunction* or insufficienc*)).ti,kf. or exp *liver diseases/ or (liver adj (failure* or dysfunction* or insufficienc* or disease* or cirrhos* or "enzyme elevation*")).ti,kf. or exp *aspartate aminotransferases/ or (alat or alt or "aspartate transaminase*" or "aspartate aminotransferase*" or "serum glutamic oxaloacetic transaminase*" or sgot or "glutamic oxaloacetic transaminase*" or "l aspartate 2 oxoglutarate aminotransferase*" or "glutamate aspartate transaminase*" or asat or ast).ti,kf. or exp alanine transaminase/ or ("alanine transaminase*" or "alanine aminotransferase*" or "glutamic pyruvic transaminase*" or "alanine 2 oxoglutarate aminotransferase*" or sgpt).ti,kf. or exp *osteoporosis/ or (osteoporos* or "bone loss*").ti,kf. or osteopenia*.ti,kf. or exp *diabetes mellitus/ or diabet*.ti,kf. or exp *pregnancy/ or pregnan*.ti,kf. or *stomach ulcer/ or ("stomach ulcer*" or "ulcus ventriculi" or "ulcus duodeni" or "gastric ulcer*").ti,kf. or exp *fatigue/ or *fatigue syndrome, chronic/ or fatigue*.ti,kf. or (thrombos* or "blood clot*").ti,kf. or exp *thrombophilia/ or (thrombophilia* or hypercoagulabilit*).ti,kf.) and (("disease activit*" or remission* or "drug survival*" or persistenc* or (success* adj1 therap*) or response* or (effective* or efficac*) or toxic* or safety or tolerab* or ("acr response" or acr20 or acr50 or acr70 or "acr 20" or "acr 50" or "acr 70" or das28 or "das 28" or ("disease activity score" adj3 "28*") or sdai or "simplified disease activity index" or cdai or "clinical disease activity index" or "eular response" or "acr/eular" or "eular remission" or "swollen joint count*" or "tender joint count*" or "haq di" or haqdi or "health assessment questionnaire disability index" or mhaq or "m haq" or "modified health assessment questionnaire" or "sharp/van der heijde score" or "larsen score" or "patient global assessment" or (pain* adj3 (scor* or scal*)) or "fatigue assessment" or facit or "vas/nrs" or rapid3 or "sf 36" or sf36 or "short form 36 health survey" or "eq 5d" or eq5d or "euroqol 5*" or "euroqol5*" or "crp/esr" or "goal attainment scaling" or "arthritis self efficacy scale" or "self efficacy for managing chronic disease*" or peppi* or "patient acceptability symptom* state*" or "patient work productivity" or ipcq or "gr ehealth literacy scale*" or "shared decision making processing survey*")).ti,ab,kf. or exp "quality of life"/ or "quality of life".ti,ab,kf. or "life quality".ti,ab,kf. or hrqol.ti,ab,kf. or qol.ti,ab,kf. or exp patient reported outcome measures/ or (prom or proms or (promis* adj3 (survey* or score*)) or promis or prem or prems).ti,ab,kf. or (exp diagnostic self evaluation/ and (outcome* or experience* or measur* or assess* or questionnaire* or survey* or score* or scale* or tool* or inventor* or |

instrument*).ti,ab,kf.) or (("self assess*" or selfassess* or "patient* assess*" or patientassess*) adj3 (outcome* or experience* or measur* or questionnaire* or survey* or score* or scale* or tool* or inventor* or instrument*)).ti,ab,kf. or (("patient* report*" or patientreport* or "self report*" or selfreport* or "participant* report*" or participantreport* or "subject* report*" or subjectreport* or "patient* centered*" or patientcentered* or "self evaluat*" or selfevaluat* or "self monitor*" or selfmonitor* or "patient* evaluat*" or patientevaluat* or "parent* report*" or parentreport* or "caregive* report*" or caregiverreport*) adj3 (outcome* or experience* or measur* or assess* or questionnaire* or survey* or score* or scale* or tool* or inventor* or instrument*)).ti,ab,kf. or exp treatment outcome/ or outcome*.ti,ab,kf. or (goal* adj2 (setting* or manag* or treatment*)).ti,ab,kf. or (self adj2 (care* or manag* or efficacy*)).ti,ab,kf. or exp self concept/ or exp self care/ or exp self-management/) [mp=title, book title, abstract, original title, name of substance word, subject heading word, floating sub-heading word, keyword heading word, organism supplementary concept word, protocol supplementary concept word, rare disease supplementary concept word, unique identifier, synonyms, population supplementary concept word, anatomy supplementary concept word]

17 (exp *Arthritis, Rheumatoid/ or ("rheumat* arthriti*" or "rheumat* arthropath*" or "rheumat* polyarthriti*").ti,kf.) and ("health-related quality of life" or hrqol).ti,kf.

18 (exp *Arthritis, Rheumatoid/ or ("rheumat* arthriti*" or "rheumat* arthropath*" or "rheumat* polyarthriti*").ti,kf.) and (("quality of life" or "life quality" or hrqol or qol) adj5 (low* or poor* or weak* or declin* or reduct* or decreas* or few or less or wors* or impair*)).ti,ab,kf.

19 1 or 2 or 3 or 4 or 5 or 6 or 7 or 8 or 9 or 10 or 11 or 12 or 13 or 14 or 15 or 16 or 17 or 18

20 exp case-control studies/

21 exp Cohort Studies/

22 (case* adj3 (control* or referent* or comparison or base) adj3 (study or studies or analysis*)).ti,ab,kf.

23 (cohort* or retrospective* or prospective* or longitudinal* or follow-up* or followup*).ti,ab,kf.

24 ("case series" adj3 (study or studies or analysis*)).ti,ab,kf.

25 "case series".ti. or exp clinical trial, phase iv/ or ("clinical trial*" adj3 ("phase iv" or "phase 4")).ti,ab,kf.

26 exp randomized controlled trial/

27 controlled clinical trial.pt.

28 (randomized or randomised).ab.

29 placebo.ab.

30 drug therapy.fs.

31 randomly.ab.

32 trial.ab.

33 groups.ab.

34 20 or 21 or 22 or 23 or 24 or 25 or 26 or 27 or 28 or 29 or 30 or 31 or 32 or 33

35 exp animals/ not humans.sh.

36 34 not 35

37 19 and 36

38 limit 37 to dt=20191201-20240912

39 38 not (comment or editorial or letter or review or case-reports or published-erratum or guideline or practice-guideline).pt.

### 4.2.2 Embase

#72 #70 OR #71

#71 #69 AND 'clinical trial'/it AND [2019-2025]/py

#70 #67 NOT ('conference abstract'/it OR 'editorial'/it OR 'letter'/it OR 'note'/it OR 'review'/it OR 'short survey'/it OR erratum/it OR chapter/it OR 'case report'/de OR 'clinical trial'/it) AND [01-12-2019]/sd NOT [13-09-2024]/sd

#69 #67 NOT ('conference abstract'/it OR 'editorial'/it OR 'letter'/it OR 'note'/it OR 'review'/it OR 'short survey'/it OR erratum/it OR chapter/it OR 'case report'/de) AND [01-12-2019]/sd NOT [13-09-2024]/sd

#68 #67 NOT ('conference abstract'/it OR 'editorial'/it OR 'letter'/it OR 'note'/it OR 'review'/it OR 'short survey'/it OR erratum/it OR chapter/it OR 'case report'/de)

#67 #24 AND #66

#66 #65 NOT ('animal experiment'/de NOT ('human experiment'/de OR 'human'/de))

#65 #25 OR #26 OR #27 OR #28 OR #29 OR #30 OR #64

#64 #50 NOT #63

#63 #51 OR #52 OR #53 OR #54 OR #55 OR #56 OR #57 OR #58 OR #59 OR #60 OR #61 OR #62

#62 (rat:ti,tt OR rats:ti,tt OR mouse:ti,tt OR mice:ti,tt OR swine:ti,tt OR porcine:ti,tt OR murine:ti,tt OR sheep:ti,tt OR lambs:ti,tt OR pigs:ti,tt OR piglets:ti,tt OR rabbit:ti,tt OR rabbits:ti,tt OR cat:ti,tt OR cats:ti,tt OR dog:ti,tt OR dogs:ti,tt OR cattle:ti,tt OR bovine:ti,tt OR monkey:ti,tt OR monkeys:ti,tt OR trout:ti,tt OR marmoset*:ti,tt) AND 'animal experiment'/de

#61 (databases NEAR/5 searched):ab

#60 'update review':ab

#59 'we searched':ab AND (review:ti,tt OR review:it)

#58 review:ab AND review:it NOT trial:ti,tt

#57 ('random cluster' NEAR/4 sampl*):ti,ab,tt

#56 'random field*':ti,ab,tt

#55 nonrandom*:ti,ab,tt NOT random*:ti,ab,tt

#54 'systematic review':ti,tt NOT (trial:ti,tt OR study:ti,tt)

#53 'case control*':ti,ab,tt AND random*:ti,ab,tt NOT ('randomised controlled':ti,ab,tt OR 'randomized controlled':ti,ab,tt)

#52 'cross-sectional study'/de NOT ('randomized controlled trial'/exp OR 'controlled clinical study'/de OR 'controlled study'/de OR 'randomised controlled':ti,ab,tt OR 'randomized controlled':ti,ab,tt OR 'control group':ti,ab,tt OR 'control groups':ti,ab,tt)

#51 ((random* NEXT/1 sampl* NEAR/8 ('cross section*' OR questionnaire* OR survey OR surveys OR database OR databases)):ti,ab,tt) NOT ('comparative study'/de OR 'controlled study'/de OR 'randomised controlled':ti,ab,tt OR 'randomized controlled':ti,ab,tt OR 'randomly assigned':ti,ab,tt)

#50 #31 OR #32 OR #33 OR #34 OR #35 OR #36 OR #37 OR #38 OR #39 OR #40 OR #41 OR #42 OR #43 OR #44 OR #45 OR #46 OR #47 OR #48 OR #49

#49 trial:ti,tt

#48 'human experiment'/de

#47 volunteer:ti,ab,tt OR volunteers:ti,ab,tt

#46 (controlled NEAR/7 (study OR design OR trial)):ti,ab,tt

#45 assigned:ti,ab,tt OR allocated:ti,ab,tt

#44 ((assign* OR match OR matched OR allocation) NEAR/5 (alternate OR group OR groups OR intervention OR interventions OR patient OR patients OR subject OR subjects OR participant OR participants)):ti,ab,tt

#43 crossover:ti,ab,tt OR 'cross over':ti,ab,tt

#42 (parallel NEXT/1 group*):ti,ab,tt

#41 'double blind procedure'/de

#40 ((double OR single OR doubly OR singly) NEXT/1 (blind OR blinded OR blindly)):ti,ab,tt

#39 (open NEXT/1 label):ti,ab,tt

#38 (evaluated:ab OR evaluate:ab OR evaluating:ab OR assessed:ab OR assess:ab) AND (compare:ab OR compared:ab OR comparing:ab OR comparison:ab)

#37 compare:ti,tt OR compared:ti,tt OR comparison:ti,tt

#36 placebo:ti,ab,tt

#35 'intermethod comparison'/de

#34 'randomization'/de

#33 random*:ti,ab,tt

#32 'controlled clinical trial'/de

#31 'randomized controlled trial'/exp

#30 'case series':ti OR 'phase 4 clinical trial'/exp OR (('clinical trial*' NEAR/3 ('phase iv' OR 'phase 4')):ti,ab,kw)

#29 ('case series' NEAR/3 (study OR studies OR analysis*)):ti,ab,kw

#28 cohort*:ti,ab,kw OR retrospective*:ti,ab,kw OR prospective*:ti,ab,kw OR longitudinal*:ti,ab,kw OR 'follow up*':ti,ab,kw OR followup*:ti,ab,kw

#27 (case* NEAR/3 (control* OR referent* OR comparison OR base) NEAR/3 (study OR studies OR analysis*)):ti,ab,kw

#26 'cohort analysis'/exp

#25 'case control study'/exp

#24 #1 OR #2 OR #3 OR #6 OR #9 OR #12 OR #13 OR #14 OR #15 OR #16 OR #17 OR #18 OR #19 OR #20 OR #21 OR #22 OR #23

#23 ('rheumatoid arthritis'/exp/mj OR 'rheumat* arthriti*':ti,kw OR 'rheumat* arthropath*':ti,kw OR 'rheumat* polyarthriti*':ti,kw) AND ((('quality of life' OR 'life quality' OR hrqol OR qol) NEAR/5 (low* OR poor* OR weak* OR declin* OR reduct* OR decreas* OR few OR less OR wors* OR impair*)):ti,ab,kw)

#22 ('rheumatoid arthritis'/exp/mj OR 'rheumat* arthriti*':ti,kw OR 'rheumat* arthropath*':ti,kw OR 'rheumat* polyarthriti*':ti,kw) AND ('health-related quality of life':ti,kw OR hrqol:ti,kw)

#21 ('rheumatoid arthritis'/exp/mj OR 'rheumat* arthriti*':ti,kw OR 'rheumat* arthropath*':ti,kw OR 'rheumat* polyarthriti*':ti,kw) AND ('obesity'/exp/mj OR obes*:ti,kw OR adipos*:ti,kw OR overweight*:ti,kw OR 'body mass'/exp/mj OR 'body mass index':ti,kw OR bmi:ti,kw OR 'depression'/exp/mj OR depression*:ti,kw OR depressive*:ti,kw OR 'comorbidity'/exp/mj OR 'multiple chronic conditions'/exp/mj OR comorbid*:ti,kw OR 'co morbid*':ti,kw OR multimorbid*:ti,kw OR 'multi morbid*':ti,kw OR 'contraindication'/exp/mj OR contraindicat*:ti,kw OR 'contra indicat*':ti,kw OR complication*:ti,kw OR 'infection'/exp/mj OR infect*:ti,kw OR 'hepatitis'/exp/mj OR hepatitis*:ti,kw OR 'human immunodeficiency virus'/exp/mj OR hiv*:ti,kw OR aids*:ti,kw OR 'human immunodeficiency virus*':ti,kw OR 'human immune deficiency virus*':ti,kw OR 'acquired immunodeficiency*':ti,kw OR 'acquired immune deficiency*':ti,kw OR 'tuberculosis'/exp/mj OR tuberculo*:ti,kw OR 'neoplasm'/exp/mj OR neoplas*:ti,kw OR malignan*:ti,kw OR tumor*:ti,kw OR tumour*:ti,kw OR cancer*:ti,kw OR 'lung disease'/exp/mj OR 'bronchus disease'/exp/mj OR 'lung disease*':ti,kw OR 'bronchial disease*':ti,kw OR asthma*:ti,kw OR copd*:ti,kw OR 'chronic obstructive pulmonary disease*':ti,kw OR 'chronic obstructive lung disease*':ti,kw OR 'chronic airflow obstruction*':ti,kw OR 'chronic obstructive airway disease*':ti,kw OR bronchitis:ti,kw OR 'pulmonary fibros*':ti,kw OR 'fibrosing alveoliti*':ti,kw OR 'cardiovascular disease'/exp/mj OR 'cardiovascular disease*':ti,kw OR 'cv disease*':ti,kw OR cvd:ti,kw OR 'cardiac event*':ti,kw OR hypertension*:ti,kw OR 'high blood pressure*':ti,kw OR 'hyperlipidemia'/exp/mj OR hyperlipidemia*:ti,kw OR hyperlipidaemia*:ti,kw OR hyperlipemia*:ti,kw OR cardiomyopath*:ti,kw OR 'cardiac failure*':ti,kw OR 'myocardial disease*':ti,kw OR myocardiopath*:ti,kw OR 'kidney disease'/exp/mj OR (((kidney OR renal) NEXT/1 (disease* OR failure* OR dysfunction* OR insufficienc*)):ti,kw) OR 'liver disease'/exp/mj OR ((liver NEXT/1 (failure* OR dysfunction* OR insufficienc* OR disease* OR cirrhos* OR 'enzyme elevation*')):ti,kw) OR 'aspartate aminotransferase'/de OR alat:ti,kw OR alt:ti,kw OR 'aspartate transaminase*':ti,kw OR 'aspartate aminotransferase*':ti,kw OR 'serum glutamic oxaloacetic transaminase*':ti,kw OR sgot:ti,kw OR 'glutamic oxaloacetic transaminase*':ti,kw OR 'l aspartate 2 oxoglutarate aminotransferase*':ti,kw OR 'glutamate aspartate transaminase*':ti,kw OR asat:ti,kw OR ast:ti,kw OR 'alanine aminotransferase'/de OR 'alanine transaminase*':ti,kw OR 'alanine aminotransferase*':ti,kw OR 'glutamic pyruvic transaminase*':ti,kw OR 'alanine 2 oxoglutarate aminotransferase*':ti,kw OR sgpt:ti,kw OR 'osteoporosis'/exp/mj OR osteoporos*:ti,kw OR 'bone loss*':ti,kw OR 'osteopenia'/exp/mj OR osteopenia*:ti,kw OR 'diabetes mellitus'/exp/mj OR diabet*:ti,kw OR 'pregnancy'/exp/mj OR pregnan*:ti,kw OR 'stomach ulcer'/exp/mj OR 'stomach ulcer*':ti,kw OR 'ulcus ventriculi':ti,kw OR 'ulcus duodeni':ti,kw OR 'gastric ulcer*':ti,kw OR 'fatigue'/exp/mj OR fatigue*:ti,kw OR thrombos*:ti,kw OR 'blood clot*':ti,kw OR 'thrombophilia'/exp/mj OR thrombophilia*:ti,kw OR hypercoagulabilit*:ti,kw) AND ('disease activity'/exp OR 'disease activit*':ti,ab,kw OR remission*:ti,ab,kw OR 'drug survival*':ti,ab,kw OR persistenc*:ti,ab,kw OR ((success* NEAR/1 therap*):ti,ab,kw) OR response*:ti,ab,kw OR effective*:ti,ab,kw OR efficac*:ti,ab,kw OR toxic*:ti,ab,kw OR safety:ti,ab,kw OR tolerab*:ti,ab,kw OR 'acr response':ti,ab,kw OR acr20:ti,ab,kw OR acr50:ti,ab,kw OR acr70:ti,ab,kw OR 'acr 20':ti,ab,kw OR 'acr 50':ti,ab,kw OR 'acr 70':ti,ab,kw OR das28:ti,ab,kw OR 'das 28':ti,ab,kw OR (('disease activity score' NEAR/3 '28*'):ti,ab,kw) OR sdai:ti,ab,kw OR 'simplified disease activity index':ti,ab,kw OR cdai:ti,ab,kw OR 'clinical disease activity index':ti,ab,kw OR 'eular response':ti,ab,kw OR 'acr/eular':ti,ab,kw OR 'eular remission':ti,ab,kw OR 'swollen joint count*':ti,ab,kw OR 'tender joint count*':ti,ab,kw OR 'haq di':ti,ab,kw OR haqdi:ti,ab,kw OR 'health assessment questionnaire disability index':ti,ab,kw OR mhaq:ti,ab,kw OR 'm haq':ti,ab,kw OR 'modified health assessment questionnaire':ti,ab,kw OR 'sharp/van der heijde score':ti,ab,kw OR 'larsen score':ti,ab,kw OR 'patient global assessment':ti,ab,kw OR ((pain* NEAR/3 (scor* OR scal*)):ti,ab,kw) OR 'fatigue assessment':ti,ab,kw OR facit:ti,ab,kw OR 'vas/nrs':ti,ab,kw OR rapid3:ti,ab,kw OR 'sf 36':ti,ab,kw OR sf36:ti,ab,kw OR 'short form 36 health survey':ti,ab,kw OR 'eq 5d':ti,ab,kw OR eq5d:ti,ab,kw OR 'euroqol 5*':ti,ab,kw OR 'euroqol5*':ti,ab,kw OR 'crp/esr':ti,ab,kw OR 'goal attainment

scaling':ti,ab,kw OR 'arthritis self efficacy scale':ti,ab,kw OR 'self efficacy for managing chronic disease*':ti,ab,kw OR peppi*:ti,ab,kw OR 'patient acceptability symptom* state*':ti,ab,kw OR 'patient work productivity':ti,ab,kw OR ipcq:ti,ab,kw OR 'gr ehealth literacy scale*':ti,ab,kw OR 'shared decision making processing survey*':ti,ab,kw OR 'quality of life'/exp OR 'quality of life':ti,ab,kw OR 'life quality':ti,ab,kw OR hrqol:ti,ab,kw OR qol:ti,ab,kw OR 'patient-reported outcome'/exp OR prom:ti,ab,kw OR proms:ti,ab,kw OR ((promis* NEAR/3 (survey* OR score*)):ti,ab,kw) OR promis:ti,ab,kw OR prem:ti,ab,kw OR prems:ti,ab,kw OR ('self evaluation'/exp AND (outcome*:ti,ab,kw OR experience*:ti,ab,kw OR measur*:ti,ab,kw OR assess*:ti,ab,kw OR questionnaire*:ti,ab,kw OR survey*:ti,ab,kw OR score*:ti,ab,kw OR scale*:ti,ab,kw OR tool*:ti,ab,kw OR inventor*:ti,ab,kw OR instrument*:ti,ab,kw)) OR ((('self assess*' OR selfassess* OR 'patient* assess*' OR patientassess*) NEAR/3 (outcome* OR experience* OR measur* OR questionnaire* OR survey* OR score* OR scale* OR tool* OR inventor* OR instrument*)):ti,ab,kw) OR ((('patient* report*' OR patientreport* OR 'self report*' OR selfreport* OR 'participant* report*' OR participantreport* OR 'subject* report*' OR subjectreport* OR 'patient* centered*' OR patientcentered* OR 'self evaluat*' OR selfevaluat* OR 'self monitor*' OR selfmonitor* OR 'patient* evaluat*' OR patientevaluat* OR 'parent* report*' OR parentreport* OR 'caregive* report*' OR caregiverreport*) NEAR/3 (outcome* OR experience* OR measur* OR assess* OR questionnaire* OR survey* OR score* OR scale* OR tool* OR inventor* OR instrument*)):ti,ab,kw) OR 'treatment outcome'/exp OR outcome*:ti,ab,kw OR ((goal* NEAR/2 (setting* OR manag* OR treatment*)):ti,ab,kw) OR ((self NEAR/2 (care* OR manag* OR efficacy*)):ti,ab,kw) OR 'self care'/exp OR 'self concept'/exp)

#20 ('rheumatoid arthritis'/exp/mj OR 'rheumat* arthriti*':ti,kw OR 'rheumat* arthropath*':ti,kw OR 'rheumat* polyarthriti*':ti,kw) AND ('adverse drug reaction'/lnk OR 'side effect'/lnk)

#19 ('rheumatoid arthritis'/exp/mj OR 'rheumat* arthriti*':ti,kw OR 'rheumat* arthropath*':ti,kw OR 'rheumat* polyarthriti*':ti,kw) AND ('drug resistance'/exp OR 'adverse drug reaction'/exp OR 'adverse event'/exp OR 'side effect'/exp OR 'drug interaction'/exp)

#18 ('rheumatoid arthritis'/exp/mj OR 'rheumat* arthriti*':ti,kw OR 'rheumat* arthropath*':ti,kw OR 'rheumat* polyarthriti*':ti,kw) AND ((adverse NEAR/2 drug*):ti,ab,kw)

#17 ('rheumatoid arthritis'/exp/mj OR 'rheumat* arthriti*':ti,kw OR 'rheumat* arthropath*':ti,kw OR 'rheumat* polyarthriti*':ti,kw) AND (((adverse OR side) NEAR/2 (effect* OR event* OR react*)):ti,ab,kw)

#16 ('rheumatoid arthritis'/exp/mj OR 'rheumat* arthriti*':ti,kw OR 'rheumat* arthropath*':ti,kw OR 'rheumat* polyarthriti*':ti,kw) AND (((previous* OR subsequent* OR discontinu* OR switch* OR 'for more than' OR cycling OR second*) NEAR/3 (treat* OR therap* OR medicat*)):ti,ab,kw)

#15 ('rheumatoid arthritis'/exp/mj OR 'rheumat* arthriti*':ti,kw OR 'rheumat* arthropath*':ti,kw OR 'rheumat* polyarthriti*':ti,kw) AND (refractory:ti,ab,kw OR fail*:ti,ab,kw OR resistan*:ti,ab,kw OR unsuccess*:ti,ab,kw OR retreat*:ti,ab,kw OR pretreat*:ti,ab,kw OR intoleran*:ti,ab,kw OR inadequat*:ti,ab,kw OR previous*:ti,kw OR subsequent*:ti,kw OR discontinu*:ti,kw OR switch*:ti,kw OR 'for more than':ti,kw OR cycling:ti,kw OR second*:ti,kw)

#14 ('rheumatoid arthritis'/exp/mj OR 'rheumat* arthriti*':ti,kw OR 'rheumat* arthropath*':ti,kw OR 'rheumat* polyarthriti*':ti,kw) AND ((established NEAR/2 (disease* OR arthriti* OR arthropath* OR polyarthriti*)):ti,ab,kw)

#13 ('rheumatoid arthritis'/exp/mj OR 'rheumat* arthriti*':ti,kw OR 'rheumat* arthropath*':ti,kw OR 'rheumat* polyarthriti*':ti,kw) AND ('difficult to treat*':ti,ab,kw OR d2t:ti,ab,kw)

#12 #10 AND #11

#11 ((adverse OR interact*) NEAR/2 drug*):ti,ab,kw

#10 ((ra OR 'ra patient*' OR 'rheumat* arthriti*' OR 'rheumat* arthropath*' OR 'rheumat* polyarthriti*') NEAR/7 adverse):ti,ab,kw

#9 #7 AND #8

#8 ((adverse OR side) NEAR/2 (effect* OR event* OR react*)):ti,ab,kw

#7 ((ra OR 'ra patient*' OR 'rheumat* arthriti*' OR 'rheumat* arthropath*' OR 'rheumat* polyarthriti*') NEAR/7 (adverse OR side)):ti,ab,kw

#6 #4 AND #5

#5 ((previous* OR subsequent* OR discontinu* OR switch* OR 'for more than' OR cycling OR second*) NEAR/3 (treat* OR therap* OR medicat*)):ti,ab,kw

#4 ((ra OR 'ra patient*' OR 'rheumat* arthriti*' OR 'rheumat* arthropath*' OR 'rheumat* polyarthriti*') NEAR/7 (previous* OR subsequent* OR discontinu* OR switch* OR 'for more than' OR cycling OR second*)):ti,ab,kw

#3 ((ra OR 'ra patient*' OR 'rheumat* arthriti*' OR 'rheumat* arthropath*' OR 'rheumat* polyarthriti*') NEAR/3 (established OR refractory OR fail* OR resistan* OR unsuccess* OR retreat* OR pretreat* OR intoleran* OR inadequat*)):ti,ab,kw

#2 ((ra OR 'ra patient*' OR 'rheumat* arthriti*' OR 'rheumat* arthropath*' OR 'rheumat* polyarthriti*') NEAR/5 ('difficult to treat*' OR d2t)):ti,ab,kw

#1 d2tra:ti,ab,kw OR 'd2t ra':ti,ab,kw

### 4.2.3 Cochrane Central Register of Controlled Trials

1 (d2tra or "d2t ra").ti,ab,kw.

2 ((ra or "rheumat* arthriti*" or "rheumat* arthropath*" or "rheumat* polyarthriti*") adj5 ("difficult to treat*" or d2t)).ti,ab,kw.

3 ((ra or "rheumat* arthriti*" or "rheumat* arthropath*" or "rheumat* polyarthriti*") adj3 (established or refractory or fail* or resistan* or unsuccess* or retreat* or pretreat* or intoleran* or inadequat*)).ti,ab,kw.

4 ((ra or "rheumat* arthriti*" or "rheumat* arthropath*" or "rheumat* polyarthriti*") adj7 ((previous* or subsequent* or discontinu* or switch* or "for more than" or cycling or second*) adj3 (treat* or therap* or medicat*))).ti,ab,kw.

5 ((ra or "rheumat* arthriti*" or "rheumat* arthropath*" or "rheumat* polyarthriti*") adj7 ((adverse or side) adj2 (effect* or event* or react*))).ti,ab,kw.

6 ((ra or "rheumat* arthriti*" or "rheumat* arthropath*" or "rheumat* polyarthriti*") adj7 (adverse adj2 drug*)).ti,ab,kw.

7 (exp *Arthritis, Rheumatoid/ or ("rheumat* arthriti*" or "rheumat* arthropath*" or "rheumat* polyarthriti*").ti,kw.) and ("difficult to treat*" or d2t).ti,ab,kw.

8 (exp *Arthritis, Rheumatoid/ or ("rheumat* arthriti*" or "rheumat* arthropath*" or "rheumat* polyarthriti*").ti,kw.) and (established adj2 (disease* or arthriti* or arthropath* or polyarthriti*)).ti,ab,kw.

9 (exp *Arthritis, Rheumatoid/ or ("rheumat* arthriti*" or "rheumat* arthropath*" or "rheumat* polyarthriti*").ti,kw.) and ((refractory or fail* or resistan* or unsuccess* or retreat* or pretreat* or intoleran* or inadequat*).ti,ab,kw. or (previous* or subsequent* or discontinu* or switch* or 'for more than' or cycling or second*).ti,kw.)

10 (exp *Arthritis, Rheumatoid/ or ("rheumat* arthriti*" or "rheumat* arthropath*" or "rheumat* polyarthriti*").ti,kw.) and ((previous* or subsequent* or discontinu* or switch* or 'for more than' or cycling or second*) adj3 (treat* or therap* or medicat*)).ti,ab,kw.

11 (exp *Arthritis, Rheumatoid/ or ("rheumat* arthriti*" or "rheumat* arthropath*" or "rheumat* polyarthriti*").ti,kw.) and ((adverse or side) adj2 (effect* or event* or react*)).ti,ab,kw.

12 (exp *Arthritis, Rheumatoid/ or ("rheumat* arthriti*" or "rheumat* arthropath*" or "rheumat* polyarthriti*").ti,kw.) and ((adverse or interact*) adj2 drug*).ti,ab,kw.

13 (exp *Arthritis, Rheumatoid/ or ("rheumat* arthriti*" or "rheumat* arthropath*" or "rheumat* polyarthriti*").ti,kw.) and exp Drug Resistance/

14 (exp *Arthritis, Rheumatoid/ or ("rheumat* arthriti*" or "rheumat* arthropath*" or "rheumat* polyarthriti*").ti,kw.) and (exp "Drug-Related Side Effects and Adverse Reactions"/ or exp Long Term Adverse Effects/ or exp Drug Interactions/)

15 (exp *Arthritis, Rheumatoid/ or ("rheumat* arthriti*" or "rheumat* arthropath*" or "rheumat* polyarthriti*").ti,kw.) and adverse effects.fs.

16 (exp *Arthritis, Rheumatoid/ or ("rheumat* arthriti*" or "rheumat* arthropath*" or "rheumat* polyarthriti*").ti,kw.) and (exp *Obesity/ or (obes* or adipos* or overweight*).ti,kw. or exp *body mass index/ or ("body mass index" or bmi).ti,kw. or exp *Depression/ or (depression* or depressive*).ti,kw. or exp *comorbidity/ or (comorbid* or "co morbid*" or multimorbid* or "multi

morbid*").ti,kw. or exp *contraindications/ or (contraindicat* or "contra indicat*").ti,kw. or complication*.ti,kw. or exp *infections/ or infect*.ti,kw. or exp *hepatitis/ or hepatitis*.ti,kw. or exp *hiv/ or (hiv* or aids* or "human immunodeficiency virus*" or "human immune deficiency virus*" or "acquired immunodeficiency*" or "acquired immune deficiency*").ti,kw. or exp *tuberculosis/ or tuberculo*.ti,kw. or exp *neoplasms/ or (neoplas* or malignan* or tumor* or tumour* or cancer*).ti,kw. or exp *lung diseases/ or exp *bronchial diseases/ or ("lung disease*" or "bronchial disease*" or asthma* or copd* or "chronic obstructive pulmonary disease*" or "chronic obstructive lung disease*" or "chronic airflow obstruction*" or "chronic obstructive airway disease*" or bronchitis or "pulmonary fibros*" or "fibrosing alveoliti*").ti,kw. or exp *cardiovascular diseases/ or ("cardiovascular disease*" or "cv disease*" or cvd or "cardiac event*").ti,kw. or (hypertension* or "high blood pressure*").ti,kw. or exp *hyperlipidemias/ or (hyperlipidemia* or hyperlipidaemia* or hyperlipemia*).ti,kw. or (cardiomyopath* or "cardiac failure*" or "myocardial disease*" or myocardiopath*).ti,kw. or exp *kidney diseases/ or ((kidney or renal) adj (disease* or failure* or dysfunction* or insufficienc*)).ti,kw. or exp *liver diseases/ or (liver adj (failure* or dysfunction* or insufficienc* or disease* or cirrhos* or "enzyme elevation*")).ti,kw. or exp *aspartate aminotransferases/ or (alat or alt or "aspartate transaminase*" or "aspartate aminotransferase*" or "serum glutamic oxaloacetic transaminase*" or sgot or "glutamic oxaloacetic transaminase*" or "l aspartate 2 oxoglutarate aminotransferase*" or "glutamate aspartate transaminase*" or asat or ast).ti,kw. or exp alanine transaminase/ or ("alanine transaminase*" or "alanine aminotransferase*" or "glutamic pyruvic transaminase*" or "alanine 2 oxoglutarate aminotransferase*" or sgpt).ti,kw. or exp *osteoporosis/ or (osteoporos* or "bone loss*").ti,kw. or osteopenia*.ti,kw. or exp *diabetes mellitus/ or diabet*.ti,kw. or exp *pregnancy/ or pregnan*.ti,kw. or *stomach ulcer/ or ("stomach ulcer*" or "ulcus ventriculi" or "ulcus duodeni" or "gastric ulcer*").ti,kw. or exp *fatigue/ or *fatigue syndrome, chronic/ or fatigue*.ti,kw. or (thrombos* or "blood clot*").ti,kw. or exp *thrombophilia/ or (thrombophilia* or hypercoagulabilit*).ti,kw.) and (("disease activit*" or remission* or "drug survival*" or persistenc* or (success* adj1 therap*) or response* or (effective* or efficac*) or toxic* or safety or tolerab* or ("acr response" or acr20 or acr50 or acr70 or "acr 20" or "acr 50" or "acr 70" or das28 or "das 28" or ("disease activity score" adj3 "28*") or sdai or "simplified disease activity index" or cdai or "clinical disease activity index" or "eular response" or "acr/eular" or "eular remission" or "swollen joint count*" or "tender joint count*" or "haq di" or haqdi or "health assessment questionnaire disability index" or mhaq or "m haq" or "modified health assessment questionnaire" or "sharp/van der heijde score" or "larsen score" or "patient global assessment" or (pain* adj3 (scor* or scal*)) or "fatigue assessment" or facit or "vas/nrs" or rapid3 or "sf 36" or sf36 or "short form 36 health survey" or "eq 5d" or eq5d or "euroqol 5*" or "euroqol5*" or "crp/esr" or "goal attainment scaling" or "arthritis self efficacy scale" or "self efficacy for managing chronic disease*" or peppi* or "patient acceptability symptom* state*" or "patient work productivity" or ipcq or "gr ehealth literacy scale*" or "shared decision making processing survey*")).ti,ab,kw. or exp "quality of life"/ or "quality of life".ti,ab,kw. or "life quality".ti,ab,kw. or hrqol.ti,ab,kw. or qol.ti,ab,kw. or exp patient reported outcome measures/ or (prom or proms or (promis* adj3 (survey* or score*)) or promis or prem or prems).ti,ab,kw. or (exp diagnostic self evaluation/ and (outcome* or experience* or measur* or assess* or questionnaire* or survey* or score* or scale* or tool* or inventor* or instrument*).ti,ab,kw.) or (("self assess*" or selfassess* or "patient* assess*" or patientassess*) adj3 (outcome* or experience* or measur* or questionnaire* or survey* or score* or scale* or tool* or inventor* or instrument*)).ti,ab,kw. or (("patient* report*" or patientreport* or "self report*" or selfreport* or "participant* report*" or participantreport* or "subject* report*" or subjectreport* or "patient* centered*" or patientcentered* or "self evaluat*" or selfevaluat* or "self monitor*" or selfmonitor* or "patient* evaluat*" or patientevaluat* or "parent* report*" or parentreport* or "caregive* report*" or caregiverreport*) adj3 (outcome* or experience* or

measur* or assess* or questionnaire* or survey* or score* or scale* or tool* or inventor* or instrument*)).ti,ab,kw. or exp treatment outcome/ or outcome*.ti,ab,kw. or (goal* adj2 (setting* or manag* or treatment*)).ti,ab,kw. or (self adj2 (care* or manag* or efficacy*)).ti,ab,kw. or exp self concept/ or exp self care/ or exp self-management/)

17 (exp *Arthritis, Rheumatoid/ or ("rheumat* arthriti*" or "rheumat* arthropath*" or "rheumat* polyarthriti*").ti,kw.) and ("health-related quality of life" or hrqol).ti,kw.

18 (exp *Arthritis, Rheumatoid/ or ("rheumat* arthriti*" or "rheumat* arthropath*" or "rheumat* polyarthriti*").ti,kw.) and (("quality of life" or "life quality" or hrqol or qol) adj5 (low* or poor* or weak* or declin* or reduct* or decreas* or few or less or wors* or impair*)).ti,ab,kw.

19 1 or 2 or 3 or 4 or 5 or 6 or 7 or 8 or 9 or 10 or 11 or 12 or 13 or 14 or 15 or 16 or 17 or 18

20 limit 19 to (clinical trial protocol or comment or conference proceeding or editorial or erratum or letter or multimedia or note or trial registry record)

21 19 not 20

22 21 and ("2019 Issue 12" or "2020*" or "2021*" or "2022*" or "2023*" or "2024*").il.

### 4.2.4 Cochrane Database of Systematic Reviews

1 (rheumat* adj3 (arthriti* or arthropath* or polyarthriti*)).ti,ab,kw.

2 limit 1 to full systematic reviews

# 5. Overview of included studies

## 5.1 PICO 2 (efficacy)

### 5.1.1 RCTs (Supplementary Table 6)

Supplementary Table 6. Study and baseline characteristics of RCTs reporting efficacy outcomes in active RA patients with ≥2 prior b/tsDMARDs

| Study ID | Trial name | Phase | Population | Treatment | Target | N | Age (Year) | Female (Percent) | Disease duration (Year) | DAS28CRP | DAS28ESR | CDAI | SDAI |
|---|---|---|---|---|---|---|---|---|---|---|---|---|---|
| Fleischmann 2023 [2] | contRAst 2 | 3 | Active RA; csDMARD-IR; bDMARD-IR allowed; no prior JAKi or anti–GM-CSF/GM-CSFR | otilimab 90mg SC QW + csDMARDs | GM-CSF | 545 | 55.2 | 79% | 10.14 | 5.7 | | 38.3 | |
| | | | | otilimab 150mg SC QW + csDMARDs | GM-CSF | 539 | 55.6 | 80% | 9.97 | 5.7 | | 39 | |
| | | | | tofacitinib 5mg PO BID + csDMARDs | JAK1/3 | 271 | 55.4 | 85% | 10.58 | 5.9 | | 40.1 | |
| | | | | placebo + csDMARDs | | 270 | 55.5 | 79% | 9.95 | 5.7 | | 38.5 | |
| Taylor 2023 [3] | contRAst 3 | 3 | Active RA; csDMARD-IR; bDMARD-IR and/or JAKi-IR; no prior anti–GM-CSF/GM-CSF R and anti-IL-6/IL-6R | otilimab 90mg SC QW + csDMARDs | GM-CSF | 156 | 56.7 | 86% | 12.24 | 5.69 | | 38.09 | |
| | | | | otilimab 150mg SC QW + csDMARDs | GM-CSF | 158 | 56 | 85% | 10.73 | 5.81 | | 39.42 | |
| | | | | sarilumab 200mg SC Q2W + csDMARDs | IL-6R | 156 | 57.5 | 85% | 13.17 | 5.76 | | 39.55 | |
| | | | | placebo + csDMARDs | | 79 | 55.5 | 82% | 11.35 | 5.6 | | 36.06 | |
| Rubbert-Roth 2020 [4] | SELECT-CHOICE | 3 | Active RA; bDMARD-IR | upadacitinib 15mg PO QD+ csDMARD | JAK1 | 303 | 55.3 | 82.20% | 12.4 | 5.7 | 6.3 | 38.5 | |
| | | | | abatacept 500/1000mg Q2W+ csDMARD | CD80/CD86 (blocks CD28-mediated T-cell co-stimulation) | 309 | 55.8 | 81.90% | 11.8 | 5.9 | 6.4 | 40.7 | |
| Genovese 2019 [5] | FINCH 2 | 3 | Active RA; bDMARD-IR or intolerance | filgotinib 100 mg PO QD + csDMARDs | JAK1 | 153 | 55 | 77.80% | 10.3 | 5.9 | | 40.4 | 42.6 |
| | | | | filgotinib 200 mg/day PO QD + csDMARDs | JAK1 | 147 | 56 | 81.60% | 9.8 | 5.9 | | 41.7 | 43.4 |
| | | | | placebo + csDMARDs | | 148 | 56 | 81.80% | 9.9 | 5.9 | | 41.4 | 43 |
| Genovese 2018 [6] | SELECT-BEYOND | 3 | Active RA; bDMARD-IR or intolerance; non–anti-TNFbiologic-IR allowed | upadacitinib 15 mg PO QD + csDMARDs | JAK1 | 164 | 56·3 | 84% | 12·4 | 5.9 | | 41.7 | 43.3 |
| | | | | upadacitinib 30 mg PO QD + csDMARDs | JAK1 | 165 | 57·3 | 84% | 12·7 | 5.8 | | 40.1 | 41.7 |

| | | | | | | | | | | | | | |
|---|---|---|---|---|---|---|---|---|---|---|---|---|---|
| | | | | placebo + csDMARDs | | 169 | 57·6 | 85% | 14·5 | 5.8 | | 41 | 42.6 |
| Genovese 2016 [7] (BC for Genovese 2018) [8] | RA-BEACON | 3 | Active RA; TNFi-IR or intolerance; non–anti-TNFbiologic-IR allowed | baricitinib 2 mg PO QD + csDMARDs | JAK1/2 | 174 | 55 | 79% | 14 | 6 | 6.7 | | 45 |
| | | | | baricitinib 4mg PO QD + csDMARDs | JAK1/2 | 177 | 56 | 84% | 14 | 5.9 | 6.6 | | 42 |
| | | | | placebo + csDMARDs | | 176 | 56 | 82% | 14 | 5.9 | 6.6 | | 43 |
| Kremer 2016 [9] | BALANCE I | 2b | Active RA; TNFi-IR; non–anti-TNFbiologic-IR allowed | upadacitinib 3 mg PO BID + MTX | JAK1 | 55 | 57 | 78% | 11.8 | 5.7 | | 40 | |
| | | | | upadacitinib 6 mg PO BID + MTX | JAK1 | 55 | 56 | 78% | 12.3 | 5.9 | | 42 | |
| | | | | upadacitinib 12 PO BID + MTX | JAK1 | 55 | 59 | 82% | 12.2 | 5.7 | | 40 | |
| | | | | upadacitinib 18 mg PO BID + MTX | JAK1 | 55 | 57 | 76% | 10.9 | 5.8 | | 41 | |
| | | | | placebo + MTX | | 56 | 58 | 86% | 12.1 | 5.8 | | 41 | |
| Burmester 2013 [10] | | 3 | Active RA; TNFi-IR | tofacitinib 5 mg PO BID + MTX | JAK1/3 | 133 | 55.4 | 85.00% | 13 | 5.4 | 6.5 | | |
| | | | | tofacitinib 10 mg PO BID + MTX | JAK1/3 | 134 | 55.1 | 86.50% | 12.6 | 5.3 | 6.4 | | |
| | | | | placebo + MTX | | 132 | 54.4 | 80.30% | 11.3 | 5.4 | 6.4 | | |
| Smolen 2009 [11] | GO-AFTER | 3 | Active RA; TNFi-IR | golimumab 50 mg SC Q4W[a] | TNFα (soluble and membrane-bound) | 153 | 55 | 74% | 9.6 | | 6.4 | | |
| | | | | golimumab 100 mg SC Q4W[a] | | 153 | 55 | 80% | 8.7 | | 6.1 | | |
| | | | | placebo[a] | | 155 | 54 | 85% | 9.8 | | 6.3 | | |
| Emery 2008 [12] | RADIATE | 3 | Active RA; TNFi-IR | tocilizumab 8 mg/kg, IVQ4W + MTX | IL-6 R | 170 | 53.9 | 84% | 12.6 | | 6.79 | | |
| | | | | tocilizumab, IV 4 mg/kg, Q4W + MTX | IL-6 R | 161 | 50.9 | 81% | 11 | | 6.78 | | |
| | | | | placebo + MTX | | 158 | 53.4 | 79% | 11.4 | | 6.8 | | |

RA, rheumatoid arthritis; b/tsDMARDs, biologic or targeted synthetic disease-modifying antirheumatic drugs; RCT, randomized controlled trial; N, number of participants; DAS28, Disease Activity Score in 28 joints; CRP, C-reactive protein; ESR, erythrocyte sedimentation rate; CDAI, Clinical Disease Activity Index; SDAI, Simplified Disease Activity Index; csDMARD, conventional synthetic disease-modifying antirheumatic drug; IR, inadequate response; JAKi, Janus kinase inhibitor; GM-CSF, granulocyte–macrophage colony-stimulating factor; IL-6R, interleukin-6 receptor; SC, subcutaneous; PO, oral; QW, once weekly; Q2W, every two weeks; BID, twice daily; QD, once daily; TNFi, tumor necrosis factor inhibitor; MTX, methotrexate; TNFα, tumor necrosis factor alpha; IV, intravenous; Q4W, every four weeks.

[a] Percentage of patients with no methotrexate, hydroxychloroquine, or sulfasalazine concomitant treatment were 29% (golimumab 50 mg), 29% (golimumab 100 mg), and 31% (placebo).

### 5.1.2 Pooled analyses of RCT data (Supplementary Table 7)

Supplementary Table 7. Study and baseline characteristics from pooled analysis of RCT data reporting efficacy outcomes in active RA patients with ≥2 prior b/tsDMARDs.

| Study ID | Trials included | Target | Treatment type | N[a] | Age (Year) | Female (Percent) | Disease duration (Year) | DAS28ESR |
|---|---|---|---|---|---|---|---|---|
| Tesser 2023 [13] | 7 Phase II trials; 6 phase III trials: ORAL Scan, ORAL Solo, ORAL Standard, ORAL Step, ORAL Start, ORAL Sync | JAK1/3 (lower potency JAK2) | tofacitinib 5 mg PO BID | 270 | 54.6 | 84.10% | 12 | 6.5 |
| | | | tofacitinib 10 mg PO BID | 289 | 54.6 | 81.70% | 12.2 | 6.4 |
| | | | placebo (with or without concomitant csDMARDs) | 223 | 53.7 | 80.3% | 11.6 | 6.4 |

RA, rheumatoid arthritis; b/tsDMARDs, biologic or targeted synthetic disease-modifying antirheumatic drugs; RCT, randomized controlled trial; N, number of participants; DAS28, Disease Activity Score in 28 joints; ESR, erythrocyte sedimentation rate; JAK, Janus kinase; PO, oral; BID, twice daily; csDMARD, conventional synthetic disease-modifying antirheumatic drug.

[a]Baseline characteristics reported here are for the group with inadequate response to bDMARDs.

## 5.2 PICO 2 (safety)

### 5.2.1 Observational Studies (Supplementary Table 8)

Supplementary Table 8. Study and baseline characteristics of safety outcomes in active RA patients with ≥2 prior b/tsDMARDs, observational studies

| Author, year | Population | Treatment | N | Age | Female% | RA duration | DAS28 | DAS28-CRP | DAS28-ESR | CDAI | SDAI |
|---|---|---|---|---|---|---|---|---|---|---|---|
| **Studies specifically on D2T RA population** | | | | | | | | | | | |
| Ochi 2022 [14] | Adult RA; initiating b/tsDMARD (TNFi, IL-6Ri, ABA, JAKi); D2T RA: Failed ≥2 different MoA of b/tsDMARDs; vD2T RA (very difficult-to-treat): Failed ≥3 MoA; b/tsDMARD-naïve; FIRST Registry, Japan (characteristics are for D2T RA group) | TNFi | 71 | 63.5 | 87.30% | 13.6 | | 4.9 | 5.7 | 27.4 | |
| | | IL-6Ri | 79 | 68.6 | 84.50% | 14.2 | | 5 | 5.9 | 28.9 | |
| | | ABA | 58 | 64.6 | 87.90% | 15.2 | | 4.6 | 5.3 | 26.7 | |
| | | JAKi | 145 | 60.5 | 83.20% | 13.7 | | 4.5 | 5.2 | 26.2 | |
| Anno 2025 [15] | Adult RA patients initiating JAKi, categorized into naïve, 1 prior failure, and ≥2 prior failures (with the ≥2 failures split into D2T RA and non-D2T RA according to EULAR criteria), multicenter cohort, Japan | JAKi (D2T RA) | 76 | 63 | 80.20% | 16.3 | | 4.4 | 5 | 22.1 | 23.9 |
| | | JAKi (non-D2T RA) | 25 | 64.6 | 96.00% | 22.6 | | 3.9 | 4.8 | 17.6 | 18.4 |
| Watanabe 2024 [16] | Adult RA; initiating b/tsDMARD (TNFi, IL-6Ri, ABA, JAKi); D2T RA defined as: (i) CDAI >10 or(ii) DAS28-ESR >3.2 or (iii) difficulty in reducing prednisone dose below 7.5mg and failed ≥2 different MoA of b/tsDMARDs; ANSWER cohort, Japan | TNFi | 310 | 62* | 84.80% | 12.45* | | | 4.26* | 14.42* | |
| | | IL-6Ri | 194 | 64* | 82.50% | 10* | | | 4.34* | 14.92* | |
| | | ABA | 147 | 64* | 82.30% | 12* | | | 4.14* | 14.6* | |
| | | JAKi | 175 | 65* | 82.30% | 138 | | | 4.23* | 15* | |
| **Other studies** | | | | | | | | | | | |
| Choy 2021 [17] | Adult RA; initiating bDMARD (ABA vs other bDMARDs) in any line of therapy; multi-centre UK chart review (2013–2017) | ABA /other bDMARDs | 213 | 55.2 | 71.40% | 7 | | 5.5 | 6.3 | | |
| Pombo-Suarez 2022 [18] | Adult RA; JAKi-IR; JAK-pot collaboration | bDMARDs | 1635 | 55.2 | 81.00% | 12.94 | | | | 23.42 | |
| | | JAKi | 365 | 57.44 | 85.20% | 14.46 | | | | 24.35 | |
| Lauper 2024 [19] | Adult RA; initiating a bDMARD (TNFi, ABA, RTX, or TCZ) as 1st–5th line therapy; UK BSRBR-RA cohort | ETA | 10643 | 57.2 | 76.80% | 13.1 | 5.8 | | | | |
| | | ADA | 7835 | 56.9 | 77.40% | 13.4 | 6.1 | | | | |
| | | INF | 4430 | 56.3 | 76.80% | 13.7 | 6.4 | | | | |
| | | CER | 1614 | 55.6 | 76.20% | 10.5 | 5.7 | | | | |

| | | | | | | | | | | | |
|---|---|---|---|---|---|---|---|---|---|---|---|
| | | RTX | 5556 | 60.2 | 78.10% | 16.5 | 5.7 | | | | |
| | | TCZ | 2633 | 58.7 | 79.40% | 15.5 | 5.3 | | | | |
| | | ABA | 1186 | 60.3 | 79.70% | 17.9 | 5.3 | | | | |
| Schaefer 2025 [20] | Adult RA; starting b/tsDMARD with csDMARD ≥1st line; without a history of prior malignancies except NMSC; German RABBIT register | JAKis (BARI, FILGO, TOFA, or UPA) (mostly BARI and TOFA) | 2285 | 59.3 | 76.80% | | | | 4.2 | 12 | |
| | | bDMARDs (ABA, ADA, CER, ETA, GOL, INF, RTX, SARI, or TCZ) | 4259 | 58.8 | 74% | | | | 4.2 | 11 | |
| Miyazaki 2025 [21] | Adult RA; JAKi-IR; FIRST Registry, Japan | bDMARDs | 45 | 60.3 | 84.40% | 108 M | | | | 20.7 | 22.1 |
| | | JAKi | 31 | 57.4 | 83.90% | 123 M | | | | 20.9 | 21.7 |
| Miyazaki 2025 [22] | Adult RA; bDMARD-IR; FIRST Registry, Japan | IL-6Ri (prior use of 2bDMARDs) | 84 | 62.6 | 75.80% | 99 M | | | 5.2 | 22.6 | 23.6 |
| | | JAKi (prior use of 2bDMARDs) | 51 | 60.7 | 89.7% | 84 M | | | 5.2 | 26.2 | 27.1 |
| | | IL-6Ri (prior use of ≧3 bDMARDs) | 82 | 61.8 | 85.50% | 167 M | | | 5.5 | 27.6 | 29.6 |
| | | JAKi (prior use of ≧3 bDMARDs) | 68 | 63.7 | 84.20% | 196 M | | | 5.7 | 28.3 | 30.6 |

RA: Rheumatoid arthritis; D2T RA: Difficult-to-treat RA; vD2T RA: Very difficult-to-treat RA; bDMARD: Biologic disease-modifying antirheumatic drug; tsDMARD: Targeted synthetic DMARD; JAKi: Janus kinase inhibitor; TNFi: Tumor necrosis factor inhibitor; IL-6Ri: Interleukin-6 receptor inhibitor; ETA: Etanercept; ADA: Adalimumab; INF: Infliximab; CER: Certolizumab pegol; RTX: Rituximab; TCZ: Tocilizumab; ABA: Abatacept; BARI: Baricitinib; FILGO: Filgotinib; TOFA: Tofacitinib; UPA: Upadacitinib; GOL: Golimumab; SARI: Sarilumab; csDMARD: Conventional synthetic DMARD; NMSC: Non-melanoma skin cancer.
FIRST: Frontiers of Rheumatoid Arthritis Cohort, Japan; ANSWER: Kansai Consortium for Well-being of Rheumatic Disease Patients, Japan; BSRBR-RA: British Society for Rheumatology Biologics Register for RA, UK; RABBIT: German biologics register; JAK-pot: International collaboration on JAK inhibitor effectiveness and safety.
*Values marked with * indicate median.

## 5.3 PICO 3 (Supplementary Table 9)

Supplementary Table 9. Study and baseline characteristics of studies included for PICO 3

| Study ID | Study Type | Treatment | Description | N | Age (Year) | Female (Percent) | Disease duration (Year) | DAS28-ESR | HAQ-DI | FACIT | Patient VAS (mm) | Physician VAS (mm) |
|---|---|---|---|---|---|---|---|---|---|---|---|---|
| Ambrosino 2020 [23] | Open-label, pilot RCT | Standard rehabilitation + home exergaming | 4-week in-hospital multidisciplinary rehabilitation program (including traditional exercises and Wii-Fit–based exergaming), followed by Nintendo Wii-Fit home exergaming for an additional 8 weeks (five selected games. around 10 min per game daily) + maintaining usual medical care. | 20 | 27.05 | 65 | | 3.68 | 1.8 | 21.25 | | |
| | | Standard rehabilitation only (habitual activity) | 4-week in-hospital multidisciplinary rehabilitation program (including traditional exercises and Wii-Fit–based exergaming), followed by maintaining their habitual physical activity at home during the 8-week follow-up, without home exergaming. | 20 | 27.85 | 60 | | 3.58 | 1.75 | 22.35 | | |
| Michou 2022 [24] | open-label RCT | Educational intervention + usual care | Usual rheumatology care plus a structured educational intervention consisting of a 40-minute educational DVD and one multidisciplinary group teleconference session delivered 2–4 weeks after randomization. | 56 | 61 | 77 | 8 | | | | | |
| | | Usual care | Usual rheumatology care alone (regular follow-up with treating rheumatologist and access to rheumatology nurse) | 55 | 59.2 | 73 | 9.2 | | | | | |
| Toyama 2025 [25] | Observational cohort study | Orthopaedic surgical intervention | Patients with D2T RA undergoing orthopaedic surgical intervention; usual pharmacological care continued. | 74 | 64.3 | 86 | 18.3 | 2.94 | 1.05 | | 37.4 | 31.7 |
| | | Orthopaedic surgical intervention | Patients without D2T RA undergoing orthopaedic surgical intervention; usual pharmacological care continued. | 74 | 63.8 | 84 | 18.5 | 3.24 | 0.69 | | 27.5 | 28.2 |

DAS28-ESR, Disease Activity Score in 28 joints using erythrocyte sedimentation rate; HAQ-DI, Health Assessment Questionnaire–Disability Index; FACIT, Functional Assessment of Chronic Illness Therapy (fatigue scale); VAS, visual analogue scale; D2T RA, difficult-to-treat rheumatoid arthritis; RCT, randomized controlled trial.

## 6. Risk of Bias Assessment

### 6.1 PICO 1 Studies

#### 6.1.1 Observational studies using NOS (Supplementary Table 10)

Supplementary Table 10. Risk of Bias assessment with Newcastle Ottawa Scale (NOS) for PICO 1 observational studies

| Study ID | Representatives of exposed cohort | Selection of the non-exposed cohort | Ascertainment of exposure | Outcome of interest was not present at start of study | Comparability of cohorts | Assessment of outcomes | Was follow-up long enough for outcomes to occur? | Adequacy of follow up of cohorts | Overall score (out of 9) | Risk of bias | comment |
|---|---|---|---|---|---|---|---|---|---|---|---|
| Atienza-Mateo 2024 [26] | | | * | * | | * | * | | 4 | High | Uniform ethnicity, moderate attrition, unadjusted comparison |
| Aymon 2025 [27] | * | * | * | | ** | | | | 5 | Moderate | heterogeneous outcome definitions (different registries); Short follow up for CV events and not adequate |
| Baker 2022 [28] | | * | * | * | * | * | * | | 6 | Moderate | US registry, voluntary, follow-up completeness NR |
| Baker 2025 [29] | | * | * | * | * | * | * | | 6 | Moderate | US registry, voluntary, follow-up completeness NR; potential residual confounding |
| Bitoun 2023 [30] | * | * | * | * | | * | * | | 6 | Moderate | unmeasured confounding for our outcome of interest; not enough completeness |
| Burton 2017 [31] | | * | * | | | | * | | 3 | High | Selection bias; no adjustment for confounding; incomplete outcome assessment, and unclear follow-up completeness |

| | | | | | | | | | | | |
|---|---|---|---|---|---|---|---|---|---|---|---|
| Cassone 2020 [32] | * | | * | | | | * | * | 4 | High | RA activity not systematically measured, no comparator and no control for confounding |
| Chen 2020 [33] | | * | | * | * | | | | 3 | High | US claims, possible selection bias; no data on disease activity, residual confounding, possible misclassification bias; not enough follow-up |
| Chen 2017 [34] | | | * | * | | * | | | 3 | High | China single center; lack of confounder adjustment, very short follow-up |
| Cho 2025 [35] | | * | | * | * | * | | * | 5 | Moderate | Korea claims data, not adjusted for disease activity, short follow-up |
| Di Muzio 2024 [36] | | | * | * | | * | * | | 4 | High | Single center, selection bias; no control group, not addressed confounders, attrition bias |
| England 2025 [37] | | * | * | * | ** | | * | * | 6 | Moderate | only veterans, selection bias, possible missingness of outcome |
| Fernández-Díaz 2020 [38] | | | * | * | | * | * | | 4 | High | Uniform ethnicity, moderate attrition, unadjusted comparison |
| Fernández-Díaz 2021 [39] | | | * | * | * | * | * | | 5 | Moderate | Uniform ethnicity, moderate attrition |
| Frideres 2025 | | * | * | * | ** | | * | | 6 | Moderate | selection bias, possible missingness of outcome |
| Fukuda 2019 [40] | | | * | * | | * | * | | 4 | High | Japan; selection bias; attrition bias |
| Gremese 2013 [41] | | | * | * | * | | * | * | 5 | Moderate | Italy. Possible selection bias; misclassification bias, |
| Haraoui 2024 [42] | | * | * | * | | * | | | 4 | High | Selection bias, not enough adjustment, attrition bias |
| Hoshida 2024 [43] | | * | * | * | | * | * | * | 6 | Moderate | Japan, highly selected; |

| | | | | | | | | | | | |
|---|---|---|---|---|---|---|---|---|---|---|---|
| Honne 2022 [44] | | | * | * | | * | * | | 4 | High | Japan, single center; no adjustment for confounders, lack of information on patient loss to follow-up. |
| Jeong 2021 [45] | | | * | * | | * | * | | 4 | High | Selection bias; no adjustment for main confounders; not report on loss to follow-up |
| Jeong 2022 [46] | | * | | * | * | | * | | 4 | High | Korean claims data; relatively short follow-up for tofacitinib; no data on RA disease activity |
| Jung 2022 [47] | | * | * | * | * | * | * | | 6 | Moderate | South Korea; residual confounding, follow-up loss NR |
| Kalyoncu 2022 [48] | * | | * | * | | * | * | | 5 | Moderate | Non-exposed cohort not applicable, high loss to follow-up |
| Kang 2020 [49] | | * | * | * | * | | * | | 5 | Moderate | US claims data; not adjusted for main confounders: smoking history, RA disease activity or duration, and severity of baseline pulmonary conditions, possible outcome misclassification |
| Kao 2025 [50] | | * | * | * | * | | * | | 5 | Moderate | Taiwan claims data, not a targeted or balanced subgroup in this study, no adjustment for disease activity as one of the key confounders, ICD codes, follow up |
| KARATAŞ 2023 [51] | | | * | * | | * | * | | 4 | High | Tertiary centers and BMIs at the beginning or end of rituximab treatment were not known, non-exposed cohort not applicable, A high percentage of patients were lost to follow-up |

| | | | | | | | | | | | |
|---|---|---|---|---|---|---|---|---|---|---|---|
| Kelly 2021 [52] | | | * | * | | * | * | | 4 | High | Selection bias, non-exposed cohort not applicable (without RA-ILD), lack of information on patient loss to follow-up. |
| Khosrow-Khavar 2022 [53] | | * | | | * | * | | * | 4 | High | US claims data; no data on disease activity, residual confounding; baseline outcome status uncertain, outcome defined via ICD codes; short follow up, |
| Kim 2024 [54] | | | * | * | | * | * | | 4 | High | South Korean registry; Lack of an applicable control group. A high percentage of patients were lost to follow-up attrition bias |
| Kim 2022 [55] | | | * | * | | * | * | * | 5 | Moderate | South Korea, single center, Non MTX users are not a valid comparator |
| Kunishita 2023 [56] | | | | * | | * | | * | 3 | High | Japan, possible selection bias; Lack of an applicable control group; follow-up not enough for malignancy. |
| Kuo 2024 [57] | | * | | * | * | | | | 3 | High | Taiwan (only Asian patients) and single center, potential exposure misclassification (last DMARD used at reactivation was defined as exposure without any window defined), outcome based on irregular monitoring, insufficient info on loss to follow-up, limited follow up for some drugs |
| Kuo 2020 [58] | | | | * | | * | * | * | 4 | High | Taiwan, single center; Lack of a control group; possible baseline misclassification bias |
| Kuo 2021 [59] | | | * | | | * | * | | 3 | High | Taiwan, single center; Lack of a control group. Possible baseline misclassification bias, incomplete follow-up information |

| | | | | | | | | | | | |
|---|---|---|---|---|---|---|---|---|---|---|---|
| Kuwana 2025 [60] | | * | | * | * | * | * | | 5 | Moderate | Not a targeted or balanced subgroup in this study, history of infection was physician-reported, residual confounding likely, loss to follow-up was not available for this subgroup |
| Lashen 2021 [61] | | | * | * | | * | | * | 4 | High | Egypt, single center; Lack of a control group, short follow-up |
| Lee 2020 [62] | | | * | | | * | * | | 3 | High | South Korea, single center; lack of a control group; risk of baseline outcome misclassification; unaddressed confounding; potential attrition bias |
| Lee 2023 [63] | | * | * | * | | * | * | | 5 | Moderate | South Korea, possible selection bias; confounding; unclear attrition |
| López-Maraver 2024 [64] | * | * | * | * | | * | * | | 6 | Moderate | Follow-up completeness was poor for RA-related outcomes, confounding not addressed for our outcome of interest |
| Maeyama 2024 [65] | | | * | * | * | * | | | 4 | High | Selection bias; comparator not from the same population; residual confounding |
| Md Yusof 2020 [66] | | * | * | * | | * | * | | 5 | Moderate | Selection bias; major confounders remain unmeasured; incomplete follow-up |
| Mena-Vazquez 2022 [67] | | | * | * | | * | * | | 4 | High | Possible selection bias; no comparator |
| Mitsuboshi 2021 [68] | | | | | | | | | 0 | High | Selection bias; exposure and outcomes unverified; major uncontrolled confounding |
| Mochizuki 2021 [69] | | | * | | | * | * | | 3 | High | Single center; no active drug comparator |
| Molina-Collada 2024 [70] | | * | * | * | * | * | | | 5 | Moderate | Selection bias of patients with a lower risk of malignancy; residual confounding for cancer stage or treatment history, short follow-up time for cancer recurrence, lost to follow-up not addressed |

| | | | | | | | | | | | |
|---|---|---|---|---|---|---|---|---|---|---|---|
| Mori 2021[71] | | * | * | * | | * | * | | 5 | Moderate | Selection bias; potential residual confounding; no clear reporting of attrition |
| Nakamura 2012 [72] | | * | * | | | * | * | | 4 | High | Japan, single center; baseline imbalances and no adjustment for confounding; confounding by indication, unclear attrition |
| Nakayama 2024 [73] | | * | * | * | | | * | | 4 | High | Japan, possibility of selection bias; no adjustment for disease activity and other comorbidities; unreliable outcome assessment; Loss to follow-up not reported |
| Novella-Navarro 2022 [74] | | * | * | * | | | * | | 4 | High | Single center, subjective outcome measures, unclear attrition, no adjustment for confounders |
| Otsuji 2024 [74] | | | * | * | | * | * | | 4 | High | Single center; no comparator; no adjustment for confounding; unclear report for attrition |
| Padovan 2016 [75] | | | * | * | | * | * | | 4 | High | Selection bias; no comparator; missing data |
| Pappas 2020 [76] | * | | * | * | | * | * | | 5 | Moderate | No adjustment for our outcome of interest |
| Priora 2024 [77] | | | * | * | | * | * | | 4 | High | Selection bias, not a good representative of patients with High CV risk, no control group, not enough confounder adjustment, missingness is not reported |
| Pundole 2020 [78] | | | * | * | * | * | * | | 4 | High | Single center, confounding by indication, not adjusted for disease activity, no information on loss to follow-up |
| Rudi 2024 [79] | * | * | * | * | * | * | * | * | 8 | Low | |
| Ruiz 2024 [80] | | | | * | * | * | * | * | 5 | Moderate | US claims data; no data on disease activity; different patient populations, no verified exposure |

| | | | | | | | | | | | |
|---|---|---|---|---|---|---|---|---|---|---|---|
| Ruiz 2025 [81] | | | | * | * | * | * | * | 5 | Moderate | US claims data; no data on disease activity; different patient populations, no verified exposure |
| Sagtaganov 2024 [82] | | | | | | * | * | * | 3 | High | Selection bias, single center; potential misclassification of exposure and baseline disease activity and pain; no statistical adjustment |
| Schäfer 2020 [83] | | * | * | * | * | * | * | | 6 | Moderate | some obesity/sex/therapy strata were small; possibility of selection bias. |
| Serrano-Combarro 2025 [84] | | | * | * | | * | * | | 4 | High | Selection bias, no comparator and variable attrition |
| Shen 2023 [85] | | * | * | * | * | * | * | | 6 | Moderate | China single center, propensity score matched but disease activity not considered |
| Shih 2025 [86] | | * | * | * | * | * | * | | 6 | Moderate | RA-ILD defined only by ICD codes; residual confounding for disease activity; attrition not mentioned |
| Shin 2021 [87] | | * | | * | | * | * | | 4 | High | Korea claims, possible selection bias; PSS but no data on RA disease activity and no adjusted comparison for our outcomes of interest |
| Shu 2021 [88] | | * | * | * | | * | * | | 5 | Moderate | Selection bias, single center; lack of confounder adjustments and unclear attrition |
| Specker 2022 [89] | | | * | * | | * | * | | 4 | High | Selection bias; no control. Since this is a subgroup analysis, there is no adjustment. Loss to follow-up not indicated |
| Suissa 2019 [90] | | * | * | | * | | * | | 4 | High | US claims data; no data on smoking status, severity of COPD and severity of RA; possible outcome misclassification, |

| | | | | | | | | | | | |
|---|---|---|---|---|---|---|---|---|---|---|---|
| Tardella 2022 [91] | | * | * | | | * | * | * | 5 | Moderate | Italy, single center, unadjusted comparisons |
| Triboulet 2025 [92] | | | * | * | | * | * | | 4 | High | Selection bias, no comparator and missing data |
| Tsuboi 2023 [93] | | | * | * | | | * | * | 4 | High | Japan, selection bias, all women; no comparator; subjective outcome assessments |
| Vallejo-Yagüe 2024 [94] | * | * | * | * | * | * | * | | 7 | Low | High missing data rates, possible residual confounding |
| Venerito 2023 [95] | * | | * | | | * | * | | 4 | High | No comparator group; unclear baseline AE status; high attrition for safety outcomes |
| Wang 2023 [96] | | * | * | | | * | | * | 4 | High | China, single center and limited generalizability; no adjustment for confounders, short follow-up; no confirmation that safety outcomes were absent at baseline |
| Westermann 2025 [97] | * | * | * | * | * | * | | * | 7 | Low | Not adjusted for disease activity, mean follow-up not adequate for TNFi |
| Yokoyama 2023 [98] | | | * | * | * | * | * | * | 5 | Moderate | Japan, RWD database; no true comparator; loss to follow-up cannot be quantified, attrition bias likely |
| Yoshimura 2024 [99] | | * | * | * | * | | * | | 5 | Moderate | Japan, tertiary center, selection bias; residual confounding, potential outcome misclassification bias (efficacy measured via drug retention rate which could be influenced by physician preference, tolerability, comorbidities); potential attrition bias |

| | | | | | | | | | | | |
|---|---|---|---|---|---|---|---|---|---|---|---|
| Yoshimura 2025 [100] | | | * | * | | * | * | | 4 | High | Japan, 2 hospitals; no treatment comparator; no clear demonstration of loss to follow-up |
| You 2025 [101] | | * | | * | * | * | | | 4 | High | Korea claims data, not adjusted for disease activity, data for baseline smoking not complete, short follow-up |

Studies assigned 0–4, 5–6, and ≥7 stars were considered as high, moderate and low risk of bias, respectively. [102]

### 6.1.2 RCTs using RoB 2.0 (Figure S1)

| Study ID | D1 | D2 | D3 | D4 | D5 | Overall |
|---|---|---|---|---|---|---|
| Fernandez-Diaz 2023 | - | - | + | - | + | - |
| Fujieda 2021 | + | + | ! | + | + | ! |
| Latocha 2023 | + | ! | - | + | + | - |
| Mochizuki 2021 | ! | - | - | ! | ! | - |
| Weinblatt 2006 | + | ! | - | + | - | - |
| Xie 2019 | - | - | - | ! | - | - |
| Ytterberg 2021 | + | - | - | + | + | - |

+ Low risk

! Some concerns

- High risk

D1 Randomisation process

D2 Deviations from the intended interventions

D3 Missing outcome data

D4 Measurement of the outcome

D5 Selection of the reported result

Figure S1. Risk of Bias assessment of RCTs included for PICO 1, using RoB. 2.0

## 6.2 PICO 2 Studies

### 6.2.1 Observational studies using NOS (Supplementary Table 11)

Supplementary Table 11. Risk of Bias assessment using Newcastle Ottawa Scale (NOS) for safety outcomes in active RA patients with ≥2 prior b/tsDMARDs

| Study ID | Representatives of exposed cohort | Selection of the non-exposed cohort | Ascertainment of exposure | Outcome of interest was not present at start of study | Comparability of cohorts | Assessment of outcomes | Was follow-up long enough for outcomes to occur? | Adequacy of follow up of cohorts | Overall score (out of 9) | Risk of bias | Comment |
|---|---|---|---|---|---|---|---|---|---|---|---|
| Choy 2021 [17] | | * | * | | | * | * | | 4 | High | 4 UK tertiary centers; retrospective chart review; safety outcome; no adjustment for confounders related outcome of interest; substantial missing data and loss to follow-up |
| Lauper 2024 [19] | * | * | * | * | * | * | * | * | 8 | Low | UK; registry; adjusted for major confounders but not for prior infection or treatment year; possible healthy user bias |
| Miyazaki 2025 [22] | | * | * | | ** | * | | * | 6 | Moderate | Japan; registry; PS-IPTW adjustment; safety outcomes; outcome not confirmed absent at baseline; no sign of major attrition bias; relatively short follow-up |
| Ochi 2022 [14] | | * | * | | * | * | * | * | 6 | Moderate | Japan; registry; outcome not confirmed absent at baseline; adjusted for age, sex, MTX dose, and glucocorticoid use, no sign of major attrition bias |
| Watanabe 2024 [16] | | * | * | | * | * | * | * | 6 | Moderate | Japan; multi center registry; outcome not confirmed absent at baseline; no sign of major attrition bias |

| | | | | | | | | | | | |
|---|---|---|---|---|---|---|---|---|---|---|---|
| Anno 2025 [15] | | * | * | | | * | | * | 4 | High | Japan; multicenter database of tertiary centers; no adjustment for confounders related to outcome of interest; outcome not confirmed absent at baseline; relatively short follow-up |
| Schaefer 2025 [20] | | * | * | * | ** | * | * | * | 9 | Low | RABBIT registry; IPTW-adjusted; 4-year follow-up; minimal attrition |
| Miyazaki 2025 [21] | | * | * | | ** | * | | * | 6 | Moderate | Japan; registry; PS-IPTW adjustment; safety outcomes; outcome not confirmed absent at baseline; no sign of major attrition bias; relatively short follow-up |
| Pombo-Suarez 2022 [18] | * | * | * | * | | * | * | * | 7 | Low | Multiple national registries; crude report of our outcome of interest (discontinuation due to adverse event) |

Studies assigned 0–4, 5–6, and ≥7 stars were considered as high, moderate and low risk of bias, respectively. [102]

## 6.2.2 RCTs using RoB 2.0 (Figure S2)

| Study ID | D1 | D2 | D3 | D4 | D5 | Overall | Notes |
|---|---|---|---|---|---|---|---|
| Burmester 2013 | + | + | ! | + | - | - | Post hoc subgroup analysis |
| Smolen 2009 | + | + | ! | + | - | - | Exploratory subgroup analysis |
| Emery 2008 | + | + | ! | + | - | - | Exploratory subgroup analysis |
| Kremer 2016 | + | + | ! | + | - | - | Exploratory subgroup analysis |
| Genovese 2018, RA-BEACON | + | + | ! | + | - | - | ≥2 TNFi was post hoc , ≥3 bDMARDs was prespecified but not all pre-specified subgroups are reported. |
| Genovese 2018, SELECT-BEYOND | + | + | ! | + | - | - | Exploratory subgroup analysis |
| Genovese 2019 | + | + | ! | + | - | - | Subgroup analysis was prespecified but not all pre-specified subgroups are reported |
| Rubbert-Roth 2020 | + | + | ! | + | + | ! | |
| Fleischmann 2023 | + | + | ! | + | + | ! | |
| Taylor 2023 | + | + | ! | + | + | ! | |

For all studies, D3 has some concerns because missing data is unclear for the subgroups

| | | | |
|---|---|---|---|
| + | Low risk | D1 | Randomisation process |
| ! | Some concerns | D2 | Deviations from the intended interventions |
| - | High risk | D3 | Missing outcome data |
| | | D4 | Measurement of the outcome |
| | | D5 | Selection of the reported result |

Figure S2. Risk of Bias assessment of RCTs reporting efficacy outcomes in active RA patients with ≥2 prior b/tsDMARDs using RoB. 2.0

## 6.3 PICO 3 Studies

### 6.3.1 Observational Study using NOS (Supplementary Table 12)

Supplementary Table 12. Risk of Bias assessment using Newcastle Ottawa Scale (NOS) for PICO 3 studies

| Study ID | Representatives of exposed cohort | Selection of the non-exposed cohort | Ascertainment of exposure | Outcome of interest was not present at start of study | Comparability of cohorts | Assessment of outcomes | Was follow-up long enough for outcomes to occur? | Adequacy of follow up of cohorts | Overall score (out of 9) | Risk of bias | comment |
|---|---|---|---|---|---|---|---|---|---|---|---|
| Toyama 2025 [25] | | | * | * | * | * | * | | 5 | Moderate | Single center, attrition bias, comparator not from the same population, residual confounding |

Studies assigned 0–4, 5–6, and ≥7 stars were considered as high, moderate and low risk of bias, respectively. [102]

### 6.3.2 RCTs using RoB 2.0 (Figure S3)

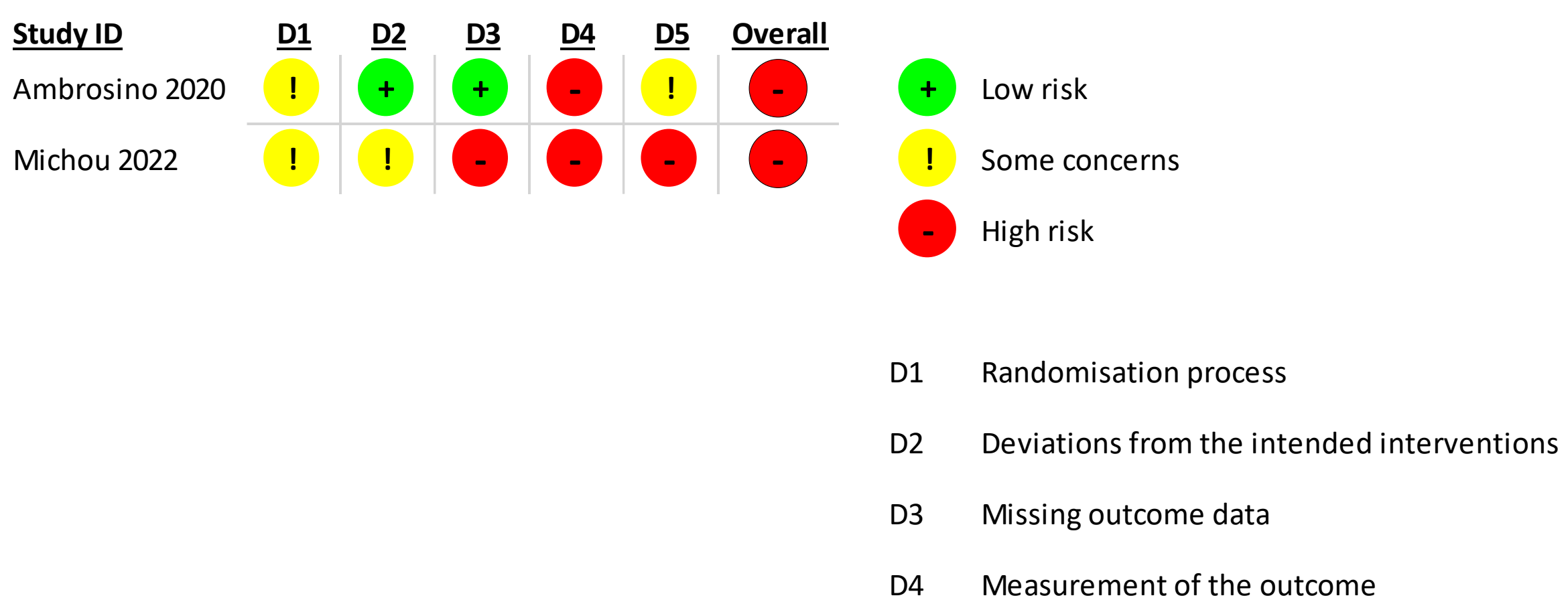


Figure S3. Risk of Bias assessment of RCTs included for PICO 3

# 7. Additional Results

## 7.1 PICO2 safety outcomes (Supplementary Table 13)

Supplementary Table 13. Studies reporting safety outcomes in active RA patients with ≥2 prior b/tsDMARDs

| Author, year | Treatment | Number of failed b/tsDMARD | N | Any AE, n (%) | SAE, n (%) | AEs leading to TD, n (%) | Any infection, n (%) | Serious infection, n (%) | Infection leading to TD | Herpes zoster, n (%) | Malignancy, n (%) | Other outcomes | Description |
|---|---|---|---|---|---|---|---|---|---|---|---|---|---|
| Choy 2021 [17] | ABA | 2 | 8 | | | | s [a] | | s | | | | |
| | other bDMARDs | 2 | 30 | | | | 13 [a] | | 12 | | | | |
| | ABA | 3 | 7 | | | | s [a] | | s | | | | |
| | other bDMARDs | 3 | 12 | | | | 5 [a] | | 5 | | | | |
| Lauper 2024 [19] | ETA | 2 | 536 | | | | | 32 (5.97) | | | | ref | |
| | ADA | 2 | 596 | | | | | 44 (7.38) | | | | HR (95% CI):1.3 (0.8, 2.1) | serious infection |
| | INF | 2 | 267 | | | | | 15 (5.62) | | | | HR (95% CI): 0.8 (0.4, 1.8) | serious infection |
| | CER | 2 | 129 | | | | | 5 (3.88) | | | | HR (95% CI): 0.9 (0.3, 3.0) | serious infection |
| | RTX | 2 | 1846 | | | | | 115 (3.23) | | | | HR (95% CI): 0.7 (0.5, 1.2) | serious infection |
| | TCZ | 2 | 923 | | | | | 73 (7.91) | | | | HR (95% CI): 1.4 (0.9, 2.3) | serious infection |
| | ABA | 2 | 367 | | | | | 23 (6.27) | | | | HR (95% CI): 1.5 (0.7, 2.9) | serious infection |
| | ETA | 3 | 147 | | | | | 8 (5.44) | | | | ref | |
| | ADA | 3 | 107 | | | | | 7 (6.54) | | | | HR (95% CI):1.3 (0.5, 3.8) | serious infection |
| | INF | 3 | 82 | | | | | 7 (8.54) | | | | HR (95% CI): 1.4 (0.4, 4.4) | serious infection |
| | CER | 3 | 83 | | | | | 1 (1.2) | | | | HR (95% CI): 0.2 (0.0, 1.9) | serious infection |
| | RTX | 3 | 568 | | | | | 46 (8.1) | | | | HR (95% CI): 1.1 (0.5, 2.3) | serious infection |
| | TCZ | 3 | 596 | | | | | 37 (6.21) | | | | HR (95% CI): 1.0 (0.5, 2.4) | serious infection |
| | ABA | 3 | 379 | | | | | 21 (5.54) | | | | HR (95% CI):1.0 (0.4, 2.4) | serious infection |
| | ETA | 4 | 48 | | | | | 3 (6.25) | | | | ref | |
| | ADA | 4 | 40 | | | | | 4 (10.0) | | | | HR (95% CI): 2.4 (0.5, 11.7) | serious infection |
| | INF | 4 | 22 | | | | | 2 (9.09) | | | | HR (95% CI): 0.7 (0.1, 7.3) | serious infection |
| | CER | 4 | 41 | | | | | 4 (9.76) | | | | HR (95% CI): 2.8 (0.5, 14.5) | serious infection |
| | RTX | 4 | 144 | | | | | 13 (9.06) | | | | HR (95% CI): 1.0 (0.2, 4.1) | serious infection |
| | TCZ | 4 | 229 | | | | | 18 (7.86) | | | | HR (95% CI): 0.7 (0.2, 3.1) | serious infection |
| | ABA | 4 | 239 | | | | | 13 (5.44) | | | | HR (95% CI): 0.8 (0.2, 3.4) | serious infection |

| | | | | | | | | | | | | | |
|---|---|---|---|---|---|---|---|---|---|---|---|---|---|
| Miyazaki 2025 [22] | IL-6Ri | 2 | 84 | 19 (22.5) | 7 (7.9) | | 12 (14.8) | 0 (0.0) | | 0 (0.0) | 0 (0.0) | | |
| | JAKi | 2 | 51 | 12 (24.1) | 2 (4.4) | | 10 (20.6) | 0 (0.0) | | **3 (5.4)** | 1 (1.1) | | |
| | IL-6Ri | ≥3 | 82 | 17 (20.5) | 11 (13.1) | | 9 (11.0) | 4 (5.1) | | 0 (0.0) | 2 (2.0) | | |
| | JAKi | ≥3 | 68 | 21 (30.8) | 3 (4.3) | | **18 (26.7)** | 1 (1.7) | | 2 (2.4) | 0 (0.0) | | |
| Ochi 2022 [14 15] | TNFi | ≥2 | 71 | | | 11 (15.5) | | | | | 0 | ref | |
| | IL-6Ri | ≥2 | 79 | | | 7 (8.9) | | | | | 1 | HR (95% CI): 0.64 (0.22, 1.87) | severe AE |
| | ABA | ≥2 | 58 | | | 5 (8.6) | | | | | 1 | HR (95% CI): 0.45 (0.12, 1.71) | severe AE |
| | JAKi | ≥2 | 145 | | | 10 (6.7) | | | | | 1 | HR (95% CI): 0.37 (0.13, 1.08) | severe AE |
| Watanabe 2024 [16] | TNFi | ≥2 | 310 | | | | | | | | | 1 | |
| | IL-6Ri | ≥2 | 194 | | | | | | | | | HR (95% CI): 1.18 (0.73, 1.91) | TD due to toxic AE |
| | ABA | ≥2 | 147 | | | | | | | | | HR (95% CI): 1.16 (0.67, 2.00) | TD due to toxic AE |
| | JAKi | ≥2 | 175 | | | | | | | | | HR (95% CI): 0.85 (0.49, 1.47) | TD due to toxic AE |
| Anno 2025 [15] | JAKi | ≥2 (true D2TRA) | 76 | | | 9 (11.8) | | | 3 (3.9) | | | | |
| | JAKi | ≥2 (non-D2TRA) | 25 | | | 0 | | | 0 | | | | |
| Schaefer 2025 [20] | JAKi | ≥3 b/tsDMARD | | | | | | | | | | **HR (95% CI): 1.98 (1.01-3.90)** | incident malignancies [b] |
| | bDMARDs | ≥3 b/tsDMARD | | | | | | | | | | ref | |
| | JAKi | ≥3 TNFi/IL6i failure | 447 | | | | | | | | | HR (95% CI): 1.30 (0.68-2.48) | incident malignancies [b] |
| | bDMARDs | ≥3 TNFi/IL6i failure | 403 | | | | | | | | | ref | |
| | JAKi | ≥3 b/tsDMARD and ≥ 60Y | | | | | | | | | | **HR (95% CI): 1.98 (1.01-3.90)** | incident malignancies [b] |
| | bDMARDs | ≥3 b/tsDMARD and ≥ 60Y | | | | | | | | | | ref | |
| | JAKi | ≥3 TNFi/IL6i failure ≥ 60Y | | | | | | | | | | HR (95% CI): 1.72 (0.71-4.16) | incident malignancies [b] |

| | | | | | | | | | | | |
|---|---|---|---|---|---|---|---|---|---|---|---|
| | bDMARDs | ≥3 TNFi/IL6i failure ≥ 60Y | | | | | | | | ref | |
| | JAKi | ≥3 b/tsDMARD and < 60Y | | | | | | | | HR (95% CI): 0.82 (0.41-1.65) | incident malignancies [b] |
| | bDMARDs | ≥3 b/tsDMARD and < 60Y | | | | | | | | ref | |
| | JAKi | ≥3 TNFi/IL6i failure < 60Y | | | | | | | | HR (95% CI): 0.80 (0.32-2.05) | incident malignancies b |
| | bDMARDs | ≥3 TNFi/IL6i failure < 60Y | | | | | | | | ref | |
| Miyazaki 2025 [21] | JAKi→nonTNFi bDMARD | ≥2 | 29 | 3 (10.3) | 0 | 3 (10.3) | 0 | | 0 | | |
| | JAKi→TNFi | ≥2 | 16 | 6 (37.5) | 2 (12.5) | 3 (18.8) | 0 | | 0 | | |
| | JAKi→JAKi | ≥2 | 31 | 7 (22.6) | 0 | 6 (19.4) | 0 | 1 (3.2) | 0 | | |

RA: Rheumatoid arthritis; D2T RA: Difficult-to-treat RA; bDMARD: Biologic disease-modifying antirheumatic drug; tsDMARD: Targeted synthetic DMARD; JAKi: Janus kinase inhibitor; TNFi: Tumor necrosis factor inhibitor; IL-6Ri: Interleukin-6 receptor inhibitor; ETA: Etanercept; ADA: Adalimumab; INF: Infliximab; CER: Certolizumab pegol; RTX: Rituximab; TCZ: Tocilizumab; ABA: Abatacept; BARI: Baricitinib; FILGO: Filgotinib; TOFA: Tofacitinib; UPA: Upadacitinib; GOL: Golimumab; SARI: Sarilumab.
AE: Adverse event; SAE: Serious adverse event; TD: Treatment discontinuation; HR: Hazard ratio; CI: Confidence interval.
a: Infection requiring antibiotic.
b: Malignancies excluding non-melanoma skin cancer.
Bolded values indicate statistically significant results.

## 7.2 Meta-regression results

### 7.2.1 RR of ACR20 Response (Supplementary Table 14)

Supplementary Table 14. Meta-regression analysis of prior bDMARD failures as moderator of RR of ACR20 Response (JAKi vs Placebo)

| Moderator (binary variable) | Comparisons (n) | β (log RR) | 95% CI | p-value | Residual Heterogeneity (QE, p-value) |
|---|---|---|---|---|---|
| ≥2 vs 1 prior bDMARD failure (ref: 1) | 14 | 0.344 | (0.191–0.497) | 0.0004 | 3.95, p = 0.984 |
| ≥3 vs ≥2 prior bDMARD failures (ref: ≥2) | 10 | 0.449 | (0.107–0.791) | 0.016 | 3.33, p = 0.911 |

bDMARD, biologic disease-modifying antirheumatic drug; RR, relative risk; ACR20, American College of Rheumatology 20% response; JAKi, Janus kinase inhibitor; β = regression coefficient on log (RR) scale; CI, confidence interval; QE, Cochran's Q statistic for residual heterogeneity; ref: reference.
Genovese 2019 (filgotinib)[5] reported separate data for exactly 2 and ≥3 prior bDMARD failures. These strata were kept separate in the ≥2 vs ≥3 comparison to prevent overlap, but combined as ≥2 for the 1 vs ≥2 comparison and pooled estimates.

### 7.2.1 Absolute ACR20 Response (Supplementary Table 15)

Supplementary Table 15. Meta-regression analysis of prior bDMARD failures as moderator of absolute ACR20 Response of JAKi

| Moderator (binary variable) | Comparisons (n) | β (log RR) | 95% CI | p-value | Residual Heterogeneity (QE, p-value) |
|---|---|---|---|---|---|
| ≥2 vs 1 prior bDMARD failure (ref: 1) | 14 | -0.0150 | (-0.526–0.496) | 0.95 | 37.189, p = 0.0002 |
| ≥3 vs ≥2 prior bDMARD failures (ref: ≥2) | 10 | -0.059 | (-0.845–0.728) | 0.87 | 25.739, p = 0.001 |

bDMARD, biologic disease-modifying antirheumatic drug; ACR20, American College of Rheumatology 20% response; JAKi, Janus kinase inhibitor; β = regression coefficient on log (RR) scale; CI, confidence interval; QE, Cochran's Q statistic for residual heterogeneity; ref: reference.
Genovese 2019 (filgotinib)[5] reported separate data for exactly 2 and ≥3 prior bDMARD failures. These strata were kept separate in the ≥2 vs ≥3 comparison to prevent overlap, but combined as ≥2 for the 1 vs ≥2 comparison and pooled estimates.

## 7.3. Operationalization of EULAR D2T RA components in Studies (Supplementary Table 16)

Supplementary Table 16. Operationalization of EULAR D2T RA components in studies that directly enrolled EULAR-defined D2T RA patients

| EULAR D2T RA components | EULAR D2T RA definition | Ochi 2022[14] | Watanabe 2024[16] | Anno 2025[15] | Toyama 2025[25] |
|---|---|---|---|---|---|
| **1. Previous treatment failures** | Failure of ≥2 b/tsDMARDs (with different MoA)* after failing csDMARD therapy (unless contraindicated).† | ✓ Operationalized as: failure of ≥2 b/tsDMARDs with different MoA | ✓ Operationalized as: failure of ≥2 b/tsDMARDs with different MoA | ✓ Operationalized as: failure of ≥2 b/tsDMARDs with different MoA | ✓ |
| **2. Signs suggestive of active/progressive disease** (≥1 of a–e) | a. At least moderate disease activity (according to validated composite measures including joint counts, for example, DAS28-ESR>3.2 or CDAI>10). | Not stated | ✓ | Probably implied (but not explicitly stated) | ✓ (met by 32.4% of D2T RA patients) |
| | b. Signs (including acute phase reactants and imaging) and/or symptoms suggestive of active disease (joint related or other). | Not stated | Not stated | Not stated | ✓ Operationalized as: CRP level of ≥1.0mg/dl or a Rooney score of ≥21 (47.3%) |
| | c. Inability to taper glucocorticoid treatment (below 7.5mg/day prednisone or equivalent). | Not stated | ✓ | Not stated | ✓ (2.7%) |
| | d. Rapid radiographic progression (with or without signs of active disease).‡ | Not stated | Not stated | Not stated | ✓ Operationalized as: Larsen grade ≥3 (98.6%) |
| | e. Well-controlled disease according to above standards, but still having RA symptoms that are causing a reduction in quality of life. | Not stated | Not stated | Not stated | ✓ Operationalized as: patient complaint attributed to RA in ≥1 joint (100%) |
| **3. Problematic perception of disease management** | The management of signs and/or symptoms is perceived as problematic by the rheumatologist and/or the patient. | Not stated | Not stated | Not stated | Not stated |

All three criteria need to be present in D2T RA.

b, biological; CDAI, clinical disease activity index; cs, conventional synthetic; DAS28-ESR, disease activity score assessing 28 joints using erythrocyte sedimentation rate; DMARD, disease-modifying antirheumatic drug; mg, milligram; EULAR, European League Against Rheumatism; RA, rheumatoid arthritis; ts, targeted synthetic.

*Unless restricted by access to treatment due to socioeconomic factors.

†Treatment according to EULAR recommendation and if csDMARD treatment is contraindicated, failure of ≥2 b/tsDMARDs with different mechanisms of action is sufficient.

‡Rapid radiographic progression: change in van der Heijde-modified Sharp score ≥5 points at 1 year.

# References


1. Roodenrijs NM, Hamar A, Kedves M, et al. Pharmacological and non-pharmacological therapeutic strategies in difficult-to-treat rheumatoid arthritis: a systematic literature review informing the EULAR recommendations for the management of difficult-to-treat rheumatoid arthritis. *RMD open* 2021;7(1)
2. Fleischmann RM, Van Der Heijde D, Strand V, et al. Anti-GM-CSF otilimab versus tofacitinib or placebo in patients with active rheumatoid arthritis and an inadequate response to conventional or biologic DMARDs: two phase 3 randomised trials (contRAst 1 and contRAst 2). *Annals of the rheumatic diseases* 2023;82(12):1516. doi: doi:https://doi.org/10.1136/ard-2023-224482
3. Taylor PC, Weinblatt ME, McInnes IB, et al. Anti-GM-CSF otilimab versus sarilumab or placebo in patients with rheumatoid arthritis and inadequate response to targeted therapies: a phase III randomised trial (contRAst 3). *Annals of the rheumatic diseases* 2023;82(12):1527. doi: doi:https://doi.org/10.1136/ard-2023-224449
4. Rubbert-Roth A, Enejosa J, Pangan AL, et al. Trial of upadacitinib or abatacept in rheumatoid arthritis. *New England Journal of Medicine* 2020;383(16):1511-21.
5. Genovese MC, Kalunian K, Gottenberg JE, et al. Effect of Filgotinib vs Placebo on Clinical Response in Patients with Moderate to Severe Rheumatoid Arthritis Refractory to Disease-Modifying Antirheumatic Drug Therapy: The FINCH 2 Randomized Clinical Trial. *JAMA* 2019;322(4):315-25. doi: doi:10.1001/jama.2019.9055
6. Genovese MC, Fleischmann R, Combe B, et al. Safety and efficacy of upadacitinib in patients with active rheumatoid arthritis refractory to biologic disease-modifying anti-rheumatic drugs (SELECT-BEYOND): a double-blind, randomised controlled phase 3 trial. *The Lancet* 2018;391(10139):2513-24.
7. Genovese MC, Kremer J, Zamani O, et al. Baricitinib in patients with refractory rheumatoid arthritis. *New England Journal of Medicine* 2016;374(13):1243-52.
8. Genovese MC, Kremer JM, Kartman CE, et al. Response to baricitinib based on prior biologic use in patients with refractory rheumatoid arthritis. *Rheumatology (United Kingdom)* 2018;57(5):900-08. doi: doi:10.1093/rheumatology/kex489
9. Kremer JM, Emery P, Camp HS, et al. A phase IIb study of ABT-494, a selective JAK-1 inhibitor, in patients with rheumatoid arthritis and an inadequate response to anti–tumor necrosis factor therapy. *Arthritis & rheumatology* 2016;68(12):2867-77.
10. Burmester GR, Blanco R, Charles-Schoeman C, et al. Tofacitinib (CP-690,550) in combination with methotrexate in patients with active rheumatoid arthritis with an inadequate response to tumour necrosis factor inhibitors: a randomised phase 3 trial. *The Lancet* 2013;381(9865):451-60.
11. Smolen JS, Kay J, Doyle MK, et al. Golimumab in patients with active rheumatoid arthritis after treatment with tumour necrosis factor α inhibitors (GO-AFTER study): a multicentre, randomised, double-blind, placebo-controlled, phase III trial. *The Lancet* 2009;374(9685):210-21.
12. Emery P, Keystone E, Tony H, et al. IL-6 receptor inhibition with tocilizumab improves treatment outcomes in patients with rheumatoid arthritis refractory to anti-tumour necrosis factor biologicals: results from a 24-week multicentre randomised placebo-controlled trial. *Annals of the rheumatic diseases* 2008;67(11):1516-23.
13. Tesser J, Gül A, Olech E, et al. Efficacy and safety of tofacitinib in patients with rheumatoid arthritis by previous treatment: post hoc analysis of phase II/III trials. *Arthritis Research and Therapy* 2023;25(1) doi: doi:10.1186/s13075-023-03154-z

14. Ochi S, Sonomoto K, Nakayamada S, et al. Preferable outcome of Janus kinase inhibitors for a group of difficult-to-treat rheumatoid arthritis patients: from the FIRST Registry. *Arthritis research & therapy* 2022;24(1):61. doi: doi:https://dx.doi.org/10.1186/s13075-022-02744-7
15. Anno S, Okano T, Mamoto K, et al. Efficacy and safety of Janus kinase inhibitors in patients with difficult-to-treat rheumatoid arthritis. *Modern Rheumatology* 2025;35(2):225-33.
16. Watanabe R, Ebina K, Gon T, et al. Predictive factors and treatment outcomes associated with difficult-to-treat rheumatoid arthritis conditions: the ANSWER cohort study. *Rheumatology (United Kingdom)* 2024;63(9):2418-26. doi: doi:10.1093/rheumatology/keae265
17. Choy E, Groves L, Sugrue D, et al. Outcomes in rheumatoid arthritis patients treated with abatacept: a UK multi-centre observational study. *BMC Rheumatology* 2021;5(1) doi: doi:10.1186/s41927-020-00173-0
18. Pombo-Suarez M, Sanchez-Piedra C, Gómez-Reino J, et al. After JAK inhibitor failure: to cycle or to switch, that is the question–data from the JAK-pot collaboration of registries. *Annals of the rheumatic diseases* 2023;82(2):175-81.
19. Lauper K, Kearsley-Fleet L, Galloway JB, et al. Evaluation of serious infections, including Mycobacterium tuberculosis, during treatment with biologic disease-modifying anti-rheumatic drugs: does line of therapy matter? *Rheumatology (Oxford, England)* 2024;63(7):1957-64. doi: doi:https://dx.doi.org/10.1093/rheumatology/kead515
20. Schaefer M, Purschke A, Zietemann V, et al. Comparative risk of incident malignancies in rheumatoid arthritis patients treated with Janus kinase inhibitors or bDMARDs: observational data from the German RABBIT register. *Annals of the Rheumatic Diseases* 2025 doi: 10.1016/j.ard.2025.05.014
21. Miyazaki Y, Nakayamada S, Tanaka H, et al. Switching to biological DMARDs versus cycling among JAK inhibitors in patients with rheumatoid arthritis and with inadequate response to JAK inhibitors: from FIRST registry. *RMD Open* 2025;11(1) doi: doi:10.1136/rmdopen-2024-004987
22. Miyazaki Y, Nakayamada S, Tanaka H, et al. Comparison of anti-IL-6 receptor and JAK inhibitors in patients with rheumatoid arthritis from the real-world practice FIRST registry. *Rheumatology* 2025;64(4):1627-36. doi: doi:10.1093/rheumatology/keae334
23. Ambrosino P, Iannuzzi GL, Formisano R, et al. Exergaming as an Additional Tool in Rehabilitation of Young Patients with Rheumatoid Arthritis: a Pilot Randomized Controlled Trial. *Games for health journal* 2020;9(5):368. doi: doi:https://doi.org/10.1089/g4h.2019.0167
24. Michou L, Julien AS, Witteman HO, et al. Measuring the impact of an educational intervention in rheumatoid arthritis: an open-label, randomized trial. *Archives of rheumatology* 2022;37(2):169. doi: doi:https://doi.org/10.46497/ArchRheumatol.2022.8965
25. Toyama S, Ishikawa H, Abe A, et al. Impact of orthopaedic surgical intervention on difficult-to-treat rheumatoid arthritis: A propensity score-matched study. *Modern Rheumatology* 2025;35(3):434-42.
26. Atienza-Mateo B, Fernandez-Diaz C, Vicente-Rabaneda EF, et al. Abatacept in usual and in non-specific interstitial pneumonia associated with rheumatoid arthritis. *European journal of internal medicine* 2024;119:118-24. doi: doi:https://dx.doi.org/10.1016/j.ejim.2023.08.025
27. Aymon R, Mongin D, Guemara R, et al. Incidence of Major Adverse Cardiovascular Events in Patients With Rheumatoid Arthritis Treated With JAK Inhibitors Compared With Biologic Disease-Modifying Antirheumatic Drugs: Data From an International Collaboration of Registries. *Arthritis and Rheumatology* 2025 doi: doi:10.1002/art.43188
28. Baker JF, Reed G, Poudel DR, et al. Obesity and Response to Advanced Therapies in Rheumatoid Arthritis. *Arthritis care & research* 2022;74(11):1909-16. doi: doi:https://dx.doi.org/10.1002/acr.24867
29. Baker JF, Reed G, Mikuls TR, et al. Metabolic Syndrome, Adipokines, and Response to Advanced Therapies in Rheumatoid Arthritis. *Arthritis and Rheumatology* 2025;77(3):263-71. doi: 10.1002/art.43034

30. Bitoun S, Hässler S, Ternant D, et al. Response to Biologic Drugs in Patients with Rheumatoid Arthritis and Antidrug Antibodies. *JAMA Network Open* 2023;6(7) doi: doi:10.1001/jamanetworkopen.2023.23098
31. Burton MJ, Curtis JR, Yang S, et al. Safety of biologic and nonbiologic disease-modifying antirheumatic drug therapy in veterans with rheumatoid arthritis and hepatitis c virus infection. *Journal of Rheumatology* 2017;44(5):565-70. doi: doi:10.3899/jrheum.160983
32. Cassone G, Manfredi A, Atzeni F, et al. Safety of abatacept in Italian patients with rheumatoid arthritis and interstitial lung disease: A multicenter retrospective study. *Journal of Clinical Medicine* 2020;9(1) doi: doi:10.3390/jcm9010277
33. Chen SK, Lee H, Jin Y, et al. Use of biologic or targeted-synthetic disease-modifying anti-rheumatic drugs and risk of diabetes treatment intensification in patients with rheumatoid arthritis and diabetes mellitus. *Rheumatology Advances in Practice* 2020;4(2):1-11. doi: doi:10.1093/rap/rkaa027
34. Chen LF, Mo YQ, Jing J, et al. Short-course tocilizumab increases risk of hepatitis B virus reactivation in patients with rheumatoid arthritis: a prospective clinical observation. *International Journal of Rheumatic Diseases* 2017;20(7):859-69.
35. Cho Y, Yoon D, Khosrow-Khavar F, et al. Cardiovascular, cancer, and infection risks of Janus kinase inhibitors in rheumatoid arthritis and ulcerative colitis: A nationwide cohort study. *Journal of internal medicine* 2025;297(4):366-81. doi: doi:https://dx.doi.org/10.1111/joim.20064
36. Di Muzio C, Di Cola I, Shariat Panahi A, et al. The effects of suppressing inflammation by tofacitinib may simultaneously improve glycaemic parameters and inflammatory markers in rheumatoid arthritis patients with comorbid type 2 diabetes: a proof-of-concept, open, prospective, clinical study. *Arthritis Research and Therapy* 2024;26(1) doi: doi:10.1186/s13075-023-03249-7
37. England BR, Baker JF, George MD, et al. Advanced therapies in US veterans with rheumatoid arthritis-associated interstitial lung disease: a retrospective, active-comparator, new-user, cohort study. *The Lancet Rheumatology* 2025;7(3):e166-e77. doi: doi:10.1016/S2665-9913(24)00265-0
38. Fernandez-Diaz C, Castaneda S, Melero-Gonzalez RB, et al. Abatacept in interstitial lung disease associated with rheumatoid arthritis: national multicenter study of 263 patients. *Rheumatology (Oxford, England)* 2020;59(12):3906-16. doi: doi:https://dx.doi.org/10.1093/rheumatology/keaa621
39. Fernandez-Diaz C, Atienza-Mateo B, Castaneda S, et al. Abatacept in monotherapy vs combined in interstitial lung disease of rheumatoid arthritis-multicentre study of 263 Caucasian patients. *Rheumatology (Oxford, England)* 2021;61(1):299-308. doi: doi:https://dx.doi.org/10.1093/rheumatology/keab317
40. Fukuda W, Hanyu T, Katayama M, et al. Risk stratification and clinical course of hepatitis B virus reactivation in rheumatoid arthritis patients with resolved infection: Final report of a multicenter prospective observational study at Japanese Red Cross Hospital. *Arthritis Research and Therapy* 2019;21(1) doi: doi:10.1186/s13075-019-2053-1
41. Gremese E, Carletto A, Padovan M, et al. Obesity and reduction of the response rate to anti-tumor necrosis factor α in rheumatoid arthritis: an approach to a personalized medicine. *Arthritis Care Res (Hoboken)* 2013;65(1):94-100. doi: 10.1002/acr.21768
42. Haraoui B, Khraishi M, Choquette D, et al. Tofacitinib Safety and Effectiveness in Canadian Patients with Rheumatoid Arthritis by Cardiovascular Risk Enrichment: Subanalysis of the CANTORAL Study. *Rheumatology and Therapy* 2024;11(6):1629-48. doi: doi:10.1007/s40744-024-00719-5
43. Hoshida Y, Tsujii A, Ohshima S, et al. Effect of Recent Antirheumatic Drug on Features of Rheumatoid Arthritis-Associated Lymphoproliferative Disorders. *Arthritis & rheumatology (Hoboken, NJ)* 2024;76(6):869-81. doi: doi:https://dx.doi.org/10.1002/art.42809
44. Honne K, Bando M, Mieno MN, et al. Bronchiectasis is as crucial as interstitial lung disease in the severe pneumonia that occurs during treatment with biologic DMARDs in rheumatoid

arthritis: a retrospective cohort study in a single facility. *Rheumatology International* 2022;42(8):1341-46. doi: doi:10.1007/s00296-021-04934-z
45. Jeong W, Choe JY, Song BC, et al. Effect of low-dose corticosteroid use on hbv reactivation in hbsag-positive rheumatoid arthritis patients. *Open Rheumatology Journal* 2021;15(1):39-44. doi: doi:10.2174/1874312902115010039
46. Jeong S, Choi S, Park SM, et al. Incident and recurrent herpes zoster for first-line bDMARD and tsDMARD users in seropositive rheumatoid arthritis patients: a nationwide cohort study. *Arthritis Research and Therapy* 2022;24(1) doi: doi:10.1186/s13075-022-02871-1
47. Jung SM, Han M, Kim EH, et al. Comparison of developing tuberculosis following tumor necrosis factor inhibition and interleukin-6 inhibition in patients with rheumatoid arthritis: a nationwide observational study in South Korea, 2013-2018. *Arthritis research & therapy* 2022;24(1):157. doi: doi:https://dx.doi.org/10.1186/s13075-022-02842-6
48. Kalyoncu U, Bilgin E, Erden A, et al. Effectiveness and safety of tofacitinib in rheumatoid arthritis-associated interstitial lung disease: TReasure real-life data. *Clinical and Experimental Rheumatology* 2022;40(11):2071-77. doi: doi:10.55563/clinexprheumatol/9h6dtb
49. Kang EH, Jin Y, Desai RJ, et al. Risk of exacerbation of pulmonary comorbidities in patients with rheumatoid arthritis after initiation of abatacept versus TNF inhibitors: A cohort study. *Seminars in Arthritis and Rheumatism* 2020;50(3):401-08. doi: doi:10.1016/j.semarthrit.2019.11.010
50. Kao CM, Chen YJ, Chen YM, et al. Major adverse cardiovascular events or venous thromboembolism in patients with rheumatoid arthritis initiating biological or targeted synthetic disease-modifying antirheumatic drugs: a nationwide, population-based cohort study. *Therapeutic Advances in Musculoskeletal Disease* 2025;17 doi: doi:10.1177/1759720X251321917
51. Karataş A, Pişkin Sağir R, Koca SS, et al. Body mass index does not affect response of rituximab in patients with rheumatoid arthritis: results from the TURKBİO registry. *Turkish Journal of Medical Sciences* 2023;53(5):1321-29. doi: doi:10.55730/1300-0144.5698
52. Kelly CA, Nisar M, Arthanari S, et al. Rheumatoid arthritis related interstitial lung disease - Improving outcomes over 25 years: A large multicentre UK study. *Rheumatology (United Kingdom)* 2021;60(4):1882-90. doi: doi:10.1093/rheumatology/keaa577
53. Khosrow-Khavar F, Kim SC, Lee H, et al. Tofacitinib and risk of cardiovascular outcomes: results from the Safety of TofAcitinib in Routine care patients with Rheumatoid Arthritis (STAR-RA) study. *Annals of the rheumatic diseases* 2022;81(6):798-804.
54. Kim J-W, Shin K, Jung J, et al. Uncovering risk factors for adverse events and infections in rheumatoid arthritis and rheumatoid arthritis with interstitial lung disease under treatment with biologics or targeted synthetic DMARDs: insights from the KOBIO Registry. *Clinical and experimental rheumatology* 2024 doi: doi:https://dx.doi.org/10.55563/clinexprheumatol/6h1euo
55. Kim K, Woo A, Park Y, et al. Protective effect of methotrexate on lung function and mortality in rheumatoid arthritis-related interstitial lung disease: a retrospective cohort study. *Therapeutic advances in respiratory disease* 2022;16:17534666221135314. doi: doi:https://dx.doi.org/10.1177/17534666221135314
56. Kunishita Y, Ichikawa K, Uzawa Y, et al. Efficacy and safety of abatacept in patients with rheumatoid arthritis with previous malignancy. *Therapeutic Advances in Musculoskeletal Disease* 2023;15 doi: doi:10.1177/1759720X231186874
57. Kuo MH, Tseng C-W, Ko P-H, et al. HBV reactivation in HBsAg-/HBcAb+ rheumatoid arthritis patients receiving biologic/targeted synthetic DMARDs. *Liver international : official journal of the International Association for the Study of the Liver* 2024;44(2):497-507. doi: doi:https://dx.doi.org/10.1111/liv.15793
58. Kuo MH, Tseng C-W, Lee C-H, et al. Moderate Risk of Hepatitis B Virus Reactivation in HBsAg-/HBcAb+ Carriers Receiving Rituximab for Rheumatoid Arthritis. *Scientific reports* 2020;10(1):2456. doi: doi:https://dx.doi.org/10.1038/s41598-020-59406-4

59. Kuo MH, Tseng C-W, Lu M-C, et al. Risk of Hepatitis B Virus Reactivation in Rheumatoid Arthritis Patients Undergoing Tocilizumab-Containing Treatment. *Digestive diseases and sciences* 2021;66(11):4026-34. doi: doi:https://dx.doi.org/10.1007/s10620-020-06725-1
60. Kuwana M, Sugiyama N, Momohara S, et al. Three-year safety and effectiveness of tofacitinib in patients with rheumatoid arthritis in Japan: Final analysis of an all-case postmarketing surveillance study. *Modern rheumatology* 2025;35(4):637-49. doi: doi:https://dx.doi.org/10.1093/mr/roaf017
61. Lashen SA, Metawea MI, Shaaban A. Direct antiviral drugs in the treatment of hepatitis C-infected rheumatoid arthritis Egyptian cohort: Safety and clinical impact. *European Journal of Gastroenterology and Hepatology* 2021;33(1):E239-E46. doi: doi:10.1097/MEG.0000000000002015
62. Lee JS, Oh JS, Kim Y-G, et al. Methotrexate-related toxicity in patients with rheumatoid arthritis and renal dysfunction. *Rheumatology international* 2020;40(5):765-70. doi: doi:https://dx.doi.org/10.1007/s00296-020-04547-y
63. Lee S-K, Shin K, Jung J-Y, et al. Retention Rate and Safety of Biologic and Targeted Synthetic DMARDs in Patients with RA-Associated Interstitial Lung Disease: A KOBIO Registry Study. *BioDrugs : clinical immunotherapeutics, biopharmaceuticals and gene therapy* 2023;37(2):247-57. doi: doi:https://dx.doi.org/10.1007/s40259-023-00578-6
64. López-Maraver M, Serrano-Combarro A, Atienza-Mateo B, et al. Subcutaneous vs intravenous abatacept in rheumatoid arthritis-interstitial lung disease. National multicentre study of 397 patients. *Seminars in Arthritis and Rheumatism* 2024;68 doi: doi:10.1016/j.semarthrit.2024.152517
65. Maeyama A, Kondo M, Harada H, et al. Efficacy and safety of baricitinib in rheumatoid arthritis patients with moderate renal impairment: a multicenter propensity score matching study. *BMC Rheumatology* 2024;8(1) doi: doi:10.1186/s41927-024-00446-y
66. Md Yusof MY, Iqbal K, Darby M, et al. Effect of rituximab or tumour necrosis factor inhibitors on lung infection and survival in rheumatoid arthritis-associated bronchiectasis. *Rheumatology (United Kingdom)* 2020;59(10):2838-46. doi: doi:10.1093/rheumatology/kez676
67. Mena-Vazquez N, Rojas-Gimenez M, Fuego-Varela C, et al. Safety and Effectiveness of Abatacept in a Prospective Cohort of Patients with Rheumatoid Arthritis-Associated Interstitial Lung Disease. *Biomedicines* 2022;10(7) doi: doi:https://dx.doi.org/10.3390/biomedicines10071480
68. Mitsuboshi S. Risk of haematological events and preventive effect of folic acid in methotrexate users with chronic kidney disease and rheumatoid arthritis: Analysis of the Japanese Adverse Drug Event Report database. *British Journal of Clinical Pharmacology* 2021;87(5):2286-89. doi: doi:10.1111/bcp.14641
69. Mochizuki T, Yano K, Ikari K, et al. Three-year results of denosumab treatment for osteoporosis in women with rheumatoid arthritis and primary osteoporosis: A clinical observational study. *Modern rheumatology* 2021;31(3):600-06. doi: doi:https://dx.doi.org/10.1080/14397595.2020.1812793
70. Molina-Collada J, Alonso F, Otero L, et al. Cancer risk with biologic and targeted synthetic DMARDs in patients with rheumatic diseases and previous malignancies: Results from the BIOBADASER register. *Seminars in arthritis and rheumatism* 2024;64:152341. doi: doi:https://dx.doi.org/10.1016/j.semarthrit.2023.152341
71. Mori Y, Izumiyama T, Kurishima H, et al. Effect of denosumab switched from bisphosphonates in preventing joint destruction in postmenopausal rheumatoid arthritis patients with anti-cyclic citrullinated peptide antibodies. *Journal of orthopaedic surgery and research* 2021;16(1):107. doi: doi:10.1186/s13018-021-02271-2
72. Nakamura T, Higashi S-i, Tomoda K, et al. Effectiveness of etanercept vs cyclophosphamide as treatment for patients with amyloid A amyloidosis secondary to rheumatoid arthritis. *Rheumatology* 2012;51(11):2064-69.
73. Nakayama Y, Onishi A, Yamamoto W, et al. Safety of Janus kinase inhibitors compared to biological DMARDs in patients with rheumatoid arthritis and renal impairment: the ANSWER

cohort study. *Clinical and Experimental Medicine* 2024;24(1) doi: doi:10.1007/s10238-024-01360-w
74. Novella-Navarro M, Genre F, Hernandez-Breijo B, et al. Obesity and response to biological therapy in rheumatoid arthritis: the role of body mass index and adipose tissue cytokines. *Clinical and experimental rheumatology* 2022;40(9):1726-32. doi: doi:https://dx.doi.org/10.55563/clinexprheumatol/a9gskx
75. Padovan M, Filippini M, Tincani A, et al. Safety of abatacept in rheumatoid arthritis with serologic evidence of past or present hepatitis B virus infection. *Arthritis care & research* 2016;68(6):738-43.
76. Pappas DA, Etzel CJ, Crabtree M, et al. Effectiveness of tocilizumab in patients with rheumatoid arthritis is unaffected by comorbidity burden or obesity: Data from a US registry. *Journal of Rheumatology* 2020;47(10):1464-74. doi: doi:10.3899/jrheum.190282
77. Priora M, Becciolini A, Celletti E, et al. Effectiveness and Predictors of Long-Term Treatment Response to Tofacitinib in Rheumatoid Arthritis Cohort: General Analysis and Focus on High-Cardiovascular-Risk Subgroup-A Multicenter Study. *Medicina (Kaunas, Lithuania)* 2024;60(12) doi: doi:10.3390/medicina60121982
78. Pundole X, Zamora NV, Siddhanamatha H, et al. Overall survival in patients with rheumatoid arthritis and solid malignancies receiving biologic disease-modifying antirheumatic therapy. *Clinical Rheumatology* 2020;39(10):2943-50. doi: doi:10.1007/s10067-020-05318-7
79. Rudi T, Zietemann V, Meissner Y, et al. Impact of DMARD treatment and systemic inflammation on all-cause mortality in patients with rheumatoid arthritis and interstitial lung disease: a cohort study from the German RABBIT register. *RMD open* 2024;10(2) doi: doi:https://dx.doi.org/10.1136/rmdopen-2023-003789
80. Ruiz JI, Lei X, Chi-Fang W, et al. Survival in patients with rheumatoid arthritis and early breast cancer treated with tumor necrosis factor inhibitors. *Breast Cancer* 2024 doi: doi:10.1007/s12282-024-01618-x
81. Ruiz JI, Lei X, Giordano SH, et al. Survival in patients with rheumatoid arthritis and recently diagnosed early-stage colorectal, lung, or prostate cancer receiving tumour necrosis factor inhibitors: a retrospective cohort study. *The Lancet Rheumatology* 2025;7(5):e333-e42. doi: doi:10.1016/S2665-9913(24)00379-5
82. Sagtaganov Z, Yessirkepov M, Bekaryssova D, et al. Managing rheumatoid arthritis and cardiovascular disease: the role of physical medicine and rehabilitation. *Rheumatology International* 2024;44(9):1749-56. doi: doi:10.1007/s00296-024-05651-z
83. Schäfer M, Meißner Y, Kekow J, et al. Obesity reduces the real-world effectiveness of cytokine-targeted but not cell-targeted disease-modifying agents in rheumatoid arthritis. *Rheumatology* 2020;59(8):1916-26.
84. Serrano-Combarro A, Atienza-Mateo B, Martín-Gutiérrez A, et al. Baricitinib in rheumatoid arthritis-interstitial lung disease: national multicenter study of 72 patients and literature review. *Rheumatology (Oxford, England)* 2025 doi: doi:10.1093/rheumatology/keaf314
85. Shen X, Wang F. The additional treatment value of immunoglobulin for the treatment of rheumatoid arthritis complicated with interstitial lung disease: A propensity score-matched pilot study. *International journal of rheumatic diseases* 2023;26(9):1745-50. doi: doi:https://dx.doi.org/10.1111/1756-185X.14808
86. Shih P-C, Zou Q-H, Lai C-C, et al. Mortality and medical utilization in rheumatoid arthritis associated interstitial lung disease: A real-world, large-scale retrospective study comparing Janus kinase inhibitors and tumor necrosis factor inhibitors. *Seminars in arthritis and rheumatism* 2025;71:152636. doi: https://dx.doi.org/10.1016/j.semarthrit.2025.152636
87. Shin A, Lee YJ, Lee EB, et al. Tuberculosis risk with biologics by screening-guided preventive strategy in rheumatoid arthritis under intermediate tuberculosis burden. *Rheumatology (Oxford, England)* 2021;60(6):2755-64. doi: doi:https://dx.doi.org/10.1093/rheumatology/keaa702

88. Shu P, Shao SQ, Cai XN, et al. Iguratimod attenuates general disease activity and improves lung function in rheumatoid arthritis-associated interstitial lung disease patients. *European Review for Medical and Pharmacological Sciences* 2021;25(14):4687-92. doi: doi:10.26355/eurrev_202107_26379
89. Specker C, Aringer M, Burmester G-R, et al. The safety and effectiveness of tocilizumab in elderly patients with rheumatoid arthritis and in patients with comorbidities associated with age. *Clinical and experimental rheumatology* 2022;40(9):1657-65. doi: doi:https://dx.doi.org/10.55563/clinexprheumatol/f7ff6q
90. Comparative safety of abatacept in rheumatoid arthritis with COPD: a real-world population-based observational study. Seminars in arthritis and rheumatism; 2019. Elsevier.
91. Tardella M, Di Carlo M, Carotti M, et al. A retrospective study of the efficacy of JAK inhibitors or abatacept on rheumatoid arthritis-interstitial lung disease. *Inflammopharmacology* 2022;30(3):705-12. doi: doi:10.1007/s10787-022-00936-w
92. Triboulet F, Juge PA, Truchetet ME, et al. Evaluation of rheumatoid arthritis-associated interstitial lung disease in patients treated with JAK inhibitors: a MAJIK-SFR cohort study. *RMD Open* 2025;11(1) doi: doi:10.1136/rmdopen-2024-005062
93. Tsuboi H, Toko H, Honda F, et al. Abatacept ameliorates both glandular and extraglandular involvements in patients with Sjogren's syndrome associated with rheumatoid arthritis: Findings from an open-label, multicentre, 1-year, prospective study: The ROSE (Rheumatoid Arthritis with Orencia Trial Toward Sjogren's Syndrome Endocrino. *Modern rheumatology* 2023;33(1):160-68. doi: doi:https://dx.doi.org/10.1093/mr/roac011
94. Vallejo-Yagüe E, Burkard T, Finckh A, et al. Comparative effectiveness of biologics in patients with rheumatoid arthritis stratified by body mass index: a cohort study in a Swiss registry. *BMJ Open* 2024;14(2) doi: doi:10.1136/bmjopen-2023-074864
95. Venerito V, Manfredi A, Carletto A, et al. Evolution of Rheumatoid-Arthritis-Associated Interstitial Lung Disease in Patients Treated with JAK Inhibitors: A Retrospective Exploratory Study. *Journal of Clinical Medicine* 2023;12(3) doi: doi:10.3390/jcm12030957
96. Wang S, Li Y, Tang Y, et al. A prospective observational cohort study of the efficacy of tofacitinib plus iguratimod on rheumatoid arthritis with usual interstitial pneumonia. *Frontiers in Immunology* 2023;14 doi: doi:10.3389/fimmu.2023.1215450
97. Westermann R, Cordtz R, Duch K, et al. Cancer recurrence risk with bDMARD treatment in patients with rheumatoid arthritis and a history of cancer: a nationwide Danish register-based cohort study. *RMD Open* 2025;11(2) doi: doi:10.1136/rmdopen-2024-005247
98. Yokoyama S, Ishii Y, Masuda J. Persistence and Safety of Golimumab in Elderly Patients with Rheumatoid Arthritis and Renal Dysfunction in a Real-World Setting. *Drugs - Real World Outcomes* 2023;10(1):51-60. doi: doi:10.1007/s40801-022-00338-y
99. Yoshimura Y, Yamanouchi M, Mizuno H, et al. Efficacy and safety of first-line biological DMARDs in rheumatoid arthritis patients with chronic kidney disease. *Annals of the Rheumatic Diseases* 2024 doi: doi:10.1136/ard-2024-225914
100. Yoshimura Y, Yamanouchi M, Koizumi R, et al. Efficacy and safety of first-line targeted synthetic DMARDs in rheumatoid arthritis patients with chronic kidney disease. *Rheumatology* 2025;64(6):3396-404. doi: doi:10.1093/rheumatology/keaf050
101. You S-H, Cho S-K, Kim J-Y, et al. Risk of major adverse cardiovascular events following targeted therapy in patients with rheumatoid arthritis: a real-world analysis stratified by cardiovascular risk. *Seminars in arthritis and rheumatism* 2025;73:152721. doi: https://dx.doi.org/10.1016/j.semarthrit.2025.152721
102. Gupta P, Lanca C, Gan AT, et al. The association between body composition using dual energy X-ray absorptiometry and type-2 diabetes: a systematic review and meta-analysis of observational studies. *Scientific reports* 2019;9(1):12634.
